# Horizontal gene transfer and gene loss drove the divergent evolution of host dependency in Micrarchaeota

**DOI:** 10.1093/nsr/nwaf542

**Published:** 2025-11-28

**Authors:** Yang-Zhi Rao, Yu-Xian Li, Ze-Wei Li, Yan-Ni Qu, Brian P Hedlund, Tom A Williams, Yan-Ling Qi, Qi-Jun Xie, Hai-Long Yang, Yuan-Qi Zhang, Hong-Chen Jiang, Marike Palmer, Mang Shi, Wen-Sheng Shu, Zheng-Shuang Hua, Wen-Jun Li

**Affiliations:** State Key Laboratory of Biocontrol, Guangdong Provincial Key Laboratory of Plant Resources and Southern Marine Science and Engineering Guangdong Laboratory (Zhuhai), School of Life Sciences, Sun Yat-Sen University, Guangzhou 510275, China; State Key Laboratory of Advanced Environmental Technology, Department of Environmental Science and Engineering, University of Science and Technology of China, Hefei 230026, China; Centre for Infection and Immunity Study (CIIS), School of Medicine (Shenzhen), Sun Yat-sen University, Shenzhen 518107, China; State Key Laboratory of Advanced Environmental Technology, Department of Environmental Science and Engineering, University of Science and Technology of China, Hefei 230026, China; Southern Marine Science and Engineering Guangdong Laboratory (Guangzhou), Guangzhou 511458, China; State Key Laboratory of Advanced Environmental Technology, Department of Environmental Science and Engineering, University of Science and Technology of China, Hefei 230026, China; State Key Laboratory of Advanced Environmental Technology, Department of Environmental Science and Engineering, University of Science and Technology of China, Hefei 230026, China; School of Life Sciences, University of Nevada Las Vegas, Las Vegas, NV 89154, USA; Department of Life Sciences, University of Bath, Bath BA2 7AX, UK; State Key Laboratory of Advanced Environmental Technology, Department of Environmental Science and Engineering, University of Science and Technology of China, Hefei 230026, China; State Key Laboratory of Advanced Environmental Technology, Department of Environmental Science and Engineering, University of Science and Technology of China, Hefei 230026, China; State Key Laboratory of Advanced Environmental Technology, Department of Environmental Science and Engineering, University of Science and Technology of China, Hefei 230026, China; State Key Laboratory of Advanced Environmental Technology, Department of Environmental Science and Engineering, University of Science and Technology of China, Hefei 230026, China; Geomicrobiology Laboratory, State Key Laboratory of Geological Processes and Mineral Resources, China University of Geosciences, Beijing 100083, China; Department of Microbiology, University of Manitoba, Manitoba R3T 2N2, Canada; Centre for Infection and Immunity Study (CIIS), School of Medicine (Shenzhen), Sun Yat-sen University, Shenzhen 518107, China; School of Life Sciences, South China Normal University, Guangzhou 510275, China; State Key Laboratory of Advanced Environmental Technology, Department of Environmental Science and Engineering, University of Science and Technology of China, Hefei 230026, China; State Key Laboratory of Biocontrol, Guangdong Provincial Key Laboratory of Plant Resources and Southern Marine Science and Engineering Guangdong Laboratory (Zhuhai), School of Life Sciences, Sun Yat-Sen University, Guangzhou 510275, China; State Key Laboratory of Desert and Oasis Ecology, Xinjiang Key Laboratory of Biodiversity Conservation and Application in Arid Lands, Xinjiang Institute of Ecology and Geography, Chinese Academy of Sciences, Urumqi 830011, China

**Keywords:** DPANN, metabolic capacity, horizontal gene transfer, evolutionary history, reductive evolution

## Abstract

The DPANN superphylum is a deep-branching radiation of archaea with small cell and genome sizes. Most DPANN lineages are predicted or validated to be host-dependent. However, certain lineages have substantial biosynthetic capacities and are potentially less dependent on hosts, or even free-living. Here, we reconstructed 163 Micrarchaeota genomes, comprising 48 assigned to previously undescribed orders and 115 affiliated with known orders. Investigation of their genetic repertoire revealed substantial metabolic capacity in Norongarragalinales-, Anstonellales- and the newly proposed Wunengiarchaeales-associated lineages, including complete or near-complete glycolysis and *de novo* biosynthetic pathways for nucleotides, amino acids, cofactors and cell envelopes. We classified genes related to the central metabolism but which are uncommon in DPANN archaea as putative free-living associated genes (pFLAGs). The extensive presence of pFLAGs in Norongarragalinales suggests a potential host-independent lifestyle. Reconstruction of evolutionary history revealed that these pFLAGs were not ancestral within the DPANN superphylum. Instead, we suggest that less-host-dependent organisms evolved from symbionts through the gradual acquisition of pFLAGs through horizontal gene transfer, whereas other Micrarchaeota lineages with streamlined genomes experienced reductive evolution due to thermal adaptation. Our analyses demonstrate that host dependency is not always an evolutionary dead end, but can be reversed through the acquisition of new metabolic capabilities by horizontal transfer.

## INTRODUCTION

Archaea is one of the major lineages of life and plays pivotal roles in Earth’s biogeochemical cycles [1,2]. Among archaea, the ‘DPANN superphylum’ is characterized by their diminutive cell and genome sizes [3,4]. They were originally proposed to unite Diapherotrites, Parvarchaeota, Aenigmarchaeota, Nanoarchaeota and Nanohaloarchaeota (reclassified as Iainarchaeota, Aenigmarchaeota, Parvarchaeales, Nanoarchaeales and Nanohaloarchaeota in Genome Taxonomy Database (GTDB, r207)) [5–10] and have since expanded to contain additional archaeal lineages [11–15]. With a limited number of cultured representatives, cultivation-independent studies have recovered a large number of metagenome-assembled genomes (MAGs) and single-cell amplified genomes, providing additional insights into the phylogenetic diversity, metabolic capacities and ecological roles of these ultra-small archaea [10,11,16–19]. The DPANN archaea inhabit diverse ecosystems, including marine, freshwater,

terrestrial and animal microbiomes, and extreme environments, such as high-temperature, hypersaline, acidic and alkaline surroundings [3,4,20].

Given their small genome sizes and limited metabolic potentials, DPANN archaea are generally considered to have an obligately symbiotic lifestyle [3,4,21]. This host-dependent relationship was shown by using multiple laboratory co-cultivation experiments [6,22–25]. Intriguingly, some other studies have suggested a departure from the traditional view that all DPANN archaea are obligate symbionts [11,17,18,21]. Genomes from members of Aenigmarchaeota, Iainarchaeota, Micrarchaeota, Parvarchaeales and Woesearchaeales have been reported to encode pathways to synthesize macromolecular building blocks, such as membrane lipids, carbohydrates, amino acids, nucleotides and cofactors. Additionally, some Micrarchaeota, Parvarchaeales and Nanohaloarchaeota genomes encode complete pathways for glycolysis, the tricarboxylic acid (TCA) cycle and/or electron transport chains (ETCs) involved in carbon metabolism and energy conservation [18,26,27]. Earlier studies on Iainarchaeota also suggest a potential host-independent lifestyle by the acquisition of these pathways via horizontal gene transfer (HGT) [11,17,28]. Additionally, Altiarchaeota genomes encode a modified Wood–Ljungdhal pathway (WLP) that may enable autotrophic growth [29] and they may even serve as hosts for Huberarchaeota [13,14,30]. The variable existence of these core pathways among DPANN archaea raises the question of whether the symbiotic lifestyle of some DPANN archaea is ancestral or a result of reductive evolution from a free-living ancestor.

As one of the earliest-discovered DPANN lineages, Micrarchaeota were shown to inhabit diverse environments, with putative hosts belonging to the Thermoplasmatales and Sulfolobales [7,11,18,23–25,31]. Here, we applied a genome-resolved metagenomic approach with samples from geothermal springs and described multiple new lineages within Micrarchaeota. We show that Micrarchaeota encode wide variations in pathways associated with central metabolism, energy conservation and membrane biosynthesis. We then used a comprehensive comparative genomics approach to examine the distribution of these ‘putative free-living associated genes (pFLAGs)’ among Micrarchaeota genomes and across other DPANN lineages, revealing that several Micrarchaeota lineages, especially Norongarragalinales, encode comparable metabolic capacity to those of Altiarchaeota and other free-living archaea. In contrast, other Micrarchaeota have highly reduced genomes and exhibit extreme metabolic deficiency. Finally, by reconstructing their evolutionary history, we show that HGT drove the gradual acquisition of pFLAGs in some lineages to evolve from obligate symbiotic ancestors. We also show that gene loss accelerated within the Anstonellales, giving rise to the most reduced archaeal genomes in a new genus that we designate as Redboyarchaeum. Our work highlights divergent evolution within the Micrarchaeota and predicts that these different evolutionary trajectories led to different degrees of host dependency.

## RESULTS

### Expansion of genomic and phylogenomic diversity of Micrarchaeota

**Figure 1.**
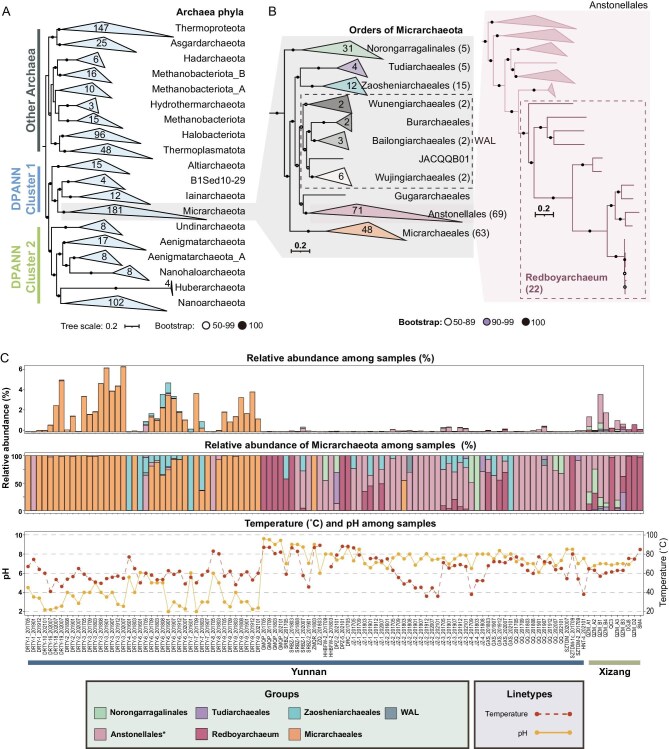
Phylogenetic placement of reconstructed Micrarchaeota MAGs. (A) Maximum-likelihood phylogeny of all 163 Micrarchaeota MAGs and reference genomes based on 53 concatenated GTDB conserved marker genes showed the topology and placement of Micrarchaeota among all archaeal phyla, with the numbers within each clasped clade representing the number of genomes of each phylum. Nodes with 100 bootstrap supports are represented by solid circles and bootstrap supports with between 50% and 99% are represented by hollow circles. (B) Expanded phylogenetic tree of Micrarchaeota branch in (A) showing the detailed phylogenetic placement of all 163 MAGs in this study and those of other Micrarchaeota reference genomes. The numbers within each clade represent the number of genomes of each phylum shown in the phylogenetic tree, while the numbers in parentheses indicate the number of genomes recovered in this study. The hollow, purple and solid circles represent the bootstrap values of 50%–89%, 90%–99% and 100%, respectively. The ‘WAL’ label represents Wunengiarchaeales-associated lineages. The Anstonellales order was further expanded to show the phylogenetic position of the genome-streamlined genus *Redboyarchaeum*. (C) Relative abundance of different major Micrarchaeota lineages and the pH and temperature of sample sites in Yunnan and Xizang. The relative abundances of different groups of Micrarchaeota among all samples are shown at the top with bars of different colors. The proportions of different groups among Micrarchaeota are shown in the middle. The pH and temperature of each sample site are shown at the bottom. The red dashed line represents the temperature values and the orange line represents the pH values.

A total of 163 Micrarchaeota MAGs were recovered from metagenomes derived from 160 geothermal spring samples from Yunnan Province and Xizang Autonomous Region, China, that were collected from January 2016 to January 2021 in 59 unique sampling locations (see details in Datasets S1 and S2 and Supporting information). Genome completeness assessment based on 48 single-copy genes [11,32] (Dataset S3) and contamination assessment based on CheckM [33] revealed that these genomes were of high or medium quality, with ≥50% completeness and <5% contamination, including 91 genomes with completeness of ≥90% and contamination of <3% (Table 1 and Dataset S1). Phylogenomic analyses of 68 species-dereplicated representative MAGs and 658 reference genomes based on 53 archaeal markers that were specifically examined as being top-ranked on archaea, especially taking into consideration the reduced genomes of DPANN, were conducted (see details in the ‘Methods’ section) [15]. This phylogeny is congruent with those of previous studies and recovers the two major clusters of DPANN archaea (DPANN Cluster 1 and 2) and shows robust support for the placement of these MAGs within Micrarchaeota and the placement of Micrarchaeota within DPANN Cluster 1 (Fig. 1A and Datasets S4 and S5; the robustness of phylogenomic inference is further examined in the ‘Evolutionary mechanisms driving the acquisition of pFLAGs in Micrarchaeota’ section). Within Micrarchaeota, 11 order-level lineages were identified and each order-level lineage was supported by high bootstrap values (Fig. 1B). Among the 163 MAGs recovered in this study, 5, 47 and 63 were assigned to Norongarragalinales, Anstonellales and Micrarchaeales, respectively [31]. In addition to these previously described orders, we propose five newly discovered or yet-to-be-described orders (Dataset S1; see details in the Nomenclature Appendix of the Supporting information) and we propose the following names under the SeqCode [34]: Tudiarchaeales (5 genomes, CAILAH01 in GTDB r207), Zaosheniarchaeales (15 genomes, CABMEP01), Wunengiarchaeales (2 genomes, new order), Bailongiarchaeales (2 genomes, JACRGF01) and Wujingiarchaeales (2 genomes, DTNL01). Wunengiarchaeales, Burarchaeales, Bailongiarchaeales, JACQQB01 and Wujingiarchaeales form a monophyletic lineage with relatively high average amino-acid identities (Fig. S1), yet each is represented by a small number of genomes (up to six) (Fig. 1B). Herein, we describe them informally as Wunengiarchaeales-associated lineages (WAL) and refer to WAL and other orders as ‘major lineages of Micrarchaeota’ to avoid ambiguity. We also recovered 22 new genomes representing a genus of Anstonellales; this genus has the smallest known genome sizes among all DPANN archaea [3] and we propose the name Redboyarchaeum (genus JAADEL01 in GTDB r207).

**Table 1.** Genomic features of 163 Micrarchaeota bins from the metagenomic assembly of this study.

Lineages	Norongarragalinales	Tudiarchaeales	Zaosheniarchaeales	WAL	Anstonellales	Redboyarchaeum	Micrarchaeales
Number of genomes	5	5	15	6	47	15	63
Genome sizes (Mbp)	0.93–0.73 (0.85)	0.92–0.48 (0.69)	0.98–0.52 (0.75)	1.16–0.65 (1.06)	1.20–0.49 (0.84)	0.77–0.33 (0.51)	0.94–0.34 (0.62)
GC content (%)	51.90–36.50 (48.61)	52.07–37.32 (44.00)	43.25–32.18 (33.10)	59.94–43.08 (44.44)	51.98–29.85 (40.03)	41.13–26.11 (34.38)	48.64–28.27 (45.61)
No. of scaffolds	105–9 (56)	86–7 (41)	96–8 (38)	171–6 (44.50)	238–5 (35)	88–5 (42.50)	161–18 (66)
N50 (bp)	71 4187–8277 (22 882)	154 525–6220 (19 069)	138 651–8909 (28 609)	541 696–3762 (32 596.50)	881 330–4873 (32 503)	124 158–6166 (15 215.50)	52 186–4052 (11 506)
Completeness (%)	97.92–66.67 (91.67)	97.92–52.08 (79.17)	100.00–60.42 (91.67)	100.00–83.33 (96.88)	100.00–58.33 (95.83)	100.00–52.08 (91.67)	97.92–66.67 (89.58)
Contamination (%)	1.87–0.00 (1.01)	2.34–0.93 (0.93)	4.67–0.00 (0.54)	1.87–0.00 (0.00)	2.25–0.00 (0.00)	1.01–0.00 (0.00)	4.67–0.00 (0.00)
5S rRNA	1–0	0–0	1–0	1–0	2–0	1–0	2–0
16S rRNA	2–0	2–0	1–0	2–0	2–0	2–0	3–0
23S rRNA	2–0	1–0	1–0	1–0	2–0	1–0	3–0
tRNA	47–31 (40)	41–28 (36)	47–26 (35)	46–29 (37)	67–23 (39)	43–22 (34)	46–17 (36)
Number of proteins	997–889 (921)	916–556 (741)	1008–554 (746)	1208–796 (1191)	1358–565 (877)	896–409 (589)	1015–416 (697)
Average length (bp)	880.84–731.28 (819.27)	927.13–775.50 (866.61)	920.22–739.17 (862.05)	912.15–772.57 (841.51)	957.47–766.94 (872.31)	929.13–761.15 (836.61)	860.82–728.38 (816.19)
Coding density (%)	92.52–87.58 (91.35)	93.16–92.16 (92.53)	91.74–85.52 (89.99)	94.98–93.63 (94.51)	94.88–86.29 (93.08)	97.03–90.26 (95.51)	93.76–87.35 (91.56)
Overlapped genes (%)	15.50–8.32 (11.84)	16.39–8.52 (9.85)	11.07–3.35 (4.86)	24.22–16.33 (19.55)	25.54–8.71 (16.88)	42.55–27.57 (39.00)	22.71–6.98 (12.18)

General genome statistics of major lineages of Micrarchaeota analysed in this study, including the number of genomes, genome sizes, GC content, assembly statistics, genome qualities, presence of RNA genes and statistics of protein-coding genes. Ranges represent the maximum and minimum of the respective characteristics of a specific lineage, medium values are shown in parentheses and statistics in detail are listed in Dataset S1. The genomic completeness was assessed by 48 conserved archaeal single-copy genes presented in each MAG and the contamination was measured by using CheckM. WAL, Wunengiarchaeales-associated lineages.

Micrarchaeota have small but highly variable genome sizes (Fig. S2A). Among Micrarchaeota, Zaosheniarchaeales (0.52–0.98 Mbp, median = 0.76 Mbp) and Micrarchaeales (0.34–1.06 Mbp, median = 0.69 Mbp) possess significantly reduced genomes compared with Norongarragalinales (0.65–1.37 Mbp, median = 0.93 Mbp), WAL (0.65–1.51 Mbp, median = 1.07 Mbp) and Anstonellales (Fig. S3A, 0.49–1.59 Mbp, median = 0.90 Mbp). This genome-size variation is driven by differences in the number of genes rather than variations in gene lengths (Fig. S3B and C), as revealed by a positive relationship between the genome size and the number of genes (Fig. S2B, *R*^2^_adj_ = 0.92, *P* < 0.001). Both Zaosheniarchaeales (32.18%–43.25%, median = 33.11%) and Redboyarchaeum (26.11%–41.13%, median = 34.40%) had ultra-low GC content (Fig. S3D). Genomes of Redboyarchaeum were characterized by having extremely small genome sizes (Fig. S3A, 0.33–0.77 Mbp, median = 0.52 Mbp), high coding densities (90.26%–97.03%, median = 95.56%) and high percentages of overlapping genes (Fig. S3E and F, 27.57%–42.55%, median = 39.00%). Genomes of Zaosheniarchaeales, although small (Fig. S3A, genome-size range: 0.52–0.98 Mbp, median = 0.76 Mbp), had relatively low coding density (85.52%–91.74%, median = 90.04%) and percentages of overlapping genes (3.35%–11.07%, median = 4.50%). We further investigated the factors that drove proteome differences between these lineages. T-distributed stochastic neighbor embedding analyses based on the archaeal clusters of orthologous groups (arCOGs) (Fig. S4A and B) and KEGG Orthology (Fig. S4C and D) of all Micrarchaeota genomes showed that they clustered better by phylogenetic distance than the derived environments.

Micrarchaeales and Zaosheniarchaeales were relatively abundant in acidic hot springs (Fig. 1C and Datasets S2 and S6). Micrarchaeales were enriched among sites with low pH (mostly pH ≤ 4) and non-hyperthermal temperature (mostly <70˚C). Zaosheniarchaeales were more abundant in springs that were slightly acidic (pH of ≥5 but <7). Statistics of the sampling pH of Micrarchaeales were also shown to be significantly lower (Fig. S3G, 1.0–9.6, median = 5.85), confirming this order as acidophilic. In many of these springs, their cumulative relative abundance was >1%, with a maximum abundance of >6% (DRTY-3_201901 and DRTY-3_202007), representing a non-negligible component of the communities. Norongarragalinales, Tudiarchaeales, WAL and Anstonellales were present at low relative abundance (<1%) among all samples, which were mostly with pH ≥ 6. Interestingly, Redboyarchaeum was the dominant Micrarchaeota genus among hyperthermal sites (most with temperature of ≥80˚C). Among all Micrarchaeota orders, genomes of Tudiarchaeales and Zaosheniarchaeales appeared to be mostly derived from thermal environments with temperatures of between 60˚C and 80˚C (Fig. 1B). Norongarragalinales, WAL and Anstonellales were obtained from diverse habitats that included both extreme and more temperate environments. Redundancy analysis based on the physicochemical parameters of the sampling sites confirmed pH as the most determinant factor influencing Micrarchaeota distribution, with total organic matter (TOM), phosphorus and temperature also playing key roles (Fig. S4E and Dataset S2). The negative relationship between temperature and Micrarchaeales consolidates their preferential distribution in lower-temperature environments. When considering all Micrarchaeota genomes, their amino-acid compositions differ, reflecting distinct optimal growth temperatures (OGTs) and varying capacities to grow in high-temperature environments. Redboyarchaeum had higher predicted OGTs compared with other lineages, while Norongarragalinales and Micrarchaeales OGTs were lower (Fig. S3H and I). A significant negative correlation exists between the predicted OGTs and genome sizes of the major Micrarchaeota lineages consisting of Norongarragalinales, Tudiarchaeales, Zaosheniarchaeales, WAL and Anstonellales (including Redboyarchaeum, Fig. S2C, *R*^2^_adj_ = 0.60, *P* < 0.001). This relationship was particularly strong among Anstonellales (Fig. S2D, *R*^2^_adj_ = 0.70, *P* < 0.001). Further validation of the relationship between OGTs and sampling temperatures corroborated the amino-acid-bias-based criteria (Fig. S2E, *R*^2^_adj_ = 0.46, *P* < 0.001; Fig. S2F, *R*^2^_adj_ = 0.48, *P* < 0.001; Fig. S2G, *R*^2^_adj_ = 0.58, *P* < 0.001; Fig. S2H, *R*^2^_adj_ = 0.55, *P* < 0.001). Taken together, temperature and pH have a pronounced effect on the distribution of the major lineages of Micrarchaeota, with predicted hyperthermophiles often possessing streamlined genomes.

### Metabolic potential of Micrarchaeota and reduced host dependency via pFLAGs

**Figure 2.**
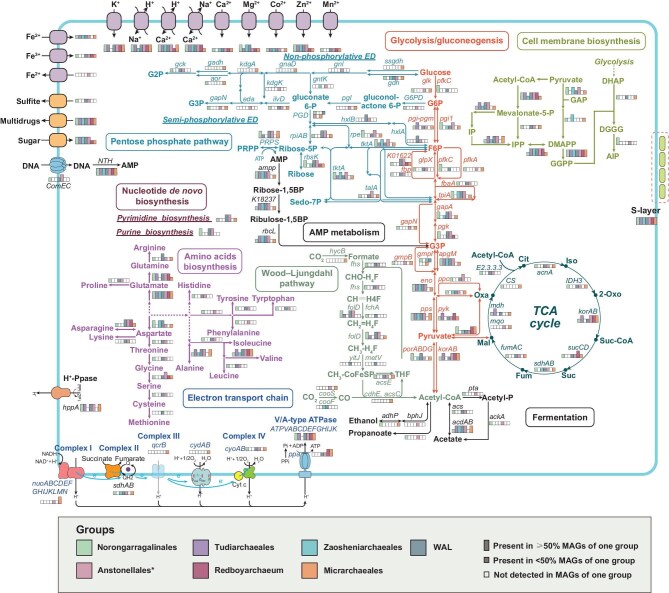
Overview of metabolic potentials of Micrarchaeota. The presence of genes in different major lineages of Micrarchaeota is shown by using different color symbols, respectively. Genes that are present in ≥50% of the genomes are illustrated by using complete rectangles, genes present in <50% but at least one MAG are represented by half rectangles and genes that were not detected in one group are represented by hollow half rectangles. Different metabolic pathways are represented by lines of different colors. Asterisks indicate that Redboyarchaeum were excluded from the statistics of other Anstonellales genomes. G6P, glucose-6-phosphate; F6P, fructose-6-phosphate; G3P, glycerate-3-phosphate; G2P, glycerate-2-phosphate; Ribose-5P, ribose-5-phosphate; Sedo-7P, sedoheptulose-7-phosphate; PRPP, phosphoribosyl diphosphate; gluconate 6-P, 6-phosphogluconate; gluconolactone 6-P, 6-phosphogluconolactone; Mevalonate-5-P, mevalonate phosphate; IP, isopentenyl phosphate; IPP, isopentenyl diphosphate; DMAPP, dimethylallyl pyrophosphate; GGPP, geranylgeranyl diphosphate; DHAP, dihydroxyacetone phosphate; DGGGP, digeranylgeranylglyceryl phosphate; AIP, archaetidylinositol phosphate; Oxa, oxaloacetate; Cit, citrate; Iso, isocitrate; 2-Oxo, 2-oxoglutarate; Suc-CoA, succinyl–CoA; Suc, succinate; Fum, fumarate; Mal, malate.

**Figure 3.**
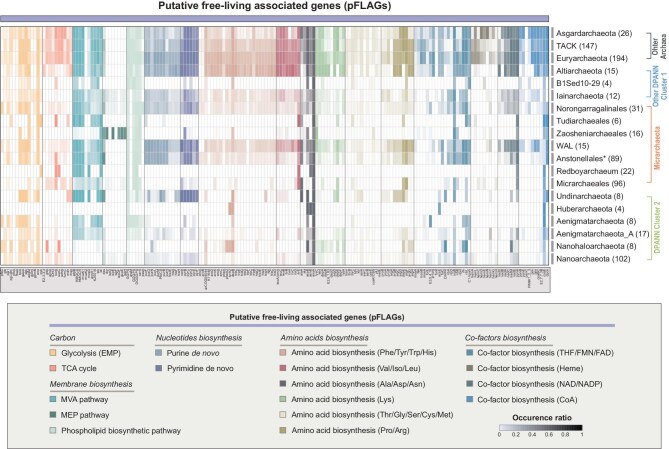
Presence of key genes of interest in Micrarchaeota and other archaea lineages. Each row represents a MAG that is ordered by the same phylogenetic placement as shown in Fig. 2. Each column indicates a gene or a set of genes of the same functions associated with putative free-living associated genes (pFLAGs). The transparency of each block indicates the occurrence ratio of each gene on associated lineages. The ‘WAL’ label represents Wunengiarchaeales-associated lineages. Genes of listing occurrences can be found in Dataset S8.

Based on the functional annotation (see the ‘Methods’ section for details) of 276 Micrarchaeota genomes, including 163 genomes recovered in this study and 113 reference genomes, we reconstructed metabolic pathways in members of the phylum Micrarchaeota (Fig. 2 and Dataset S7). The widespread deficiency of complete central carbon metabolic pathways (e.g. glycolysis, TCA cycle), the ETC and anabolic pathways such as the *de novo* biosynthesis of archaeal cell membranes, nucleotides, amino acids and cofactors was confirmed. These metabolic deficiencies are part of the evidence suggesting that DPANN archaea are obligate symbionts [3,4,11]. In particular, the lack of pathways for *de novo* cell membrane biosynthesis is strong evidence of host dependency [3]. Our analysis confirmed the ‘typical’ metabolisms of DPANN in most orders, which are likely based on a fermentative ‘salvage’ lifestyle (Fig. 3). Relevant pathways include the non-oxidative pentose phosphate pathway (noPPP), the adenosine monophosphate pathway, the ETC, fermentation, hydrogenases and pathways associated with protein/peptide degradation and amino-acid import (Fig. 2, Figs S5A and B and S6, and Dataset S7; see Supplementary text for details). We also detected potential propanoate fermentation with a complete citramalate pathway encoded by Norongarragalinales, WAL and Anstonellales. In addition, Micrarchaeota also encoded numerous genes involved in sulfur and nitrogen metabolisms as well as CRISPR-Cas systems, similarly to previous findings (Fig. 2, Figs S6 and S7, and Dataset S7; see Supplementary text for details) [18]. On the other hand, our analyses indicate that Norongarragalinales, WAL, Anstonellales and Micrarchaeales have an expanded metabolic capability. These lineages encode pathways including a complete oxidative PPP and a branched Entner–Doudoroff (ED) glycolysis pathway encoded by Anstonellales; a reverse ribulose monophosphate pathway encoded by WAL and Anstonellales; a complete Embden–Meyerhof–Parnas glycolysis pathway utilizing bifunctional ADP-dependent phosphofructokinase/glucokinase (*pfkC*) encoded by Norongarragalinales and WAL; a complete TCA cycle encoded by Micrarchaeales and Norongarragalinales; and a partial WLP encoded by several Micrarchaeota orders.

Unexpectedly, we also found that certain Micrarchaeota not only have the abovementioned catabolic pathways, but also possess considerable biosynthetic capacity. Norongarragalinales, WAL, Anstonellales and Micrarchaeales have complete pathways for *de novo* pyrimidine biosynthesis (Fig. 2, Fig. S8A and Dataset S7). Norongarragalinales, WAL and Anstonellales also have complete pathways for *de novo* purine biosynthesis, while Micrarchaeales encode a partial pathway. The majority of Norongarragalinales, WAL and Anstonellales genomes have *purH* and *pyrF* genes involved with the biosynthesis of the key nucleotide intermediates inosine monophosphate and uridine monophosphate. Norongarragalinales, WAL, Anstonellales and Micrarchaeales have complete mevalonate (MVA) pathways for the biosynthesis of the key archaeal membrane lipid precursor isopentenyl diphosphate (IPP) via MVA kinase (Fig. 2, Fig. S9 and Dataset S7). Interestingly, Tudiarchaeales and Zaosheniarchaeales seem to use an alternative pathway for IPP biosynthesis, in which IPP is synthesized from isopentenyl phosphate via *ipk* rather than from 5-diphosphomevalonate via *mvaD*. All major lineages have phosphoglycerol geranylgeranyltransferase (GGGPS), geranylgeranylglycerol-phosphate geranylgeranyltransferase (DGGGPS) and cytidine diphosphate-diglyceride synthetase to convert glycerol-1-phosphate into geranylgeranylglycerol-phosphate (DGGGP). Norongarragalinales, WAL and Micrarchaeales also have *araM*, which encodes glycerol 1-phosphate dehydrogenase (G1PDH). Nearly complete pathways for membrane lipid biosynthesis in DPANN archaea were also found in previous studies [14,35]. However, Norongarragalinales have the complete pathway for archaeal membrane lipid backbone biosynthesis from acetyl-coenzyme A (CoA) to the final building block of archaeal membrane lipid—archaetidylinositol phosphate (archaeol), with most genomes having *pssA* genes encoding phosphatidylserine synthase that ultimately synthesizes archaeol. Although complete lipid biosynthetic pathways were previously found in Undinarchaeota, many other metabolic pathways, such as amino-acid biosynthesis and glycolysis, were missing or incomplete [15]. Furthermore, Norongarragalinales, Zaosheniarchaeales and Micrarchaeales also have a complete bacterial methylerythritol phosphate (MEP) pathway for IPP biosynthesis, which was formerly only detected in Woesearchaeales within Nanoarchaeota [36]. A previous study has shown that, when archaeal geranylgeranyl diphosphate and DGGGP synthase were expressed in *Escherichia coli*, it formed a heterochiral membrane with glycerol-3-phosphate (G3P)–bacterial lipids and G1P–archaeal lipids [37]. However, considering that only a few Micrarchaeales genomes encode *glpA* to synthesize G3P, it remains uncertain whether they could form the heterochiral membrane.

Additionally, although many DPANN have minimal amino-acid and cofactor biosynthetic capacity [36], some Micrarchaeota exhibit extraordinary capabilities in these aspects (Fig. 2 and Figs S10 and S11). For example, Norongarragalinales, WAL and Anstonellales are capable of the *de novo* biosynthesis of nearly all amino acids, except for lysine in Norongarragalinales, methionine in Norongarragalinales and Anstonellales, and tyrosine, tryptophan and phenylalanine in WAL. As for cofactors, complete or near-complete biosynthetic pathways for thiamine monophosphate/pyrophosphate, folate, tetrahydrofolate, flavin mononucleotide, flavin adenine dinucleotide, menaquinone, heme, nicotinamide adenine dinucleotide, nicotinamide adenine dinucleotide phosphate and CoA were also detected in some Micrarchaeota orders.

To simplify communication, we grouped the collection of genes associated with glycolysis, the TCA cycle, *de novo* nucleotide biosynthesis, *de novo* amino-acid biosynthesis and *de novo* cofactor biosynthesis as ‘putative free-living associated genes (pFLAGs)’. pFLAGs represent a set of functional genes that are ubiquitously present in free-living archaea and bacteria, but are often missing in symbiotic microorganisms such as DPANN archaea and CPR bacteria [3]. We then investigated the frequencies of pFLAGs across all major Micrarchaeota lineages and other archaea (Fig. 3 and Dataset S8). The comprehensive description of the distribution of pFLAGs on all DPANN archaea and other archaea consolidated the separation of their free-living capacities. Norongarragalinales, WAL and Anstonellales frequently encoded pFLAGs, similarly to other archaea, including members of free-living Altiarchaeota and putatively free-living Iainarchaeota [13,17,30]. By contrast, Tudiarchaeales, Zaosheniarchaeales, Redboyarchaeum and the yet-to-be-described phylum B1Sed10-29 generally resemble Huberarchaeota by lacking pFLAGs. The high completeness of pFLAG-associated pathways in Norongarragalinales was evident not only at the order level, but also when individual genomes were considered (see Dataset S8). For instance, the genome JZ-4_201803_bins_88 exhibited nearly complete *de novo* biosynthetic pathways for cell envelopes and nucleotides. Furthermore, NC_groundwater_1324_Ag_S-0.2um_56_85 and NC_groundwater_395_Ag_B-0.1um_58_13 encoded nearly complete *de novo* amino-acid biosynthesis pathways. Although these pathways were not always complete, they were comparable to those pathways found in the genomes of Altiarchaeota and other free-living archaea in terms of completeness. Unexpectedly, these pathways were not consistently complete within individual genomes of most other free-living archaeal lineages, except for Halobacteriota.

### Evolutionary mechanisms driving the acquisition of pFLAGs in Micrarchaeota

**Figure 4.**
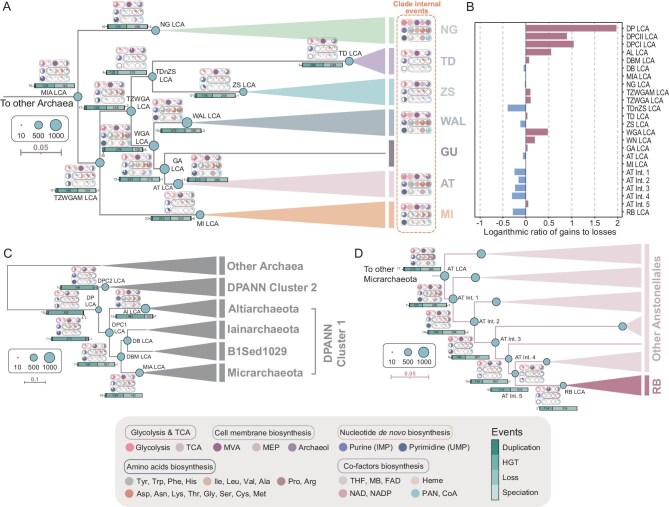
Reconstruction of evolutionary history of Micrarchaeota. (A, B, D) Schematic phylogenetic trees based on the rate-soothing DPANN phylogeny reconstructed from 494 of 658 genomes used for phylogenomic analyses with completeness of ≥90% and contamination of <5%. The size of the circle in each node indicates the proteome size of the node. Mapping of evolutionary event occurrences can be found in Dataset S10. LCA, the last common ancestor; MIA, Micrarchaeota; NG, Norongarragalinales; TD, Tudiarchaeales; ZS, Zaosheniarchaeales; WN, Wunengiarchaeales-associated lineages (WAL); AT, Anstonellales; MI, Micrarchaeales; TZWGAM, Tudiarchaeales, Zaosheniarchaeales, WAL, Gugararchaeales, Anstonellales and Micrarchaeales; TZWGA, Tudiarchaeales, Zaosheniarchaeales, WAL, Gugararchaeales and Anstonellales; WGA, WAL, Gugararchaeales and Anstonellales; TDnZS, Tudiarchaeales and Zaosheniarchaeales; DPC2, DPANN Cluster 2; DPC1, DPANN Cluster 1; DPANN, DPANN; AL, Altiarchaeota; IA, Iainarchaeota; IBM, Iainarchaeota, B1Sed-1029 and Micrarchaeota; IB, Iainarchaeota and B1Sed-1029; Int., internal node; RB, Redboyarchaeum. The pie charts of each node show the completeness of pFLAG-associated pathways on the corresponding node, including *de novo* nucleotide biosynthesis, glycolysis, *de novo* cell membrane biosynthesis, *de novo* amino-acid biosynthesis and *de novo* cofactor biosynthesis. The bar under the pie chart on each node represents the number of duplications, transfers, losses and origination events. (A) Trimmed tree illustrating the evolutionary history of Micrarchaeota. ‘Clade internal events’ indicates the sum of events regarding the presence of protein families after the diversification of the associated ancestral node. (B) Logarithmic ratio of gene gains to losses of each ancestral node. (C) Evolutionary history regarding ancestral nodes of Micrarchaeota and other archaea. (D) Evolutionary history regarding ancestral nodes of Redboyarchaeum within Anstonellales.

We have uncovered substantial metabolic capacity and, potentially, a capability for host independence in some major Micrarchaeota lineages. However, as others exhibit clear signs of reductive evolution, we sought to determine whether these core functions were ancestral or derived. To do so, we traced the gene-content evolution in Micrarchaeota by using the probabilistic gene-tree-aware reconciliation algorithm Amalgamated likelihood estimation (ALE) [38,39] with a particular focus on the evolutionary history of pFLAGs (Fig. 4). We explored a variety of substitution models and marker gene sets to establish a reference species tree for these analyses (Fig. S12A–E and Dataset S9; see details in the Supporting information). We first applied site-heterogeneous substitution models in an analysis of 53 GTDB archaeal markers to ameliorate the effect of long branch attraction on deep evolutionary inferences. The resulting phylogeny robustly divided DPANN archaea into two distinct lineages—Cluster 1 and Cluster 2—with strong bootstrap support (Fig. S12A, 100%), as suggested previously [15]. In this tree, the Altiarchaeota—which possess larger genomes and metabolic repertoires than most other DPANNs—branch with DPANN Cluster 1, with high bootstrap support (100%). While this position has been recovered in previous phylogenetic analyses [3,4,15,32], some other studies have instead recovered Altiarchaeota as the deepest-branching lineage within DPANN (i.e. sister to all other DPANN lineages) [2,13,14]. As resolving the placement of Altiarchaeota may have an effect on the scenarios of genome expansion and contraction within DPANN, we sought to investigate its phylogenetic position further. In our focal dataset, an approximately unbiased test rejected the basal placement of Altiarchaeota (*P* = 3.32e-54, Fig. S12B and Table S1) and analyses of three additional marker sets (14 ribosomal proteins (RPs) [3,11,32,40], 45 arCOGs and 49 RPs [41,42]) also provided no support for this position (Fig. S12C–E; see the Supporting information for further details). Taken together, our analyses support the view that Altiarchaeota branch at the base of Cluster 1, rather than forming an out-group to all other DPANNs.

We subsequently mapped the evolutionary history of 8866 arCOG and 39 KO families, each containing at least four sequences, onto the reference species tree by using gene tree–species tree reconciliation via the model implemented in ALE (Datasets S10 and S11) [38,39]. A total of 95 634 events (gene-family originations, duplications, transfers and losses) were identified since the last common ancestor of the Micrarchaeota (MIA LCA). Among these events, 1985 (2.08%) were inferred as duplications, 41 224 (42.99%) as HGTs, 52 277 (54.77%) as gene losses and 148 (0.16%) as originations (Fig. S13 and Dataset S12). The evolution of Micrarchaeota was thus dominated by HGT and gene loss, with occasional gene duplications. The last common ancestor of WAL, Gugararchaeales and Anstonellales (WGA LCA), the last common ancestor of WAL (WAL LCA) and the last common ancestor of Micrarchaeales (MI LCA) were among the hotspots of gene influxes contributed by HGT (top 50 nodes with highest HGT events; Fig. 4A, Fig. S13 and Dataset S13). The logarithmic ratios of gene gains over losses indicate that the proteome expansion, endowing WAL with core metabolism functions, was dominated by a major gene influx in the WAL LCA, similar to the last common ancestor of Altiarchaeota (AL LCA) (Fig. 4B and C, and Fig. S13). However, the gene gains of Norongarragalinales were counter-balanced by gene loss (Fig. 4A and B). Analyses focused on the pFLAGs showed that these genes were primarily acquired by HGT and this led to a possible transition to less host-dependent lifestyles in Norongarragalinales, WAL, Anstonellales and Micrarchaeales, especially for Norongarragalinales (Fig. 4A). The ‘completeness’ of the pFLAG-associated pathways was evaluated by calculating the percentage of present genes of each pathway at ancestral nodes. This revealed that the pFLAGs were gained through a process of continuous gene gain and loss rather than a short-lived burst of gene acquisition. The deficiency of the pFLAGs-associated pathways, especially nucleotides and cell membrane biosynthetic pathways at the last common ancestor of Norongarragalinales (NG LCA), the last common ancestor of Tudiarchaeales (TU LCA), the last common ancestor of Zaosheniarchaeales (ZS LCA), the last common ancestor of Wunengiarchaeales (WN LCA), the last common ancestor of Anstonellales (AT LCA) and the MI LCA suggest that the ancestors of each Micrarchaeota order were likely symbionts, albeit with somewhat more complete pFLAG-associated pathways in the ancestors of Norongarragalinales, WAL, Anstonellales and Micrarchaeales. The inference that the larger gene repertoires of these Micrarchaeota result from HGT-driven expansion from smaller ancestors was robust to several key analysis parameters, including the choice of substitution model used to infer the species and gene trees (single-matrix versus site-heterogeneous mixture models; Fig. S14A–D) and the position of Altiarchaeota in the species tree (Fig. S14E and Dataset S14).

By looking into detailed events that occurred at each node, we found that the glycolytic pathway was already >50% complete at the MIA LCA (Fig. 4A). Genes encoding 6-phosphofructokinase (*pfkA*), glyceraldehyde 3-phosphate dehydrogenase (*gapA*), phosphoglycerate kinase (*pgk*), 2,3-bisphosphoglycerate-independent phosphoglycerate mutase (*apgM*), enolase (*eno*) and ADP-dependent *pfkC* were likely to be present at the MIA LCA (Fig. S15). *ApgM* and *eno* may additionally have been present at the last common ancestor of DPANN (DPANN LCA), albeit with a complex evolutionary history. Although not passing the detection threshold by ALE, *PfkC* of Norongarragalinales was shown by phylogenetic inference to have been horizontally transferred from Nanohaloarchaeota (genus *Nanosalinia*) and possibly originated from Halobacteriota (class Halobacteria), while sequences of WAL were derived from Methanosarcinia or Nitrososphaeria (Fig. S16). The adenosine triphosphate (ATP)-dependent glucokinase (*Glk*) genes of Anstonellales were transferred from Nanoarchaeota and putatively originated from Thermoplasmatota (Fig. S17). The sequences of Norongarragalinales and Micrarchaeales were likely to be transferred from Thermoplasmatota (Thermoplasmata) and Halobacteriota (*Halobacteria*). Others, including genes encoding glucose-6-phosphate isomerase (*pgi1*), glucose/mannose-6-phosphate isomerase (*pgi-pmi*), fructose-bisphosphate aldolase, class II (*fbaA*), triosephosphate isomerase (*tpiA*) and pyruvate kinase (*pyk*) were shown to be horizontally acquired at later stages of evolution. This indicates that the assembly of the glycolytic pathway was only complete during the diversification of Norongarragalinales, WAL, Anstonellales and Micrarchaeales, and thus is not an ancestral feature of any of these lineages. As for the TCA cycle, only one gene *fumC* encoding class II fumarate hydratase was detected to be present in the MIA LCA (Fig. S18). The full set of genes for Norongarragalinales were completely gained via HGT after the diversification of the NG LCA. Micrarchaeales, however, experienced an assembly process from the last common ancestor of Tudiarchaeales, Zaosheniarchaeales, WAL, Gugararchaeales, Anstonellales and Micrarchaeales, the MI LCA and after the diversification of the MI LCA by HGT.

Genes associated with the MVA pathway, including those encoding acetyl-CoA C-acetyltransferase (*ACAT*), hydroxymethyl-glutaryl–CoA (HMG–CoA) synthase (HMGCS), HMG–CoA reductase (HMGCR), mevalonate kinase (MVK), phosphomevalonate kinase (PMVK), mevalonate pyrophosphate decarboxylase (MVD) and isopentenyl diphosphate Delta-isomerase (*idi*) were shown to present be in the MIA LCA (Fig. S19). Except for HMGCR and PMVK, most of these genes were vertically descended from the DPANN LCA. Further phylogenetic analysis suggested that most Micrarchaeota *mvk* genes form a monophyletic lineage adjacent to Thermoproteota and the internal topology was similar to the phylogenomic tree, suggesting the conservation and vertical evolution of this branch, and may represent a vertically acquired feature (Fig. S20). Most genes of the MEP pathway except *dxs* encoding 1-deoxy-D-xylulose-5-phosphate synthase were horizontally acquired by Norongarragalinales, Zaosheniarchaeales and Micrarchaeales after the diversification of these orders. Although the hallmark gene *araM* encoding G1PDH of phospholipid biosynthesis was detected in the DPANN LCA, it was lost in the last common ancestor of Iainarchaeota, B1Sed10-29 and Micrarchaeota (IBM LCA) and then regained in the descendants of the NG LCA and WAL LCA (Fig. S19). The regained G1PDH of Norongarragalinales and Aenigmarchaeota were placed within a large branch consisting of Halobacteria and Methanobacteriota, from whom these genes may have been acquired horizontally, but the WAL G1PDH was derived from Thermoproteota (Thermoproteia) (Fig. S21). Another hallmark gene of lipid biosynthesis, *PssA*, appears to have vertically descended from the DPANN LCA to modern Norongarragalinales, except for some Anstonellales sequences, which may have a different origin, along with G1PDH in Aenigmarchaeota and Undinarchaeota (Fig. S22). Taken together, these results demonstrate that the MIA LCA could not synthesize an archaeal membrane and the complete biosynthetic pathway in Norongarragalinales was a mix of vertical transmissions and HGTs.

Similarly, *de novo* nucleotide biosynthetic pathways were already >50% complete at the MIA LCA (Fig. 4A and Dataset S8). *PyrF* and other genes of the pyrimidine synthesis were present in the DPANN LCA and vertically transmitted to its descendants, including Micrarchaeota (Fig. S23). Interestingly, multiple origins of *PyrF* were observed within Micrarchaeota, with several genes from Anstonellales and Norongarragalinales, possibly derived from Thermoproteota. No explicit HGTs were observed for several lineages, including WAL and Norongarragalinales (Fig. S24). In contrast, many genes in the purine biosynthesis pathway, including *purF*, the phosphoribosylamine–glycine ligase (*purD*), phosphoribosylglycinamide formyltransferase 1 (*purN*), the phosphoribosylformylglycinamidine synthase subunit PurL (*purL*), the phosphoribosylformylglycinamidine synthase subunit PurS (*purS*), phosphoribosylformylglycinamidine cyclo-ligase (*purM*), phosphoribosylaminoimidazole carboxylase/phosphoribosylaminoimidazolesuccinocarboxamide synthase and adenylosuccinate lyase (*purB*), were present in the DPANN LCA; many genes were shown to be lost in the IBM LCA (Fig. S23). Considering the phylogeny of AICAR transformylase/IMP cyclohydrolase, it seems likely that frequent HGTs occurred because some DPANN PurH sequences are mixed with Halobacteriota and Thermoplasmatota, while other genes of WAL and Anstonellales appeared to have evolved vertically (Fig. S25). Altogether, we inferred that the ability to *de novo* synthesize nucleotides was not an ancestral feature of Norongarragalinales, WAL and Anstonellales, but was rather later acquired horizontally and gradually at different stages of evolution.

By looking into the evolution of pathways associated with the *de novo* biosynthesis of amino acids and cofactors, we observed that they were highly incomplete in the MIA LCA (Fig. 4A). Similarly to nucleotide biosynthesis pathways, the complete or near-complete *de novo* amino-acid biosynthetic pathways found in the contemporary Norongarragalinales, WAL and Anstonellales MAGs were mostly acquired via HGT separately after the diversification of each lineage (Figs S26–S30). Slightly more than a few of the genes for *de novo* amino-acid and cofactor biosynthesis were present in the MIA LCA, including 18 out of 75 for amino-acid synthesis and 8 out of 45 for cofactor biosynthetic pathways. Interestingly, the genes present in the last common ancestors of each Micrarchaeota major lineage were associated with the final step of the biosynthesis of several amino acids. These include key genes for tyrosine and phenylalanine synthesis (*aspB/hisC*) at the NG LCA and WAL LCA; tryptophan synthesis (*trpB*) for the WAL LCA and AT LCA; histidine synthesis (*hisD*) for the WAL LCA; leucine, isoleucine, valine synthesis (*ilvE*) and alanine synthesis (*alaA/ala*) for WAL LCA, AT LCA and MI LCA; aspartate and asparagine synthesis (*aspB*/*asnAB*) for the WAL LCA; lysine synthesis (*lysA*) for the NG LCA, WAL LCA and AT LCA; threonine synthesis (*thrC*) for WAL LCA, AT LCA and MI LCA; and glycine (*kbl*) and serine (*serA*) synthesis for the AT LCA and WAL LCA.

### Reductive evolution of Redboyarchaeum

As mentioned above, Redboyarchaeum genomes were highly compact and ultra-streamlined with very limited inferred metabolic capabilities (Fig. 2, Figs S3 and S14, and Dataset S1). The high proportions of genes associated with information storage and processing indicated that the genome reduction may have been induced by random losses of genes associated with nonessential functions (Fig. S31). The evolutionary history reconstruction reveals that extensive gene losses occurred in the last common ancestor of Redboyarchaeum (RB LCA) with a tremendous 224 losses compared with 2 duplications and 114 HGTs (Fig. 4D). Additionally, the trend of gene losses over gene gains continued after the AT LCA. Despite having ‘less streamlining’, the reduced genome sizes of Zaosheniarchaeales were also putatively driven by ongoing gene losses over gene gain from the last common ancestor of Tudiarchaeales and Zaosheniarchaeales to the ZS LCA.

## DISCUSSION

The co-isolation of *Ca. Nanoarchaeum equitans* with its archaeal host, along with the discovery of ultra-small ARMAN archaea including Micrarchaeota, introduced the scientific community to life with highly reduced genomes inhabiting extreme environments [6–8,18]. We recovered 163 Micrarchaeota MAGs classified into three known orders (Norongarragalinales, Anstonellales and Micrarchaeales) and five orders described (Tudiarchaeales, Zaosheniarchaeales, Wunengiarchaeales, Bailongiarchaeales and Wujingiarchaeales) in this study. The evolution of Tudiarchaeales, Zaosheniarchaeales and Redboyarchaeum was marked by extensive gene losses, resulting in metabolic deficiencies in these archaea. Consequently, they almost certainly rely on other microbes to support their growth. Such metabolic limitations are not exclusive to DPANN archaea, as the earlier discovery of *Panguiarchaeum symbiosum* provided a second example of gene loss and likely symbiosis within the archaea [43]. In addition to nutrient exchanges, frequent genetic interactions might also occur between symbionts and their hosts due to their direct cell-to-cell contact [44,45]. By deducing the origins of HGTs, we conjectured that Thermoproteota may be a potential host of Zaosheniarchaeales (Fig. S32). Intriguingly, a considerable number of HGTs were detected between Tudiarchaeales and Anstonellales, implying the possibility of DPANN archaea serving as hosts. Other symbiotic relationships between symbiont and host ‘pairs’ can also be inferred, such as Nanohaloarchaeota and Halobacteriota, Huberarchaeota and Altiarchaeota, and Micrarchaeota and Thermoplasmatota, consistently with previous studies [14,22–25,27,44,45]. Further analyses revealed that HGT was the major driver of the genome expansion of Micrarchaeota lineages, consistently with prior notions that HGT plays a crucial role in shaping genetic diversity and the evolutionary trajectory of prokaryotes, especially in extreme environments [46–48]. Additionally, for Micrarchaeales, we detected considerable HGTs accompanied by a large number of gene losses. This may be attributed to their adaptation and preferential distribution in acidic environments, as documented in previous studies [7,8,18,20,49]. Such processes may lead to the shuffling and turnover of their proteomes, further driving their divergence from other lineages based on their function profiling (Fig. S4).

In contrast, comparative genomic analyses revealed that Norongarragalinales, WAL, Anstonellales and Micrarchaeales possess pathways for central carbon metabolism, energy conservation and biosynthetic capacities through the HGT of pFLAGs. In particular, HGT-acquired pFLAGs might confer upon Norongarragalinales a reduced dependence on hosts or even the capacity to be free-living, as also suggested for Altiarchaeota, Iainarchaeota, Woesearchaeota and Undinarchaeota [13–15,35]. Notably, previous studies suggested that Altiarchaeota are a free-living autotrophic DPANN lineage that could use a modified WLP for carbon fixation [29] and may even serve as a host for Huberarchaeota [13,14]. By reconstructing the evolutionary history of DPANN, we found that AL LCA likely exhibited a symbiotic lifestyle characterized by incomplete catabolic and/or anabolic pathways. Given the considerable number of HGT events following the diversification of the AL LCA (Fig. S11), we reasoned that the free-living capacity, indicated by the presence of pFLAGs (Fig. 3), appeared to be an acquired feature driven by HGT. Interestingly, such evolutionary trajectories may have also occurred in parallel in other DPANN archaea lineages. For example, many pathways in Iainarchaeota, such as the ATP production and biosynthesis of key metabolites, were reported to have ancient origins from bacteria [17,28]. Notably, biosynthetic pathways for certain amino acids were even found to be entirely acquired via HGT.

Interestingly, the acquisitions of pFLAGs in different pathways exhibit distinct evolutionary histories. Similarly to the methanogen-to-halophile transition of Hikarchaeota [50], this process entailed gradual influxes of associated gene families after Norongarragalinales, WAL, Anstonellales and Micrarchaeales diversified, rather than the acquisition of entire catabolic or anabolic pathways at a single ancestral node (Fig. 4A and B). Pathways including glycolysis, archaeal membrane and *de novo* nucleotide biosynthesis were ≥50% complete before the MIA LCA. However, pFLAGs involving amino-acid and cofactor biosynthesis were mostly acquired after the MIA LCA. For Norongarragalinales and Micrarchaeales, there was not >50% completeness until the diversification of the NG LCA and MI LCA. Genes involved in the membrane biosynthesis pathway appear to be more conservative [51]. This could be exemplified by genes such as *ACAT*, HMGCS, MVK, MVD, *idi*, GGPS DGGGPS and *pssA*, which are mostly inherited from the DPANN LCA (Fig. S19). In contrast, genes involved in amino-acid and nucleotide biosynthesis were prone to undergoing HGTs [52,53]. Intriguingly, those ancestors possess several genes related to the last steps of amino-acid biosynthesis, which might have acted as the backbones to boost the later gradual evolution of relevant genes and eventually led to the formation of the complete pathways. This implies the potential for amino-acid biosynthesis in the presence of certain substrates or intermediates, which may have been provided by their symbiotic partners or come from elsewhere. The loss of amino-acid and cofactor biosynthetic capacities was common in symbionts compared with free-living siblings, and amino-acid auxotrophy has also been suggested to be more prevalent in obligate parasitic bacteria [54,55]. Thus, the possession of amino-acid and cofactor biosynthetic pathways may further suggest the substantial independent growth potentials of these lineages. On top of that, cofactors participated in a variety of catalytic enzyme activities, such as carbohydrate and amino-acid metabolism, redox reactions and metabolic activation, and were thus vital for the independent survival of microorganisms. The ultra-strong cofactor biosynthetic capacities of Micrarchaeota are comparable to and even surpass those of certain eukaryotic organisms [56].

As for Tudiarchaeales, Zaosheniarchaeales, Redboyarchaeum and B1Sed10-29 phylum, they lack pFLAG-associated pathways, resembling obligate symbiont Huberarchaeota [14] (Fig. 3 and Dataset S8). These extended the notion that, besides members of DPANN Cluster 2, lineages among DPANN Cluster 1 may also be characterized by reductive evolution and inferior metabolic capacity [15]. Among them, Zaosheniarchaeales and Redboyarchaeum represent two Micrarchaeota lineages with reduced genome sizes and ultra-low GC content that may tentatively be a result of endosymbiosis (Fig. S3A and D) [57]. The ‘non-streamlining’ of Zaosheniarchaeales versus Redboyarchaeum could indicate an earlier stage of genome erosion (Fig. S3E and F) [57]. Further evolutionary analyses also showed that the inferior metabolic capacity can be traced back to their respective last common ancestors, which were shown to experience reductive evolution since the MIA LCA (Fig. 4C). As shown in our results, the genome sizes of Redboyarchaeum and associated lineages were negatively correlated with their OGTs (Fig. S2C and D), representing a hyperthermophilic-specific lineage similar to that of the previously discovered Haiyanarchaeum [26]. A look into the amino-acid frequencies of these lineages (Fig. S33A and B) reveals that the proteomes of Redboyarchaeum were enriched with Asp (D), Arg (R), Leu (L), Ile (I) and Tyr (Y), with the majority among the ‘IVYWREL’, which is significantly correlated with OGTs when overrepresented in proteomes [58]. Specifically, Asp was also among the charged amino acids of ‘charged versus polar (non-charged) amino acids (CvP-bias)’, which also showed a strong correlation with OGTs [59]. Moreover, albeit not detected in genomes of this study, *rgy* genes that were associated with hyperthermal adaptation were also solely detected in genomes of Redboyarchaeum (Fig. 3 and Dataset S7). The adaptation to high-temperature environments of Anstonellales may also be associated with the utilization of a branched ED glycolysis pathway. This pathway has previously been described in members of the hyperthermophilic archaea *Sulfolobus solfataricus* and *Thermoplasma acidophilum*, which may grant them evolutionary advantages by diminishing the carbon loss induced by thermolabile intermediates in hyperthermal environments [60,61]. Combined with the continuous gene losses since the AT LCA (Fig. 4B and D), the genome streamlining observed in hyperthermophilic Redboyarchaeum was tentatively attributed to adaptation to high-temperature environments.

Taken together, we propose an evolutionary scenario in which some modern DPANN archaea evolved from symbiotic thermophilic ancestors through HGT-driven genome expansion, emerging with metabolic capacity that may endow them with the potential for host independence and free-living growth. We also conjecture that temperature plays a substantial role in shaping the evolution of these lineages. Further, the inference that the most deep-rooted genomes of all major Micrarchaeota lineages except Gugararchaeales (with only one representative genome) derive from high-temperature environments may suggest the thermophilic origin of Micrarchaeota. Our study reveals their origin in extreme environments and evolutionary diversification into various extreme and nonextreme habitats, hence depicting the comprehensive panorama of the evolutionary history of this phylum.

## MATERIALS AND METHODS

### Metagenomic assembly and genome binding

Raw sequencing data were preprocessed as previously described [48] by using a customized Perl script (quality_control.pl, https://github.com/hzhengsh/qualityControl). All quality reads of each dataset were *de novo* assembled by using SPAdes (v3.9.0) [62], with the parameters: -k 21,33,55,77,99 127 –meta. In each assembly, scaffolds with a length of <2500 bp were removed. BBMap (v38.92) (http://sourceforge.net/projects/bbmap/) was used to calculate the coverage information by mapping clean reads to corresponding assembled scaffolds with parameters: k = 15 minid = 0.97 build = 1. Three pieces of software—CONCOCT (v.1.1.0) [63], Maxbin2 (v.2.2.7) [64] and MetaBAT (version 2.12.1) [65]—were used for automated binning to generate candidate bins for each sample. The best bins were selected by using DASTool (v.1.1.3) [66]. To further improve the quality of the genomes, all bins were mapped by using the quality reads BBMap and reassembled with SPAdes via the following parameters: -k 21,33,55,77,99 127 –careful. CheckM (v1.1.3) was used to estimate the contaminations and strain heterogeneity of reassembled bins [33], while the metric completeness was calculated with 48 previously described single-copy genes [32]. In addition, CheckM2 was implemented to further assess the genome quality of the DPANN archaea [67]. The preliminary taxonomic assignment of each MAG was acquired via GTDB-tk (v1.6.0) [68,69]. Moreover, dRep (v3.2.2) [70] was used to dereplicate all 163 genomes in this study at 99% average nucleotide identity (strain level) and some MAGs were manually selected to maximize the characterized phylogenetic diversity (noted in Dataset S1).

### Functional annotation of genomes

Putative protein-coding sequences (CDS) were identified using Prodigal (v2.6.3) under the ‘-p single’ model. Predicted CDSs were compared against the KEGG database [71] with KofamKOALA [72] and arCOG [73] databases using DIAMOND (v2.0.11.149) [74] with a cutoff of 1e-5. Hydrogenases were annotated via the local version of HydDB [75]. Carbonhydrate-active enzymes (CAZy) [76] were annotated using dbcan2 (dbscan) with the parameters: –hmm_eval 1e-5 –dia_eval 1e-5 [77]. Annotation results were kept if CAZys were identified by at least two of the three tools (HMMER, DIAMOND and eCAMI [78]). CDSs were also searched for PFAMs and TIGRFAMs using InterProScan (v5.36–75.0) [79] package with the following parameters: -dp -f tsv -goterms -pa. Transporters were identified by Transporter Classification Database (TCDB) [80] using DIAMOND with E-value < 1e-5. CRISPR-Cas systems were detected with CRISPRCasFinder (v4.3.2) [81]. The rRNA-coding regions were determined by using barrnap (v0.9, https://github.com/tseemann/barrnap). tRNAscan-SE (v2.0.9) [82] was used to identify tRNAs of all MAGs.

### Phylogenetic and phylogenomic analysis

A total of 658 archaeal reference genomes were carefully selected from public databases for phylogenomic analyses based on previous studies [15,43] (Dataset S5). The GTDB 53 archaeal conserved markers were selected and concatenated to reconstruct phylogeny [5]. We were cautious in selecting suitable markers for reconstructing the species tree. These marker sequences were identified by using AMPHORA2 [83]. The individual markers were aligned by using MAFFT (v7.487) [84] with ‘linsi’ options by iterating 1000 times. Poorly aligned regions were trimmed via TrimAl (v1.4.rev22) [85] with the ‘-gappyout’ option (14 736 amino-acid positions were retained). Multiple sequence alignments were concatenated by using a self-made Perl script (concatenate_multiple_genes.pl, https://github.com/hzhengsh/phylogeny). The phylogenetic tree was constructed by using IQ-TREE (v1.6.11) [86] with the parameter: -alrt 1000 -nt AUTO. The best-fitting model of the phylogenomic tree is LG + F + R10.

Supporting methods regarding sampling, experiments, phylogenetic analyses, and comparative and evolutionary genomics are listed in the Supporting information.

## Supplementary Material

nwaf542_Supplemental_Files

## Data Availability

All datasets analysed or generated in this study, including all MAGs and reference genomes and all workflows for phylogeny, annotations and comparative/evolutionary genomic analyses, were deposited at Zenodo (10.5281/zenodo.11170968). MAGs generated in this study are also accessible at NCBI under the BioProject ID PRJNA544494 (the accession ID of each MAG can be found in Dataset S1).

## References

[1] Offre P, Spang A, Schleper C. Archaea in biogeochemical cycles. Annu Rev Microbiol 2013; 67: 437–57. 10.1146/annurev-micro-092412-155614 23808334

[2] Baker BJ, De Anda V, Seitz KW et al. Diversity, ecology and evolution of Archaea. Nat Microbiol 2020; 5: 887–900. 10.1038/s41564-020-0715-z 32367054

[3] Castelle CJ, Brown CT, Anantharaman K et al. Biosynthetic capacity, metabolic variety and unusual biology in the CPR and DPANN radiations. Nat Rev Micro 2018; 16: 629–45. 10.1038/s41579-018-0076-2 30181663

[4] Dombrowski N, Lee J-H, Williams TA et al. Genomic diversity, lifestyles and evolutionary origins of DPANN archaea. FEMS Microbiol Lett 2019; 366: fnz008. 10.1093/femsle/fnz008 30629179 PMC6349945

[5] Rinke C, Chuvochina M, Mussig AJ et al. A standardized archaeal taxonomy for the genome taxonomy database. Nat Microbiol 2021; 6: 946–59. 10.1038/s41564-021-00918-8 34155373

[6] Huber H, Hohn MJ, Rachel R et al. A new phylum of Archaea represented by a nanosized hyperthermophilic symbiont. Nature 2002; 417: 63–7. 10.1038/417063a 11986665

[7] Baker BJ, Tyson GW, Webb RI et al. Lineages of acidophilic archaea revealed by community genomic analysis. Science 2006; 314: 1933–5. 10.1126/science.1132690 17185602

[8] Baker BJ, Comolli LR, Dick GJ et al. Enigmatic, ultrasmall, uncultivated Archaea. Proc Natl Acad Sci USA 2010; 107: 8806–11. 10.1073/pnas.0914470107 20421484 PMC2889320

[9] Narasingarao P . De novo metagenomic assembly reveals abundant novel major lineage of archaea in hypersaline microbial communities. ISME J 2012; 6: 81–93. 10.1038/ismej.2011.78 21716304 PMC3246234

[10] Rinke C, Schwientek P, Sczyrba A et al. Insights into the phylogeny and coding potential of microbial dark matter. Nature 2013; 499: 431–7. 10.1038/nature12352 23851394

[11] Castelle CJ, Wrighton KC, Thomas BC et al. Genomic expansion of domain archaea highlights roles for organisms from new phyla in anaerobic carbon cycling. Curr Biol 2015; 25: 690–701. 10.1016/j.cub.2015.01.014 25702576

[12] Probst AJ, Castelle CJ, Singh A et al. Genomic resolution of a cold subsurface aquifer community provides metabolic insights for novel microbes adapted to high CO2 concentrations. Environ Microbiol 2017; 19: 459–74. 10.1111/1462-2920.13362 27112493

[13] Probst AJ, Ladd B, Jarett JK et al. Differential depth distribution of microbial function and putative symbionts through sediment-hosted aquifers in the deep terrestrial subsurface. Nat Microbiol 2018; 3: 328–36. 10.1038/s41564-017-0098-y 29379208 PMC6792436

[14] Schwank K, Bornemann TLV, Dombrowski N et al. An archaeal symbiont-host association from the deep terrestrial subsurface. ISME J 2019; 13: 2135–9. 10.1038/s41396-019-0421-0 31048756 PMC6776059

[15] Dombrowski N, Williams TA, Sun J et al. Undinarchaeota illuminate DPANN phylogeny and the impact of gene transfer on archaeal evolution. Nat Commun 2020; 11: 3939. 10.1038/s41467-020-17408-w 32770105 PMC7414124

[16] Parks DH, Rinke C, Chuvochina M et al. Recovery of nearly 8,000 metagenome-assembled genomes substantially expands the tree of life. Nat Microbiol 2017; 2: 1533–42. 10.1038/s41564-017-0012-7 28894102

[17] Youssef NH, Rinke C, Stepanauskas R et al. Insights into the metabolism, lifestyle and putative evolutionary history of the novel archaeal phylum ‘Diapherotrites’. ISME J 2015; 9: 447–60. 10.1038/ismej.2014.141 25083931 PMC4303637

[18] Chen L-X, Méndez-García C, Dombrowski N et al. Metabolic versatility of small archaea Micrarchaeota and Parvarchaeota. ISME J 2018; 12: 756–75. 10.1038/s41396-017-0002-z 29222443 PMC5864196

[19] Liu X, Li M, Castelle CJ et al. Insights into the ecology, evolution, and metabolism of the widespread Woesearchaeotal lineages. Microbiome 2018; 6: 102. 10.1186/s40168-018-0488-2 29884244 PMC5994134

[20] Shu W-S, Huang L-N. Microbial diversity in extreme environments. Nat Rev Micro 2022; 20: 219–35. 10.1038/s41579-021-00648-y 34754082

[21] Beam JP, Becraft ED, Brown JM et al. Ancestral absence of electron transport chains in patescibacteria and DPANN. Front Microbiol 2020; 11: 1848. 10.3389/fmicb.2020.01848 33013724 PMC7507113

[22] Hamm JN, Erdmann S, Eloe-Fadrosh EA et al. Unexpected host dependency of Antarctic Nanohaloarchaeota. Proc Natl Acad Sci USA 2019; 116: 14661–70. 10.1073/pnas.1905179116 31253704 PMC6642349

[23] Golyshina OV, Toshchakov SV, Makarova KS et al. ‘ARMAN’ archaea depend on association with euryarchaeal host in culture and in situ. Nat Commun 2017; 8: 60. 10.1038/s41467-017-00104-7 28680072 PMC5498576

[24] Krause S, Gfrerer S, von Kügelgen A et al. The importance of biofilm formation for cultivation of a Micrarchaeon and its interactions with its Thermoplasmatales host. Nat Commun 2022; 13: 1735. 10.1038/s41467-022-29263-y 35365607 PMC8975820

[25] Sakai HD, Nur N, Kato S et al. Insight into the symbiotic lifestyle of DPANN archaea revealed by cultivation and genome analyses. Proc Natl Acad Sci USA 2022; 119: e2115449119. 10.1073/pnas.2115449119 35022241 PMC8784108

[26] Rao Y-Z, Li Y-X, Li Z-W et al. Metagenomic discovery of ‘Candidatus Parvarchaeales’-related lineages sheds light on adaptation and diversification from neutral-thermal to acidic-mesothermal environments. mSystems 2023; 8: e0125222. 36943058 10.1128/msystems.01252-22 PMC10134863

[27] La Cono V, Messina E, Rohde M et al. Symbiosis between nanohaloarchaeon and haloarchaeon is based on utilization of different polysaccharides. Proc Natl Acad Sci USA 2020; 117: 20223–34. 10.1073/pnas.2007232117 32759215 PMC7443923

[28] Probst AJ, Banfield JF. Homologous recombination and transposon propagation shape the population structure of an organism from the deep subsurface with minimal Metabolism. Genome Biol Evol 2018; 10: 5. 10.1093/gbe/evy067 PMC5905446 29672704

[29] Probst AJ, Weinmaier T, Raymann K et al. Biology of a widespread uncultivated archaeon that contributes to carbon fixation in the subsurface. Nat Commun 2014;5: 5497. 10.1038/ncomms6497 25425419

[30] Esser SP, Rahlff J, Zhao W et al. A predicted CRISPR-mediated symbiosis between uncultivated archaea. Nat Microbiol 2023; 8: 1619–33. 37500801 10.1038/s41564-023-01439-2

[31] Vázquez-Campos X, Kinsela AS, Bligh MW et al. Genomic insights into the archaea inhabiting an Australian radioactive legacy site. Front Microbiol 2021; 12: 732575. 34737728 10.3389/fmicb.2021.732575 PMC8561730

[32] He C, Keren R, Whittaker ML et al. Genome-resolved metagenomics reveals site-specific diversity of episymbiotic CPR bacteria and DPANN archaea in groundwater ecosystems. Nat Microbiol 2021; 6: 354–65. 10.1038/s41564-020-00840-5 33495623 PMC7906910

[33] Parks DH, Imelfort M, Skennerton CT et al. CheckM: assessing the quality of microbial genomes recovered from isolates, single cells, and metagenomes. Genome Res 2015; 25: 1043–55. 10.1101/gr.186072.114 25977477 PMC4484387

[34] Hedlund BP, Chuvochina M, Hugenholtz P et al. SeqCode: a nomenclatural code for prokaryotes described from sequence data. Nat Microbiol 2022; 7: 1702–8. 36123442 10.1038/s41564-022-01214-9 PMC9519449

[35] Huang W-C, Liu Y, Zhang X et al. Comparative genomic analysis reveals metabolic flexibility of Woesearchaeota. Nat Commun 2021; 12: 5281. 10.1038/s41467-021-25565-9 34489402 PMC8421398

[36] Castelle CJ, Banfield JF. Major new microbial groups expand diversity and alter our understanding of the tree of life. Cell 2018; 172: 1181–97. 10.1016/j.cell.2018.02.016 29522741

[37] Caforio A, Siliakus MF, Exterkate M et al. Converting Escherichia coli into an archaebacterium with a hybrid heterochiral membrane. Proc Natl Acad Sci USA 2018; 115: 3704–9. 10.1073/pnas.1721604115 29555770 PMC5889666

[38] Szöllősi GJ, Rosikiewicz W, Boussau B et al. Efficient exploration of the space of reconciled gene trees. Syst Biol 2013; 62: 901–12. 23925510 10.1093/sysbio/syt054 PMC3797637

[39] Groussin M, Hobbs JK, Szöllősi GJ et al. Toward more accurate ancestral protein genotype–phenotype reconstructions with the use of species tree-aware gene trees. Mol Biol Evol 2015; 32: 13–22. 10.1093/molbev/msu305 25371435 PMC4271536

[40] Hug LA, Baker BJ, Anantharaman K et al. A new view of the tree of life. Nat Microbiol 2016; 1: 16048. 10.1038/nmicrobiol.2016.48 27572647

[41] Eme L, Tamarit D, Caceres EF et al. Inference and reconstruction of the heimdallarchaeial ancestry of eukaryotes. Nature 2023; 618: 992–9. 37316666 10.1038/s41586-023-06186-2 PMC10307638

[42] Valentin-Alvarado LE, Appler KE, De Anda V et al. Asgard archaea modulate potential methanogenesis substrates in wetland soil. Nat Commun 2024; 15: 6384. 10.1038/s41467-024-49872-z 39085194 PMC11291895

[43] Qu Y-N, Rao Y-Z, Qi Y-L et al. Panguiarchaeum symbiosum, a potential hyperthermophilic symbiont in the TACK superphylum. Cell Rep 2023; 42: 112158. 10.1016/j.celrep.2023.112158 36827180

[44] Hotopp JCD, Clark ME, Oliveira DCSG et al. Widespread lateral gene transfer from intracellular bacteria to multicellular eukaryotes. Science 2007; 317: 1753–6. 10.1126/science.1142490 17761848

[45] Jarett JK, Nayfach S, Podar M et al. Single-cell genomics of co-sorted Nanoarchaeota suggests novel putative host associations and diversification of proteins involved in symbiosis. Microbiome 2018; 6: 161. 10.1186/s40168-018-0539-8 30223889 PMC6142677

[46] Zhaxybayeva O, Swithers KS, Lapierre P et al. On the chimeric nature, thermophilic origin, and phylogenetic placement of the Thermotogales. Proc Natl Acad Sci USA 2009; 106: 5865–70. 10.1073/pnas.0901260106 19307556 PMC2667022

[47] Nelson-Sathi S, Dagan T, Landan G et al. Acquisition of 1,000 eubacterial genes physiologically transformed a methanogen at the origin of Haloarchaea. Proc Natl Acad Sci USA 2012; 109: 20537–42. 10.1073/pnas.1209119109 23184964 PMC3528564

[48] Hua Z-S, Qu Y-N, Zhu Q et al. Genomic inference of the metabolism and evolution of the archaeal phylum Aigarchaeota. Nat Commun 2018; 9: 2832. 10.1038/s41467-018-05284-4 30026532 PMC6053391

[49] Hua Z-S, Han Y-J, Chen L-X et al. Ecological roles of dominant and rare prokaryotes in acid mine drainage revealed by metagenomics and metatranscriptomics. ISME J 2015; 9: 1280–94. 10.1038/ismej.2014.212 25361395 PMC4438317

[50] Martijn J, Schön ME, Lind AE et al. Hikarchaeia demonstrate an intermediate stage in the methanogen-to-halophile transition. Nat Commun 2020; 11: 5490. 10.1038/s41467-020-19200-2 33127909 PMC7599335

[51] Koonin EV . Comparative genomics, minimal gene-sets and the last universal common ancestor. Nat Rev Micro 2003; 1: 127–36. 10.1038/nrmicro751 15035042

[52] Husnik F, Nikoh N, Koga R et al. Horizontal gene transfer from diverse bacteria to an insect genome enables a tripartite nested mealybug symbiosis. Cell 2013; 153: 1567–78. 10.1016/j.cell.2013.05.040 23791183

[53] Husnik F, McCutcheon JP. Functional horizontal gene transfer from bacteria to eukaryotes. Nat Rev Micro 2018; 16: 67–79. 10.1038/nrmicro.2017.137 29176581

[54] Ramoneda J, Jensen TBN, Price MN et al. Taxonomic and environmental distribution of bacterial amino acid auxotrophies. Nat Commun 2023; 14: 7608. 10.1038/s41467-023-43435-4 37993466 PMC10665431

[55] Boscaro V, Felletti M, Vannini C et al. Polynucleobacter necessarius, a model for genome reduction in both free-living and symbiotic bacteria. Proc Natl Acad Sci USA 2013; 110: 18590–5. 10.1073/pnas.1316687110 24167248 PMC3831957

[56] Krishnan A, Kloehn J, Lunghi M et al. Vitamin and cofactor acquisition in apicomplexans: synthesis versus salvage. J Biol Chem 2020; 295: 701–14. 10.1016/S0021-9258(17)49928-5 31767680 PMC6970920

[57] McCutcheon JP, Moran NA. Extreme genome reduction in symbiotic bacteria. Nat Rev Micro 2012; 10: 13–26. 10.1038/nrmicro2670 22064560

[58] Zeldovich KB, Berezovsky IN, Shakhnovich EI. Protein and DNA sequence determinants of thermophilic adaptation. PLoS Comput Biol 2007; 3: e5. 10.1371/journal.pcbi.0030005 17222055 PMC1769408

[59] Suhre K, Claverie J-M. Genomic correlates of hyperthermostability, an update *. J Biol Chem 2003; 278: 17198–202. 10.1074/jbc.M301327200 12600994

[60] Figueiredo AS, Kouril T, Esser D et al. Systems biology of the modified branched Entner-Doudoroff pathway in sulfolobus solfataricus. PLoS One 2017; 12: e0180331. 10.1371/journal.pone.0180331 28692669 PMC5503249

[61] Ahmed H, Ettema TJG, Tjaden B et al. The semi-phosphorylative Entner–Doudoroff pathway in hyperthermophilic archaea: a re-evaluation. Biochem J 2005; 390: 529–40. 10.1042/BJ20041711 15869466 PMC1198933

[62] Bankevich A, Nurk S, Antipov D et al. SPAdes: a new genome assembly algorithm and its applications to single-cell sequencing. J Comput Biol 2012; 19: 455–77. 10.1089/cmb.2012.0021 22506599 PMC3342519

[63] Alneberg J, Bjarnason BS, de Bruijn I et al. Binning metagenomic contigs by coverage and composition. Nat Methods 2014; 11: 1144–6. 10.1038/nmeth.3103 25218180

[64] Wu Y-W, Simmons BA, Singer SW. MaxBin 2.0: an automated binning algorithm to recover genomes from multiple metagenomic datasets. Bioinformatics 2016; 32: 605–7. 10.1093/bioinformatics/btv638 26515820

[65] Kang DD, Froula J, Egan R et al. MetaBAT: metagenome binning based on abundance and Tetranucleotide frequency. PeerJ 2015; 3: e1165.26336640

[66] Sieber CMK, Probst AJ, Sharrar A et al. Recovery of genomes from metagenomes via a dereplication, aggregation and scoring strategy. Nat Microbiol 2018; 3: 836–43. 10.1038/s41564-018-0171-1 29807988 PMC6786971

[67] Chklovski A, Parks DH, Woodcroft BJ et al. CheckM2: a rapid, scalable and accurate tool for assessing microbial genome quality using machine learning. Nat Methods 2023; 20: 1203–12. 37500759 10.1038/s41592-023-01940-w

[68] Chaumeil P-A, Mussig AJ, Hugenholtz P et al. GTDB-Tk: a toolkit to classify genomes with the genome taxonomy database. Hancock J (ed.). Bioinformatics 2020; 36: 1925–7. 10.1093/bioinformatics/btz848 PMC7703759 31730192

[69] Parks DH, Chuvochina M, Rinke C et al. GTDB: an ongoing census of bacterial and archaeal diversity through a phylogenetically consistent, rank normalized and complete genome-based taxonomy. Nucleic Acids Res 2022; 50: D785–94. 10.1093/nar/gkab776 34520557 PMC8728215

[70] Olm MR, Brown CT, Brooks B et al. dRep: a tool for fast and accurate genomic comparisons that enables improved genome recovery from metagenomes through de-replication. ISME J 2017; 11: 2864–8. 10.1038/ismej.2017.126 28742071 PMC5702732

[71] Kanehisa M, Furumichi M, Tanabe M et al. KEGG: new perspectives on genomes, pathways, diseases and drugs. Nucleic Acids Res 2017; 45: D353–61. 10.1093/nar/gkw1092 27899662 PMC5210567

[72] Aramaki T, Blanc-Mathieu R, Endo H et al. KofamKOALA: KEGG Ortholog assignment based on profile HMM and adaptive score threshold. Valencia A (ed.). Bioinformatics 2020; 36: 2251–2. 10.1093/bioinformatics/btz859 31742321 PMC7141845

[73] Makarova K, Wolf Y, Koonin E. Archaeal clusters of orthologous genes (arCOGs): an update and application for analysis of shared features between Thermococcales, Methanococcales, and Methanobacteriales. Life 2015; 5: 818–40. 10.3390/life5010818 25764277 PMC4390880

[74] Buchfink B, Reuter K, Drost H-G. Sensitive protein alignments at tree-of-life scale using DIAMOND. Nat Methods 2021; 18: 366–8. 10.1038/s41592-021-01101-x 33828273 PMC8026399

[75] Søndergaard D, Pedersen CNS, Greening C. HydDB: a web tool for hydrogenase classification and analysis. Sci Rep 2016; 6: 34212. 10.1038/srep34212 27670643 PMC5037454

[76] Lombard V, Golaconda Ramulu H, Drula E et al. The carbohydrate-active enzymes database (CAZy) in 2013. Nucl Acids Res 2014; 42: D490–5. 10.1093/nar/gkt1178 24270786 PMC3965031

[77] Zhang H, Yohe T, Huang L et al. dbCAN2: a meta server for automated carbohydrate-active enzyme annotation. Nucleic Acids Res 2018; 46: W95–101. 10.1093/nar/gky418 29771380 PMC6031026

[78] Xu J, Zhang H, Zheng J et al. eCAMI: simultaneous classification and motif identification for enzyme annotation. Xu J (ed.). Bioinformatics 2020; 36: 2068–75. 10.1093/bioinformatics/btz908 31794006

[79] Jones P, Binns D, Chang H-Y et al. InterProScan 5: genome-scale protein function classification. Bioinformatics 2014; 30: 1236–40. 10.1093/bioinformatics/btu031 24451626 PMC3998142

[80] Saier MH, Reddy VS, Moreno-Hagelsieb G et al. The transporter classification database (TCDB): 2021 update. Nucleic Acids Res 2021; 49: D461–7. 10.1093/nar/gkaa1004 33170213 PMC7778945

[81] Couvin D, Bernheim A, Toffano-Nioche C et al. CRISPRCasFinder, an update of CRISRFinder, includes a portable version, enhanced performance and integrates search for Cas proteins. Nucleic Acids Res 2018; 46: W246–51. 10.1093/nar/gky425 29790974 PMC6030898

[82] Lowe TM, Chan PP. tRNAscan-SE: On-line: integrating search and context for analysis of transfer RNA genes. Nucleic Acids Res 2016; 44: W54–7. 10.1093/nar/gkw413 27174935 PMC4987944

[83] Wu M, Scott AJ. Phylogenomic analysis of bacterial and archaeal sequences with AMPHORA2. Bioinformatics 2012; 28: 1033–4. 10.1093/bioinformatics/bts079 22332237

[84] Katoh K, Standley DM. MAFFT multiple sequence alignment software version 7: improvements in performance and usability. Mol Biol Evol 2013; 30: 772–80. 10.1093/molbev/mst010 23329690 PMC3603318

[85] Capella-Gutierrez S, Silla-Martinez JM, Gabaldon T. trimAl: a tool for automated alignment trimming in large-scale phylogenetic analyses. Bioinformatics 2009; 25: 1972–3. 10.1093/bioinformatics/btp348 19505945 PMC2712344

[86] Nguyen L-T, Schmidt HA, von Haeseler A et al. IQ-TREE: a fast and effective stochastic algorithm for estimating maximum-likelihood phylogenies. Mol Biol Evol 2015; 32: 268–74. 10.1093/molbev/msu300 25371430 PMC4271533

