# Supplementary material for: Horizontal gene transfer and gene loss drove the divergent evolution of host dependency in Micrarchaeota
Source: Natl Sci Rev. 2025 Nov 28;13(4):nwaf542. doi: 10.1093/nsr/nwaf542 (PMC12892359; doi:10.1093/nsr/nwaf542)
Supplement: nwaf542_Supplemental_Files [file nwaf542_supplemental_files.zip › Supporting information.pdf]

Supporting Information for

Horizontal gene transfer and gene loss drove the divergent evolution of host dependency in Micrarchaeota

Yang-Zhi Rao[1,2,8], Yu-Xian Li[2,3], Ze-Wei Li[2], Yan-Ni Qu[2], Brian P. Hedlund[4], Tom A. Williams[5], Yan-Ling Qi[2], Qi-Jun Xie[2], Hai-Long Yang[2], Yuan-Qi Zhang[2], Hong-Chen Jiang[6], Marike Palmer[7], Mang Shi[8], Wen-Sheng Shu[9,*], Zheng-Shuang Hua[2,*], Wen-Jun Li[1,10,*]

[1]State Key Laboratory of Biocontrol, Guangdong Provincial Key Laboratory of Plant Resources and Southern Marine Science and Engineering Guangdong Laboratory (Zhuhai), School of Life Sciences, Sun Yat-Sen University, Guangzhou 510275, China

[2] State Key Laboratory of Advanced Environmental Technology, Department of Environmental Science and Engineering, University of Science and Technology of China, 230026, China

[3]Southern Marine Science and Engineering Guangdong Laboratory (Guangzhou), Guangzhou 511458, China.

[4]School of Life Sciences, University of Nevada Las Vegas, Las Vegas, NV 89154, USA

[5] Department of Life Sciences, University of Bath, BA2 7AX, UK.

[6]Geomicrobiology Laboratory, State Key Laboratory of Geological Processes and Mineral Resources, China University of Geosciences, Beijing 100083, China.

[7]Department of Microbiology, University of Manitoba, R3T 2N2, Manitoba, Canada

[8]Centre for Infection and Immunity Study (CIIS), School of Medicine (Shenzhen), Sun Yat-sen University, Shenzhen 518107, China.

[9]School of Life Sciences, South China Normal University, Guangzhou 510275, China

[10]State Key Laboratory of Desert and Oasis Ecology, Xinjiang Key Laboratory of Biodiversity Conservation and Application in Arid Lands, Xinjiang Institute of Ecology and Geography, Chinese Academy of Sciences, Urumqi, China

***Corresponding authors:**

Wen-sheng Shu, Email: shuwensheng@m.scnu.edu.cn

Zheng-shuang Hua, Email: hzhengsh@ustc.edu.cn

Wen-jun Li, Email: liwenjun3@mail.sysu.edu.cn

This PDF file includes:

Nomenclature Appendix

Supporting text

Table S1

Figures S1 to S33

Legends for Datasets S1 to S14

SI References

Other supporting materials for this manuscript include the following:

Datasets S1 to S14

Supporting Information Text

Supporting Results

Extension of known Micrarchaeota metabolic potentials. *Fermentation.* In detail, the RuBisCO large subunits RbcL of Micrarchaeota were confirmed to phylogenetically branch between form III-b and form III-c or between form III-a and from II/III for a nucleotide salvage pathway (Fig. S5A) (1–3). On top of that, hydrogenases were also detected in all major Micrarchaeota lineages. Sequences were phylogenetically assigned to FeFe group A hydrogenase, and NiFe Group 3b hydrogenase, suggesting functioning in bidirectional NAD(P)+/NAD(P)H interconversion (4) (Fig. S5B). A complete electron transport chain (ETC) is missing in all major lineages (Fig. S6). Although Micrarchaeales encode some genes of all five complexes, only one gene *qcrB* encoding the cytochrome b subunit of complex III was detected and the iron-sulfur subunit of complex IV is also missing. In addition, Cytochrome bd complex encoded by *cydAB* may be utilized to cope with oxidative stress in acidic environments rather than aerobic respiration[7]. Altogether, consistent with prior knowledge, the anaerobic respiration of Micrarchaeota was based on the fermentation of ethanol/acetate (5–8). The encoding of alcohol dehydrogenase (*adhP*) suggests that Norongarragalinales and Micrarchaeales may be capable of ethanol fermentation, though aldehyde dehydrogenase is missing. Acetyl-CoA synthetase (*acs*) was detected in Norongarragalinales, WAL, and Anstonellales, and acetyl coenzyme A (CoA) synthetase (*acdAB*) was detected in all major lineages, which was reported to be associated with fermentation and production of acetate (5–7). However, in our analyses, we find that *acdAB* was likely to be associated with propanoate fermentation with a complete citramalate pathway encoded by Norongarragalinales, WAL, and Anstonellales (9, 10). The citramalate pathway found in this study first condenses acetyl-CoA and pyruvate to generate 2-oxobutanoate, then to propanoyl-CoA, and finally to propanoate. Relatedly, the generation of propanoate and acetate may provide substrates for syntrophic propanoate/acetate oxidizing bacteria in high-temperature environments (9). Micrarchaeota also encode CRISPR-Cas systems may enable them to serve as "viral decoys" for their potential hosts (11, 12). Micrarchaeota ARMAN-1 encoded CRISPR-Cas systems with Cas9 as the effector protein (13). However, to our surprise, CRISPR-Cas systems recovered in Micrarchaeota lineages in this study are highly diverse and show little phylogenetic conservation (Fig. S7). These may suggest that the CRISPR-Cas systems of different Micrarchaeota lineages may be horizontally transferred from each respective host. Based on annotations confirmed by searching against the Carbohydrate-active enzymes (CAZy) database (14), all major lineages possess capacities of carbohydrate biosynthesis including the synthesis of cellulose (GT2) and sucrose (GT4) (Fig. 2, Dataset S7). Albeit not detected in CAZy, Norongarragalinales, Zaosheniarchaeales, Anstonellales, and Micrarchaeales are also capable of synthesizing starch/glycogen by encoding *glgA* gene. However, only some lineages were able to degrade carbohydrates by encoding alpha-amylase (GH57, Norongarragalinales, WAL, and Anstonellales), and endoglucanase (E3.2.1.4, Redboyarchaeum). The unique cellulose degradation capacity of Redboyarchaeum may enable them to provide extra carbon sources for the cell growth of their hosts, forming potential mutually beneficial symbiosis relationships (15).

Oxidative PPP and branched Entner-Doudoroff (ED) glycolysis pathway. Possession of oxidative PPP may be an important source of NAD(P)H generation for Anstonellales (16). The *hxlB* and *hxlA* genes encoding 6-phospho-3-hexuloisomerase and 3-hexulose-6-phosphate synthase were detected in the genomes of WAL and Anstonellales, as well as other genes that encode a reverse ribulose monophosphate pathway (rRuMP) involved in pentose-phosphate biosynthesis (17, 18). As suggested previously, Micrarchaeales have near complete Entner-Doudoroff (ED) glycolysis pathway (8). Anstonellales appear to encode a branched (both semi-phosphorylative and non-phosphorylative) ED pathway that was utilized for sugar degradation (19). As for Embden-Meyerhof-Parnas (EMP) glycolysis, it is often incomplete with glucokinase missing in DPANN (20). Some rely on external sources of glycolytic intermediates to perform lower glycolysis to provide fermentation substrates. Others might bypass the first glycolytic step by the conversion of glucose-1-phosphate (G1P) degraded from polysaccharides via phosphomannomutase to glucose-6-phosphate (G6P) (11). To our surprise, Norongarragalinales and WAL are among the few DPANN lineages encoding the complete EMP pathway (21, 22). Unlike the classical EMP pathway requiring ATP-dependent glucokinase (*glk*) and PFK (*pfkA*), Norongarragalinales and WAL may employ ADP-dependent phosphofructokinase/glucokinase (*pfkC*), a bifunctional enzyme that phosphorylates both glucose and fructose-6P. The ADP-dependency of this enzyme may suggest a more efficient modified EMP pathway with higher ATP yield and increased yields of NAD(P)H and biosynthetic precursors for amino acids, nucleotides, and fatty acids (23–25). Besides the earlier description of Micrarchaeales' possession of the tricarboxylic acid (TCA) cycle (8), genomes of Norongarragalinales were also detected to contain a full set of genes involved in the TCA cycle. Genes associated with the Wood-Ljungdhal pathway (WLP) were also detected in Micrarchaeota genomes but were scattered in different lineages. Although Norongarragalinales, WAL and Anstonellales have most of the associated genes, the key acetyl-CoA synthase (ACS) gene *acsABC* is missing. Micrarchaeales on the contrary, only possess *acsC* gene of one ACS subunit. Thus, none of the Micrarchaeota lineages has a complete WL pathway for carbon fixation. The absence of genes linked to the archaeal type WLP and the presence of genes related to the tetrahydrofolate (THF) methyl-branch acetogenesis in bacteria suggests that these traits may have been acquired from bacteria through HGTs (26).

Sulfur and nitrogen metabolism. Micrarchaeota also encoded numerous genes involved in sulfur and nitrogen metabolisms. Members of Zaosheniarchaeales may be capable of dissimilatory sulfur reduction (DSR) of sulfate to sulfite by encoding adenylyltransferase (*sat*), adenylylsulfate reductase (*aprAB*), and quinone-modifying oxidoreductase (*qmoAC*). Genes encoding dissimilatory sulfite reductase (*dsrAB*) were absent. However, *dsrAB* were previously detected in Thermoplasmatota (27, 28), which is also shown to be the host of Micrarchaeales (29, 30). Thus, Zaosheniarchaeales may provide their hosts with sulfite as substrates for the DSR cascade. Other lineages including WAL, Anstonellales, and Micrarchaeales possess sulfide:quinone oxidoreductase (*sqr*) genes that may endow them with the ability to oxidize sulfide to sulfur. Interestingly, Anstonellales appear to participate in the denitrification process by encoding nitrate reductase (NR), and nitrite reductase (*nirK*). Moreover, similar to Jingweiarchaeales and Parvarchaeales, they encoded hydrazine

synthase (HZS, K20934) that is involved in the first step of anaerobic ammonium oxidation (anammox), converting ammonia and nitric oxide to hydrazine (31, 32). Genomes of other Micrarchaeota lineages, including Norongarragalinales, Zaosheniarchaeales, and Micrarchaeales, encoded another subunit of HZS (K20932). This is unexpected since anammox capacity was only previously detected in bacteria (33). However, since none of the genomes encoded all subunits of HZS, further experimental validation is necessary to illustrate the role of DPANN archaea in anammox processes.

Habitat transition-driven genome expansion. In the most metabolically diverse Micrarchaeota lineage that may even be free-living, gene gains in the last common ancestor of Norongarragalinales (NG LCA) were diminished by gene loss (Fig. 4, A and B). Further results suggested that the genome expansion of this order was after the diversification of NG LCA with multiple HGT hot spots (Fig. S13, Dataset S12). The proteome sizes were also expanded on the last common ancestor of Tudiarchaeales, Zaosheniarchaeales, WAL, Gugararchaeales, Anstonellales, and Micrarchaeales (TZWGAM LCA), the last common ancestor of Tudiarchaeales, Zaosheniarchaeales, WAL, Gugararchaeales, and Anstonellales (TZWGA), the last common ancestor of WGA LCA and to WAL LCA and the last common ancestor of Gugararchaeales (GA LCA) and AT LCA. These expansions may endow associated lineages with a putative transition from obligate symbiosis to a less host-dependent lifestyle.

Supporting Methods

Detailed descriptions of sampling sites, DNA extraction, and metagenomic sequencing

A total of 160 metagenomic samples were collected from 5 regions in Yunnan province and Xizang autonomous region, China, from January 2016 to January 2021. Sites in Xizang autonomous region: (1) DaGeJia ChangMaQu (DGJ) east coast, AngDa county: DGJ08. (2) QuCai (QC) village, DangXiongQuCai county: QC03. (3) QuZhuoMu (QZM) village, CuoNa county: QZM_A1, QZM_A2, QZM_A3, QZM_B1, QZM_B3, QZM_B4, QZM_D2, and SM4. Sites in Tengchong, Yunnan: (1) Rehai Geothermal National Park: i) DiReChi (DRC). ii) DiReTiYan Zone (DRTY): DRTY-1, DRTY-2, DRTY-3, DRTY-4, DRTY-5, DRTY-6, DRTY-7, DRTY-8, DRTY-9, DRTY-10, DRTY-16, DRTY-18, and DRTY-19. iii) GuMingQuan (GMQ): GMQP and GMQS. iv) QiaoQuan (QQ). v) ShuiReBaoZha Zone (SRBZ) - SRBZ-1 and SRBZ-2. vi) DaPingZi (DPZ): DPZ-2 and DPZ-5. vii) ShiZiTouDuiMian (SZTDM): SZTDM, SZTDM-1b, SZTDM-1c, and SZTDM-2. viii) ZiMeiQuan (ZMQ): ZMQL and ZMQR. xi) ZhenZhuQuan (ZZQ): ZZQ and ZZQ-2 (2) Diantan county: i) JinZe Hot Spring Resort (JZ) - JZ-1, JZ-2, JZ-3 and JZ-4. ii) GongXiaoShe (GXS). (3) Others: BianFuWa (BFW): BF2, BF3, HHBFW-2; HaMaZui (HMZ); HeiNiTan (HNT): HNT-1, HNT-1-1, HNT-1-2, HNT-2, HNT-3, HNT-5, HNT-7, HNT-8, and HNT-10.

The sampling date, GPS coordinates, and geochemical characteristics of all sample sites were recorded in detail in Dataset S2 and SI. Hot spring samples were collected in the field into 50 ml of sterilized centrifugation tubes, and community DNA was extracted from ~20g of sediment samples using the PowerSoil DNA isolation kit (MiBio). The DNA quality was evaluated by the Qubit fluorimeter. The DNA libraries

were constructed with an average insert size of 350 bp using an M220 Focused-ultrasonicator NEBNext and an Ultra II DNA library prep kit. Metagenomic sequencing was performed with the Illumina Hiseq 4000 platform at Beijing Novogene Bioinformatics Technology Co., Ltd (Beijing, China), generating approximately 30 giga base pairs (Gbp) of PE250 reads for each sample.

Phylogenetic analyses

Selection of reference genomes and phylogenetic markers in Fig. 2. The 53 archaeal markers were initially derived from the Undinarchaeota study [34]. This study extensively evaluated 151 markers, including the previous 120 GTDB archaeal markers (r89), Phyloswift markers, and newly identified ones. It revealed that many of the original 120 GTDB markers and others were inappropriate as they failed to recover the monophyly of archaea or well-established archaeal lineages. The resulting refined 56-marker set, with the top-ranked 50% of markers, was recommended and was later modified to 53 markers and adopted by GTDB in r207 (https://forum.gtdb.ecogenomic.org/t/gtdb-r07-rs207-release- notes/265). We have shown that most of these markers were present on over 80% of the genomes of each phylum (Dataset S5)

RuBisCo and hydrogenases tree. RuBisCo reference sequences were selected from a previous study [35]. These sequences were aligned with MAFFT (v7.487) [36] with "linsi" option with 1000 iterations. Poorly aligned regions were removed using TrimAl (v1.4. rev22). The unrooted phylogeny was generated using IQ-TREE with the following parameters: -alrt 1000 -bb 1000 -m JTT. The best-fitted model of the phylogenetic tree is LG+R8. Reference sequences of hydrogenases were acquired from HydDB [37], and the phylogenetic construction workflow is the same as RuBisCo. The best-fitted model of the hydrogenase's phylogeny is LG+R10.

Comparative genomics analyses

Amino acid identity (AAI) between each genome pair was determined by calculating the mean identities of orthologs, which were extracted from all reciprocal best BLAST hits (rBBHs; E-value < 1e-10). The usage of amino acid frequencies was calculated by a self-made Perl script (amino_acid_usage.pl, https://github.com/hzhengsh/reverse_ecology). Heatmaps of AAI and amino acids usage of representative genomes were generated by the pheatmap package (v1.0.12) [38] in R. Rate smoothing of the phylogenetic tree on the heatmaps was conducted with makeChronosCalib() and chronos() functions of the ape package (v5.6-2) [39]. The optimal growth temperatures (OGT) were calculated using the OGT_prediction program [40] and amino acid frequencies of seven amino acids (IVYWREL, IVYWREL2_4_fasta_dir.pl, https://github.com/hzhengsh/reverse_ecology) [41]. Genomic feature differences and amino acid frequency differences were tested by ANOVA with function aov() in R package stats (v4.2.2) [42], and HSD post hoc all-pairwise comparisons test [43] was conducted by function HSD.test in R package agricolae (v1.3-2) [44]. The ggplot2 package (v3.4.1) [45] was used to conduct and visualize linear regressions of genomic features with geom_smooth (method = "lm") function. The t-distributed stochastic neighbor embedding (t-SNE)

analysis [46] was further implemented for ordination with Rtsne() function in Rtsne package (v0.16, https://github.com/jkrijthe/Rtsne). Only 68 representative MAGs were included for clearance of ordinations. Genomic feature differences, amino acid frequency differences, and ordinations were visualized by ggplot2 package (v3.4.1) [45]. Redundancy analysis was performed using rda() function in the vegan package (v2.6-6.1). Only genomes recovered from Yunnan were kept for analysis to maximize the available geochemical parameters. Co-linear parameters were removed before analysis.

Evolutionary genomics

Species tree for evolutionary history inference. To conduct a reliable evolutionary history inference of Micrarchaeota, only genomes with completeness $\geq$90% and contamination < 5% were kept for further analyses. This results in 494 genomes kept from 658 genomes of previous phylogenomic analyses in Fig. 2. Genomes that do not meet this standard were removed from the previously generated phylogenomic species tree via the tips.drop() method and schematic trees were converted by rate-soothing using the makeChronosCalib() and chronos() functions of the *ape* package (v5.7.1) of R [39]. To ameliorate the effect of long branch attraction (LBA), we additionally used site-heterogeneous substitution models on the multiple sequence alignment (MSA) to infer the species tree using IQ-TREE (settings: -s all.pro.gtdb.ar53.markers.evol.phy -m MFP -mset LG -madd LG+C20,LG+C40,LG+C60 -B 1000 -wbtl). To directly evaluate support for an alternative hypothesis of species relationships that bears on the question of genome size evolution within DPANN, we used a constrained tree search in IQ-TREE to obtain the maximum likelihood tree in which Altiarchaeota branched sister to all other DPANN. Then, an approximately unbiased (AU) test implemented in IQ-TREE was used to determine the confidence set of tree topologies that could not be rejected by the data at $P < 0.05$. To further ensure the robustness of the species tree used for evolutionary history inference, we tested different marker sets: 1) 16 RPs [5, 20, 47, 48], 2) RP55 [49], and 3) 48 arCOGs [50]. Markers present in fewer than 50% of the genomes within each phylum except Huberarchaeota were excluded (Dataset S9), resulting in 14 RPs, 49 RPs, and 45 arCOGs. The same procedure of phylogenetic tree construction of the 53 GTDB archaeal marker set was used for phylogenetic inferences of these marker sets, retaining 2,284, 7,310, and 10,107 amino acid positions for phylogenetic construction, respectively.

All protein sequences of Micrarchaeota MAGs were assigned to protein families according to arCOG annotation. For some of the protein functions of interest that have sequences that lack arCOG assignment but with KO assignment, protein families were assigned based on the KO annotation. Only the protein families with no less than four sequences were subject to further analyses, and sequences of each family were extracted for phylogenetic analyses using the same parameters of phylogenomic analysis. The best-fitting model of each phylogenetic tree is listed in Dataset S11. To mitigate potential LBA, we conducted analyses based on the posterior mean site frequency (PMSF) model for each gene family tree. The constructed tree of each family was subsequently passed to the gene tree-aware approach implemented by the ALE package (v0.5)

[51, 52]. The ALEobserve and ALEml_updated (sample=100) algorithm in ALE was applied to compute and reconcile the protein family trees probabilistically against the species tree and further infer the numbers of gene presence, duplications, losses, intra-HGTs (HGTs identified within the genome set of this analysis), and originations (including gene birth and HGTs with donors from outside of this genome set) of each branch on the species tree with a threshold of 0.3 for output. The evolutionary "hot spots" were designated to tree nodes with the top 50 counts of the respective evolutionary events. The potential donors of HGTs were quantified by a previously published Python script (count_sister_taxa.py, https://github.com/Tancata/phylo/) [34] based on well-established GTDB phyla. However, lineages proposed or of concern (e.g., Norongarragalinales, Tudiarchaeales, Zaosheniarchaeales, Wunengiarchaeales associated lineages, Gugararchaeales, Anstonellales, Micrarchaeales, and Redboyarchaeum) within Micrarchaeota in this study were inspected to uncover the sources of HGTs of each lineage.

Nomenclature Appendix

The etymology of *Tudiarchaeum tengchongense* sp. nov.

Tudiarchaeum tengchongense (teng.chong.en'se N.L. neut. adj. *tengchongense*, referring to Tengchong county, Yunnan Province, China, where the first genome of this species was recovered from). The nomenclatural type for the species is DRTY-6_201705_bins_182.

The etymology of *Tudiarchaeum* gen. nov.

Tudiarchaeum (Tu.di.ar.chae'um N.L. masc. n. *Tudius*, from Mandarin, Tudi or Tudi Gong, the Earth God in traditional Chinese folklore, a guardian deity who protects the locality and inhabitants for prosperity, protection, and peace; N.L. neut. n. *archaeum*, an archaeon, ancient; N.L. neut. n. *Tudiarchaeum*, an archaeon named for Tudi (Gong) a companion and guardian to the local microbial community. The predicted role for these archaea is similar to DPANN archaea's role in forming a mutually beneficial symbiosis with hosts and contributing to a special niche in the microbial community despite the tiny cell sizes and limited metabolic capacities. The nomenclatural type for the genus is the species *Tudiarchaeum tengchongense*.

The etymology of Tudiarchaeaceae fam. Tudiarchaeaceae (Tu.di.ar.chae.a.ce'ae. N.L. neut. n. *Tudiarchaeum* a genus; -*aceae*, ending to denote a family; N.L. fem. pl. n. Tudiarchaeaceae, the *Tudiarchaeum* family). The nomenclatural type for the family is the genus *Tudiarchaeum*.

The etymology of Tudiarchaeales ord. nov.

Tudiarchaeales (Tu.di.ar.chae.a'les. N.L. neut n. *Tudiarchaeum* a genus; -*ales*, ending to denote an order; N.L. fem. pl. n. Tudiarchaeales the *Tudiarchaeum* order). The nomenclatural type for the order is the genus *Tudiarchaeum*.

The etymology of *Zaosheniarchaeum subthermophilum* sp. nov.

Zaoshenarchaeum subthermophilum (sub.ther.mo'phi.lum. Gr. prefix *sub*-, somewhat; Gr. fem. n. *therme*, heat; Gr. masc. adj. *philos*, loving; N.L. neut. adj. *subthermophilum*, somewhat heat-loving. *Zaosheniarchaeum*). This organism was found in sampling sites with elevated but not extreme temperatures,

and potentially, this genus has characteristics of a moderately high estimated optimal growth temperature (OGT). The nomenclatural type for the species is the genome JZ-3_201901_bins_92.

The etymology of *Zaosheniarchaeum* gen. nov.

Zaosheniarchaeum (Zao.shen.i.ar.chae'um N.L. masc. n. *Zaoshenius*, from Mandarin Zaoshen, the Stove God in traditional Chinese folklore, oversees a family's moral integrity and influences their fortune based on ethical conduct; N.L. neut. n. *archaeum*, an archaeon, ancient; N.L. neut. n. *Zaosheniarchaeum*, an archaeon named after Zaoshen, for the protection of the family. The predicted role for these organisms is similar to the small but indispensable role of DPANN archaea. The nomenclatural type of the genus is the species *Zaosheniarchaeum subthermophilum*.

The etymology of Zaosheniarchaeaceae fam. nov.

Zaosheniarchaeaceae (Zao.shen.i.ar.chae.a.ce'ae. N.L. neut. n. *Zaosheniarchaeum* a genus; -*aceae*, ending to denote a family; N.L. fem. pl. n. Zaosheniarchaeaceae, the *Zaosheniarchaeum* family). The nomenclatural type for the family is the genus *Zaosheniarchaeum*.

The etymology of Zaosheniarchaeales ord. nov.

Zaosheniarchaeales (Zao.shen.i.ar.chae.a'les. N.L. neut n. *Zaosheniarchaeum* a genus; -*ales*, ending to denote an order; N.L. fem. pl. n. Zaosheniarchaeales the *Zaosheniarchaeum* order). The nomenclatural type for the order is the genus *Zaosheniarchaeum*.

The etymology of *Wunengiarchaeum polytrophicum* sp. nov.

Wunenigarchaeum polytrophicum (po.ly.tro.'phi.cum Gr. neut. adj. *poly*, many; Gr. neut. n. *trophe*, nourishment; N.L. neut. adj. *polytrophicum*, utilizing many types of nutrients). Genomes of members of *Wunengiarchaeum* encode substantial metabolic capacity by utilizing a broad spectrum of organic and inorganic substrates. The nomenclatural type for the species is DRTY-6_201705_bins_86.

The etymology of *Wunengiarchaeum* gen. nov.

Wunengiarchaeum (Wu.nen.gi.ar.chae'um. N.L. masc. n. *Wunengius*, from Mandarin, Wuneng, the formal Buddhist name of Zhu Bajie, a character renowned for his mixed capabilities and shortcomings in the Chinese literary classic "Journey to the West."; N.L. neut. n. *archaeum*, an archaeon, ancient; N.L. neut. n. *Wunengiarchaeum*, the archaeon was named after Wuneng for their metabolic capacity, and ability to thrive

in diverse environments mirroring Zhu Bajie's ability to overcome various trials despite his flawed and often comical nature. The nomenclatural type of the genus is the species *Wunengiarchaeum polytrophicum*.

The etymology of Wunengiarchaeaceae fam. nov.

Wunengiarchaeaceae (Wu.nen.gi.ar.chae.a.ce'ae. N.L. neut. n. *Wunengiarchaeum* a genus; -*aceae*, ending to denote a family; N.L. fem. pl. n. Wunengiarchaeaceae, the *Wunengiarchaeum* family). The nomenclatural type of the family is the genus *Wunengiarchaeum*.

The etymology of Wunengiarchaeales ord. nov.

Wunengiarchaeales (Wu.nen.gi.ar.chae.a'les. N.L. neut n. *Wunengiarchaeum* a genus; -*ales*, ending to denote an order; N.L. fem. pl. n. Wunengiarchaeales the *Wunengiarchaeum* order). The nomenclatural type of the order is the genus *Wunengiarchaeum*.

The etymology of *Bailongiarchaeum tengchongense* sp. nov.

Bailongarchaeum tengchongense (teng.chong.en'se. N.L. neut. adj. *tengchongense*, referring to Tengchong county, Yunnan Province, China, where its first genome was reconstructed). The nomenclatural type of the species is the genome QQ_201907_bins_60.

The etymology of *Bailongiarchaeum* gen. nov.

Bailongiarchaeum (Bai.lon.gi.ar.chae'um. N.L. masc. n. *Bailongius*, from Mandarin, Bailong, the formal name of the White Dragon Horse from the Chinese literary classic "Journey to the West," a character known for his transformation from a dragon into a horse to serve as Xuanzang's loyal steed on the journey; N.L. neut. n. *archaeum*, an archaeon, ancient; N.L. neut. n. *Bailongiarchaeum*, an archaeon named after Bailong for its adaptability and resilience to thrive in various environments with metabolic diversity. The nomenclatural type for the genus is the species *Bailongiarchaeum tengchongense*.

The etymology of Bailongiarchaeaceae fam. nov.

Bailongiarchaeaceae (Bai.lon.gi.ar.chae.a.ce'ae. N.L. neut. n. *Bailongiarchaeum* a genus; -*aceae*, ending to denote a family; N.L. fem. pl. n. Bailongiarchaeaceae, the *Bailongiarchaeum* family). The nomenclatural type for the family is the genus *Bailongiarchaeum*.

The etymology of Bailongiarchaeales ord. nov.

Bailongiarchaeales (Bai.lon.gi.ar.chae.a'les. N.L. neut n. *Bailongiarchaeum* a genus; -*ales*, ending to denote an order; N.L. neut. pl. n. Bailongiarchaeales the *Bailongiarchaeum* order). The nomenclatural type of the order is the genus *Bailongiarchaeum*.

The etymology of *Wujingiarchaeum fermentans* sp. nov.

Wujingiarchaeum fermentans (fer.men'tans. L. part. adj. *fermentans*, fermenting). This novel species lack a respiratory chain and may entirely rely on fermentation for growth. The nomenclatural type for the species is the genome DRTY-6_201901_bins_19.

The etymology of *Wujingiarchaeum* gen. nov.

Wujingiarchaeum (Wu.jin.gi.ar.chae'um N.L. masc. n. *Wujingius*, from Mandarin, Wujing, the formal Buddhist name of Sha Wujing, a character known for his steadfast loyalty and strength in the Chinese literary classic "Journey to the West."; N.L. neut. n. *archaeum*, archaeon, ancient; N.L. neut. n. *Wujingiarchaeum*,

the archaeon was named after Wujing for its resilience and ability to thrive under harsh conditions). The etymology of the genus name refers especially to is survival in harsh conditions in aquatic environments, since most genomes were shown to be recovered from freshwater habitats. The nomenclatural type of the genus is the species *Wujingiarchaeum fermentans*.

The etymology of Wujingiarchaeaceae fam. nov.

Wujingiarchaeaceae (Wu.jin.gi.ar.chae.a.ce'ae. N.L. neut. n. *Wujingiarchaeum* a genus; *-aceae*, ending to denote a family; N.L. fem. pl. n. Wujingiarchaeaceae, the *Wujingiarchaeum* family). The nomenclatural type of the family is the genus *Wujingiarchaeum*.

The etymology of Wujingiarchaeales ord. nov.

Wujingiarchaeales (Wu.jin.gi.ar.chae.a'les. N.L. neut n. *Wujingiarchaeum* a genus; *-ales*, ending to denote an order; N.L. neut. pl. n. *Wujingiarchaeales* the *Wujingiarchaeum* order). The nomenclatural type of the order is the genus *Wujingiarchaeum*.

The etymology of *Redboyarchaeum thermophilum* sp. nov.

Redboyarchaeum thermophilum (ther.mo'phi.lum. Gr. fem. n. therme, heat; Gr. masc. adj. philos, loving; N.L. neut. adj. thermophilum, heat loving. *Redboyarchaeum* was found in sampling sites of ultra-high temperatures and potentially high estimated OGT). The nomenclatural type is the genome BF3_201808_bins_80.

The etymology of *Redboyarchaeum* gen. nov.

Redboyarchaeum (Red.boy.archae'um. N.L. masc. n. *Redboyus*, from English "Red Boy", translation from "Hong Haier" in Mandarin, the son of the Bull Demon King and Princess Iron Fan is initially a formidable antagonist turned disciple in the Chinese literary classic "Journey to the West."; N.L. neut. n. *archaeum*, archaeon, ancient; N.L. neut. n. *Redboyarchaeum* an archaeon named after Red Boy. Genomes of organisms are predicted to possess high estimated OGT, mirroring Red Boy's possession of powerful fire magic. The nomenclatural type for the genus is the species *Redboyarchaeum thermophilum*.

All proposed names were registered in SeqCode (53).

Table S1. Output of approximately-unbiased (AU) test

Tree	logL	deltaL	bp-RELL	p-KH	p-SH	c-ELW	p-AU
ar53_mixture_model	-5763125.9	0	1 +	1 +	1 +	1 +	1 +
ar53_alti_basal_cons	-5764269.3	1143.5	0 -	0 -	0 -	3.02e-153 -	3.32e-54 -

*deltaL: logL difference from the maximal logl in the set.
*bp-RELL: bootstrap proportion using RELL method (Kishino et al. 1990).
*p-KH: p-value of one sided Kishino-Hasegawa test (1989).
*p-SH: p-value of Shimodaira-Hasegawa test (2000).
*c-ELW: Expected Likelihood Weight (Strimmer & Rambaut 2002).
*p-AU: p-value of approximately unbiased (AU) test (Shimodaira, 2002).

*Plus signs denote the 95% confidence sets.
*Minus signs denote significant exclusion.
*All tests performed 10000 resamplings using the RELL method.

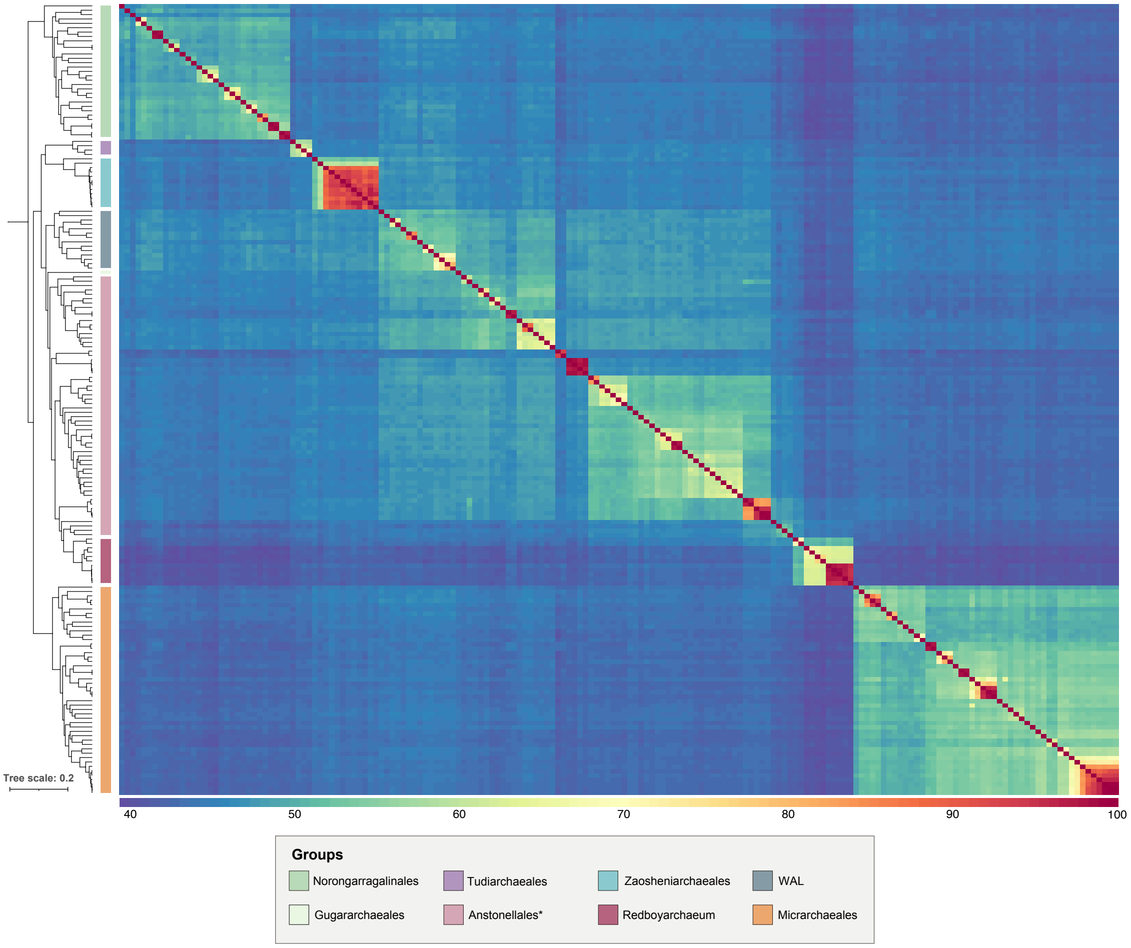

Fig. S1. Average amino acid identity (AAI) hierarchical heatmap. The dendrogram shown on the left was extracted from the maximum likelihood tree based on 53 concatenated GTDB conserved marker genes in Fig. 1B. Asterisks indicate that Redboyarchaeum were separately counted from other Anstonellales genomes.

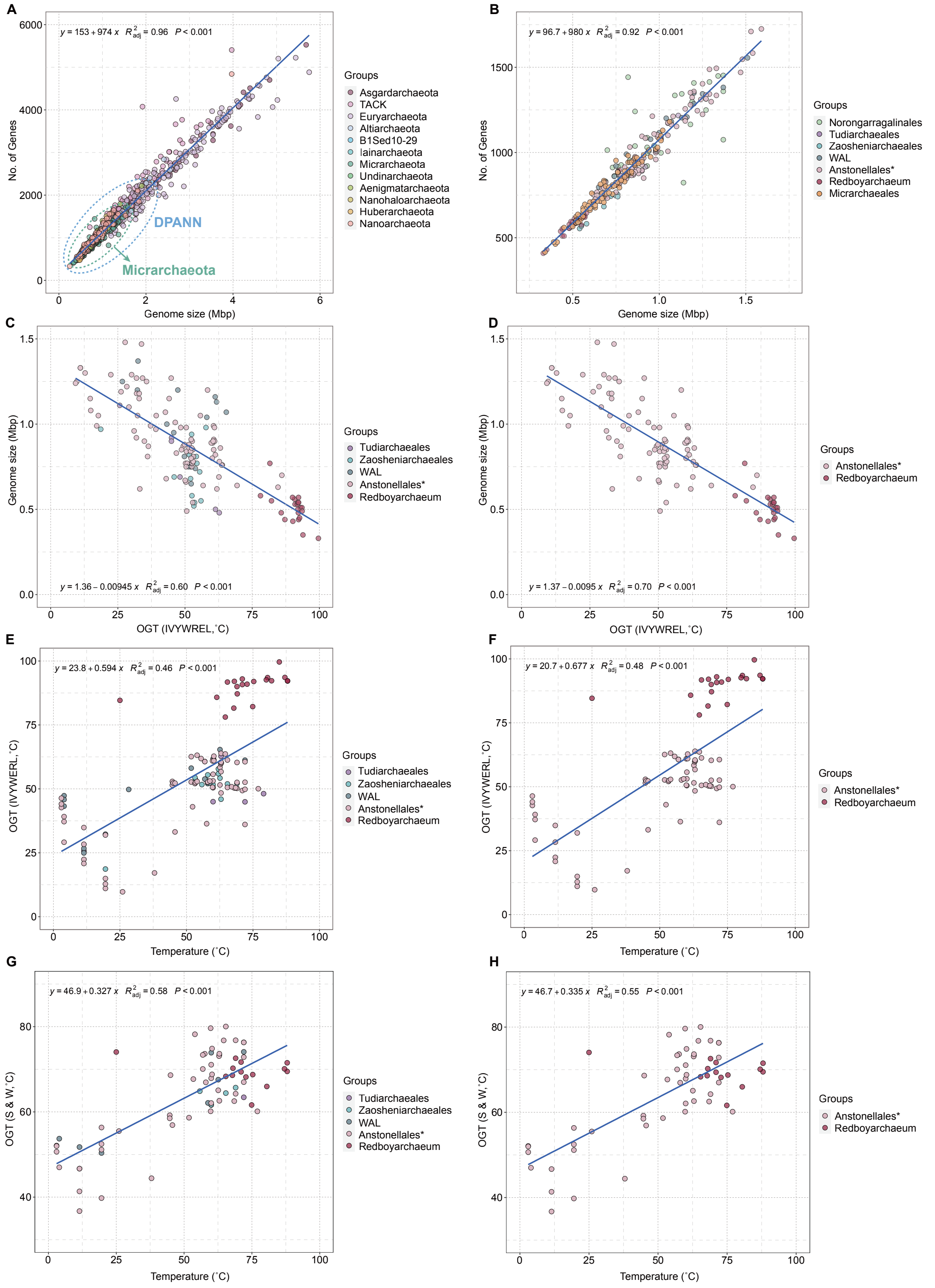

Fig. S2. Linear regression between the number of protein-coding genes and genome sizes, between genome sizes and optimal growth temperature (OGT), and between sampling temperatures and OGTs. (A) Linear regression between genome sizes and number of protein-coding genes among Micrarchaeota and other archaeal phyla. (B) Linear regression between genome sizes and number of protein-coding genes among

Micrarchaeota. (C) Linear regression between OGTs and genome sizes among Tudiarchaeales, Zaosheniarchaeales, Wunengiarchaeales associated lineages (WAL), and Anstonellales. (D) Linear regression between OGTs and genome sizes among Anstonellales. (E) and (F) Linear regression between sampling temperatures and OGTs using IVYWERL estimation (see methods for detail) (41) among Tudiarchaeales, Zaosheniarchaeales, WAL, and Anstonellales (E), and within the Anstonellales (F). (G) and (H) Linear regression between sampling temperatures and OGTs using the estimation of Sauer and Wang. (S & W, see methods for details) (40) among Tudiarchaeales, Zaosheniarchaeales, WAL, and Anstonellales (G) and within Anstonellales (H). Asterisks indicate that Redboyarchaeum were separately counted from other Anstonellales genomes.

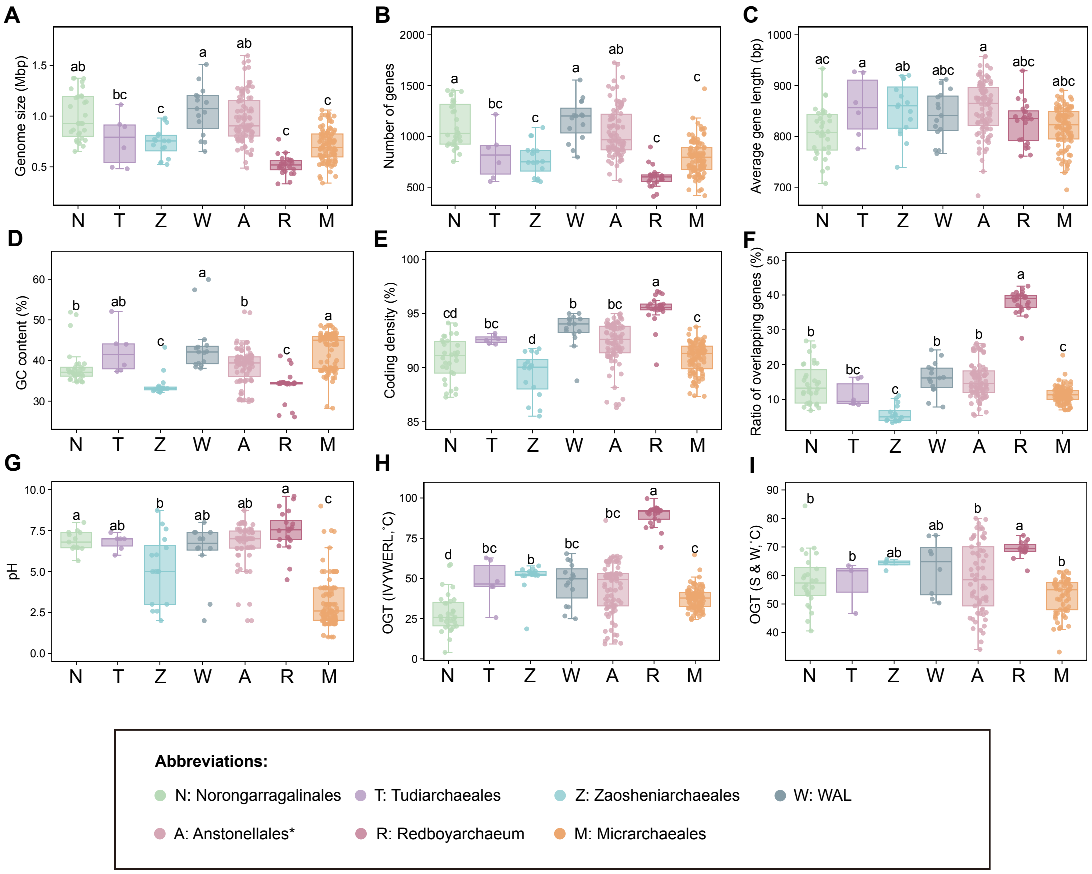

Fig. S3. Genomic comparisons of major lineages of Micrarchaeota. Boxplots of (A) genome sizes, (B) the number of genes, (C) average gene length, (D) GC content, (E) coding density, (F) ratio of overlapping genes, (G) pH values of derived samples, (H) OGTs predicted by the overrepresentation of 7 amino acids (IVYWREL) (41), (I) OGTs predicted with methods proposed by Sauer and Wang (40), were visualized using ggplot2 package v3.3.6 in R (45). The HSD post hoc all-pairwise comparisons test was used to test the differences in genomic characters among different groups. Different letters show significant differences between groups with a P value of < 0.05. Abbreviations: WAL - Wunengiarchaeales associated lineages (WAL); S & W - Sauer and Wang.

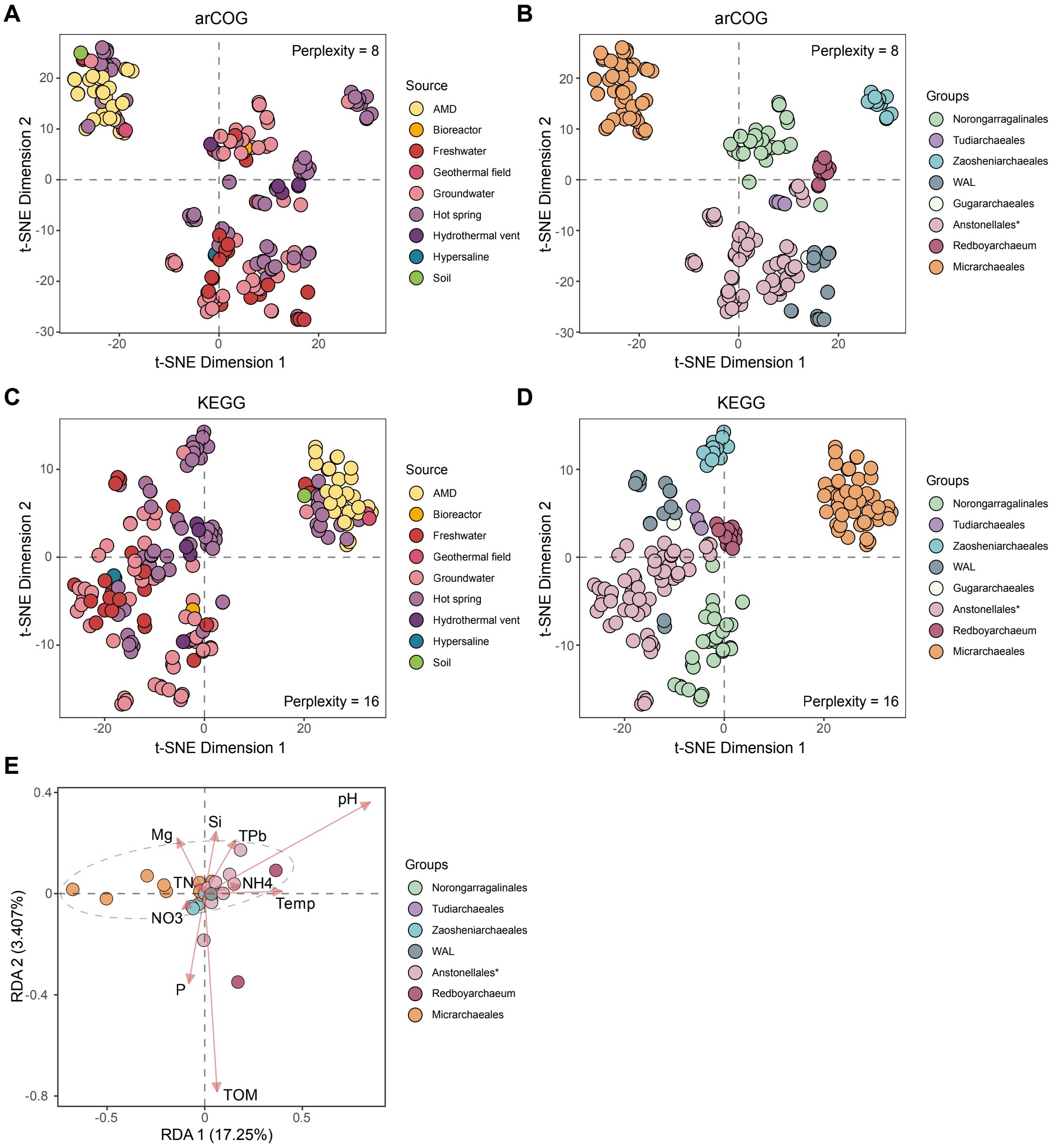

Fig. S4. t-Distributed Stochastic Neighbor Embedding (t-SNE) of Micrarchaeota and redundancy analysis across all sites in Yunnan. (A) and (B) were plotted with the copy of functional genes of 68 representative MAGs and reference genomes based on the annotation of arCOG databases, and (C) and (D) were plotted based on the annotation of KEGG databases. (E) were plotted based on 37 Micrarchaeota MAGs with relative abundances > 0.01%. Colors in (A) and (C) represent the grouping by habitats of Micrarchaeota, and (B), (D), and (E) represent the major lineages of Micrarchaeota. WAL in abbreviation stands for Wunengiarchaeales-associated lineages.

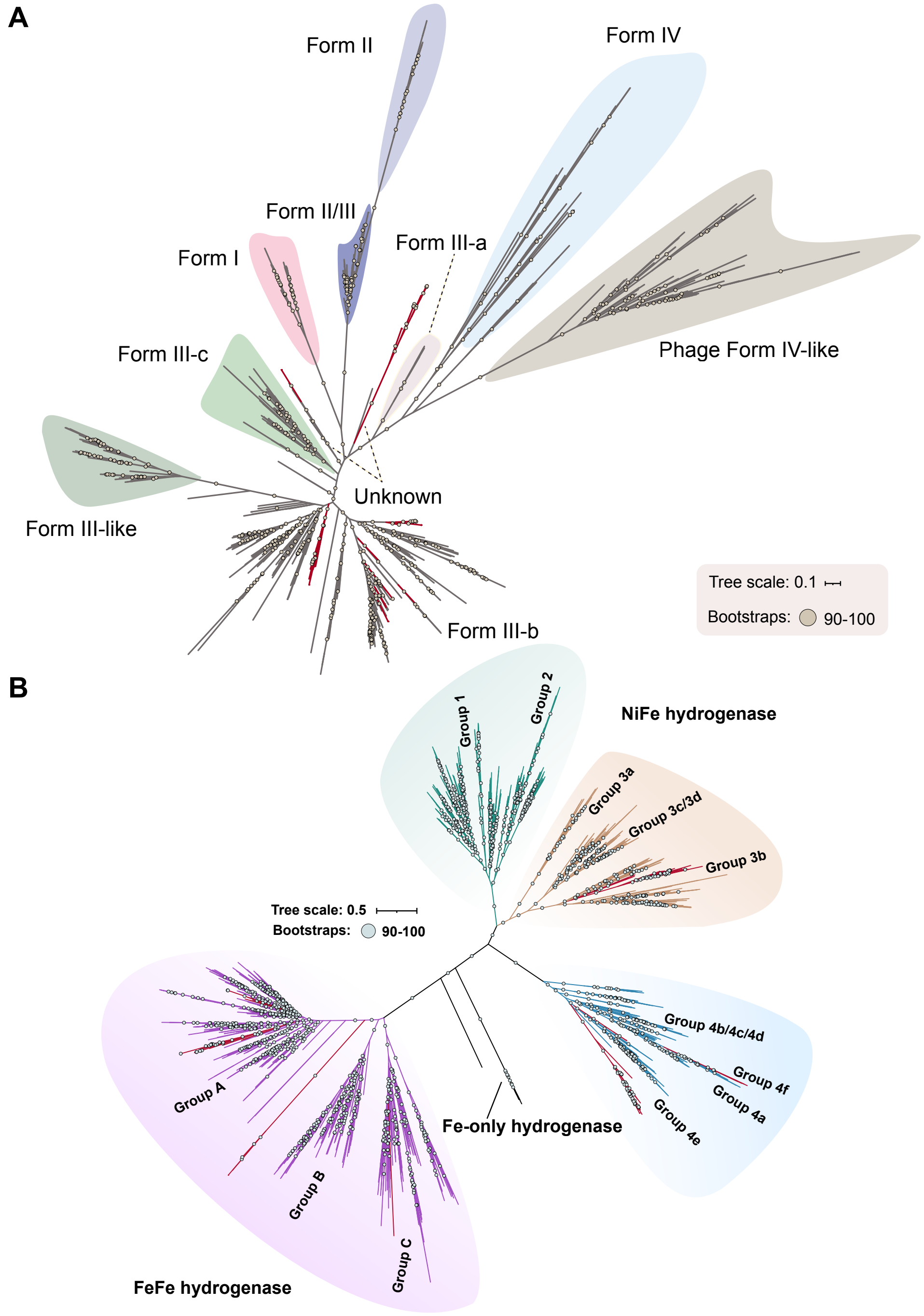

Fig. S5. Maximum likelihood phylogenetic of the ribulose-1,5-bisphosphate carboxylase-oxygenase (RuBisCO) and hydrogenases. (A) Maximum likelihood phylogenetic tree of RuBisCO. Sequences of Micrarchaeota MAGs in this study are shown in red branches. Bootstrap values of 90-100% were shown in light brown circles. (B) Hydrogenases. Sequences of Micrarchaeota retrieved in this study are shown in red clades. Shades in light green, light brown, and light blue are [NiFe]-hydrogenases, and shades in purple are [FeFe]-hydrogenases. Bootstraps support of 90-100 were shown in blue circles.

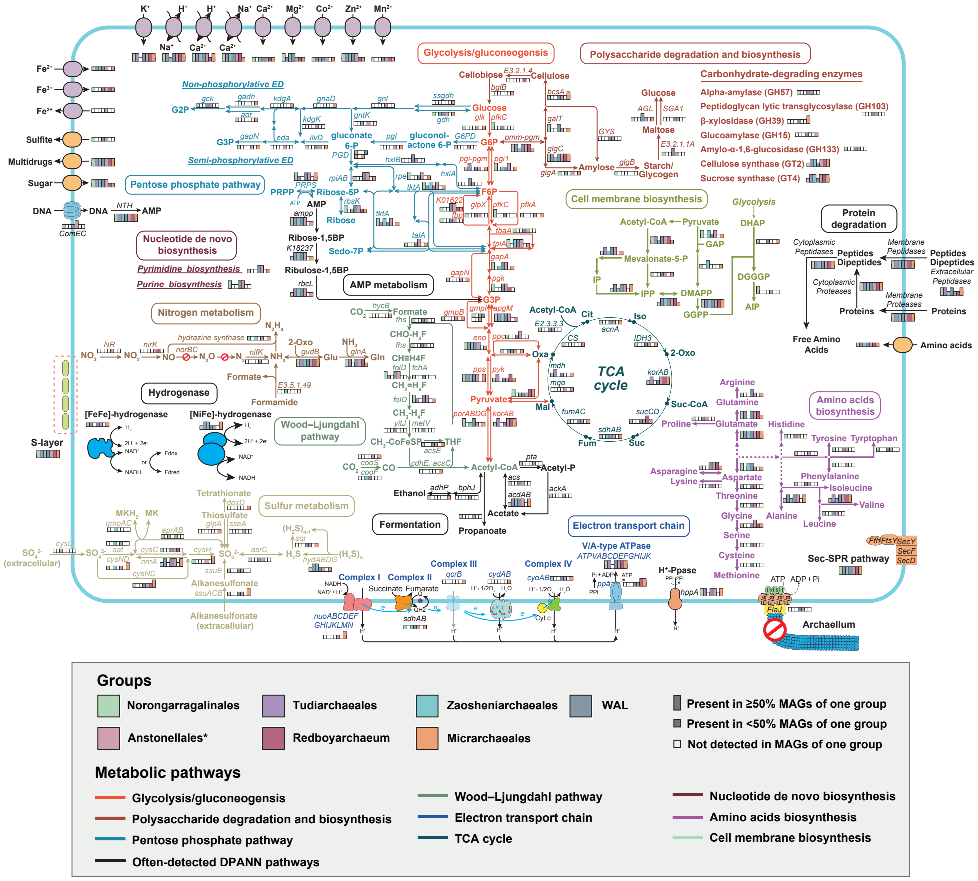

Fig. S6. Metabolic potentials in detail of Micrarchaeota. The presence of genes in different major lineages of Micrarchaeota is shown in different color symbols, respectively. Genes that are present in no less than 50% of the genomes are illustrated in complete rectangles, genes present in less than 50% but at least one MAG are shown in half rectangles, and genes that were not detected in one group are shown in hollow half rectangles. Different metabolic pathways were indicated in lines of different colors. Asterisks indicate that Redboyarchaeum was excluded from the statistics of other Anstonellales genomes. Abbreviations: G6P, glucose-6-phosphate; F6P, fructose-6-phosphate; G3P, glycerate-3-phosphate; G2P, glycerate-2-phosphate; Ribose-5P, ribose-5-phosphate; Sedo-7P, sedoheptulose-7-phosphate; PRPP, phosphoribosyl diphosphate; gluconate 6-P, 6-phosphogluconate; gluconolactone 6-P, 6-phosphogluconolactone; Mevalonate-5-P, mevalonate phosphate; IP, isopentenyl phosphate; IPP, isopentenyl diphosphate; DMAPP, dimethylallyl pyrophosphate; GGPP, geranylgeranyl diphosphate; DHAP, Dihydroxyacetone phosphate; DGGGP, digeranylgeranylglyceryl phosphate; AIP, archaetidylinositol phosphate; Oxa, oxaloacetate; Cit, citrate; Iso, isocitrate; 2-Oxo, 2-oxoglutarate; Suc-CoA, succinyl- CoA; Suc, succinate; Fum, fumarate; Mal, malate; TCA, tricarboxylic acid; MK, menaquino.

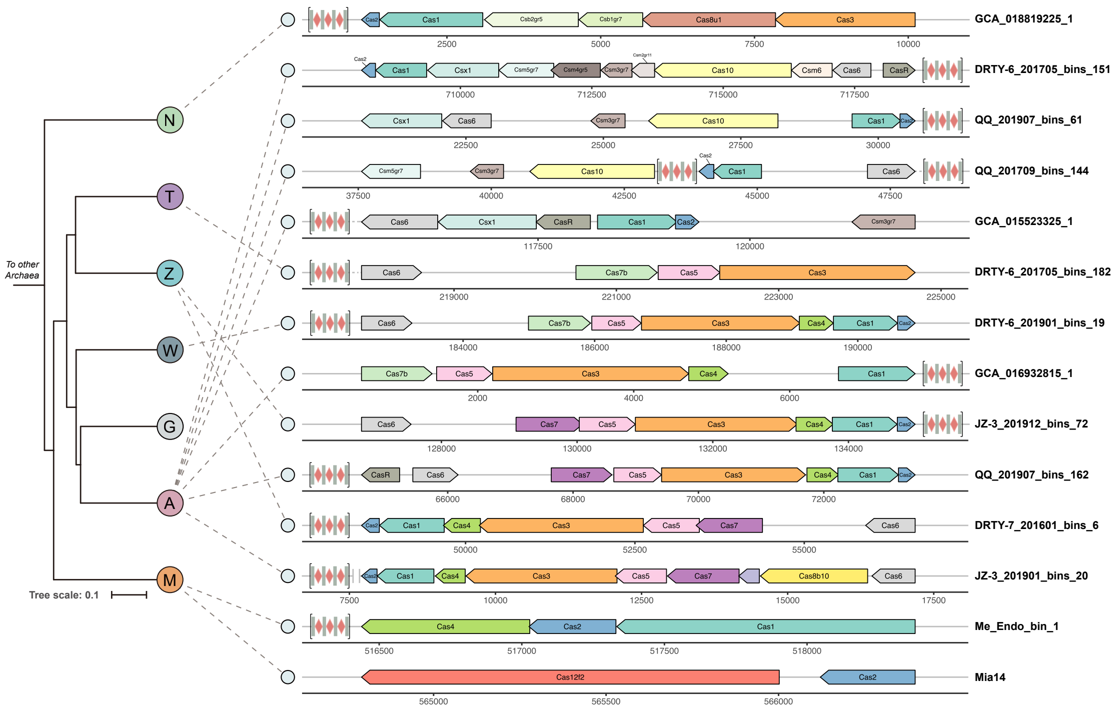

Fig. S7. Gene organizations of CRISPR-Cas systems detected in 163 MAGs and the reference genomes of Micrarchaeota in this study. The schematic tree on the left was trimmed and converted from the phylogenetic tree in Fig. 2. Abbreviations in the circles of tree tips represent N: Norongarragalinales, T: Tudiarchaeales, Z: Zaosheniarchaeales, W: Wunengiarchaeales associated lineages, A: Anstonellales, R: Redboyarchaeum, M: Micrarchaeales. The dashed lines indicate the lineages where the CRISPR-Cas systems were recovered from.

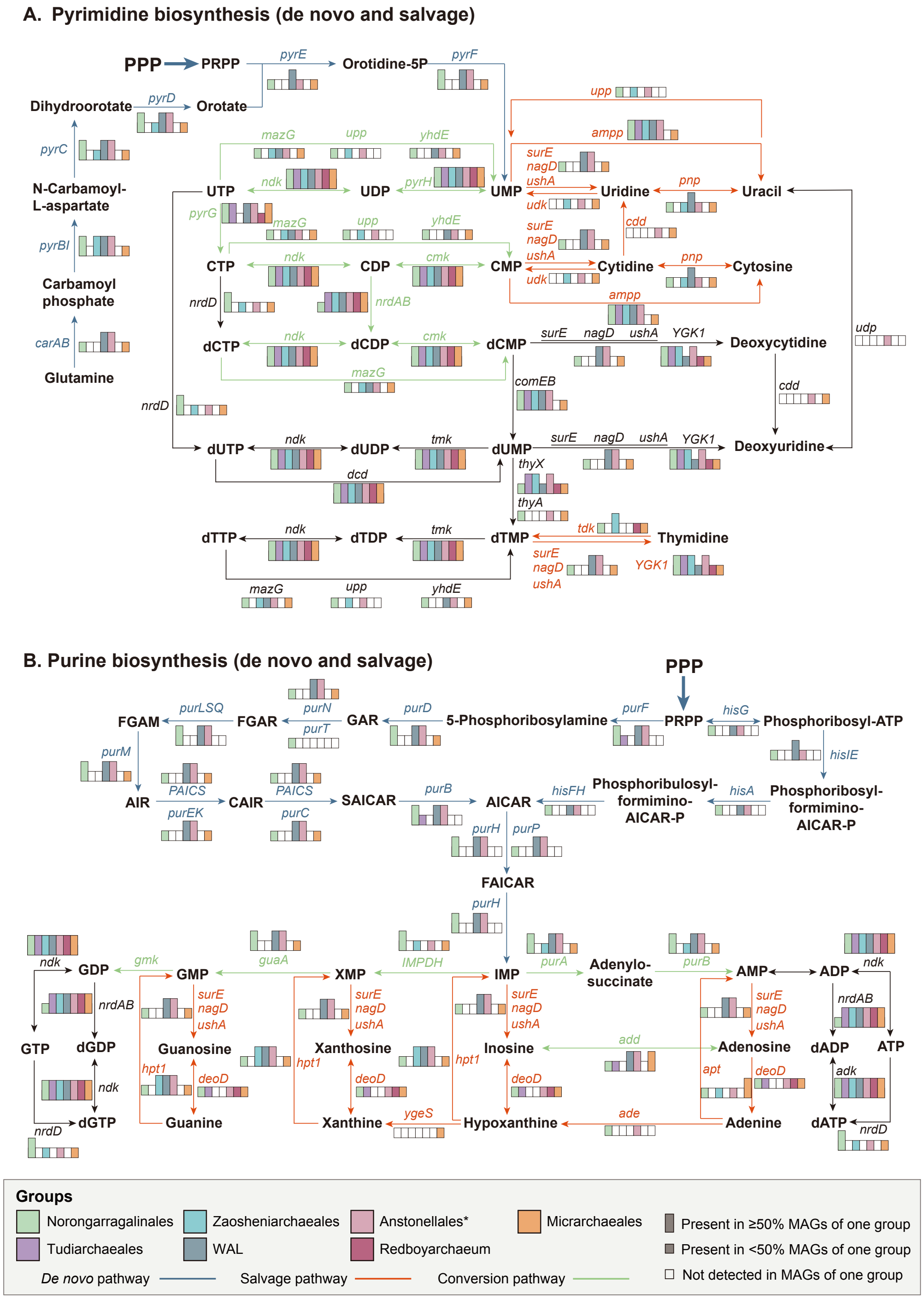

Fig. S8. Purine and pyrimidine biosynthesis pathway in Micrarchaeota. Details of gene copies in each genome are shown in Dataset S7. Abbreviations: PRPP, phosphoribosyl diphosphate; GAR, 5'-Phosphoribosylglycinamide; FGAR, 5'- Phosphoribosyl-N-formylglycinamide; FGAM, 5'-Phosphoribosyl-N-formylglycinamidine; AIR, 5'- Phosphoribosyl-5-aminoimidazole; CAIR, 5'-Phosphoribosyl-5-amino-4-imidazolecarboxylate; SAICAR, 1-(5'- Phosphoribosyl)-5-amino-4-(N-succinocarboxamide)-imidazole; AICAR, 1-(5'-Phosphoribosyl)-5-amino-4- imidazolecarboxamide; FAICAR, 1-(5'-Phosphoribosyl)-5-formamido-4-imidazolecarboxamide; WAL, Wunengiarchaeales associated lineages (WAL).

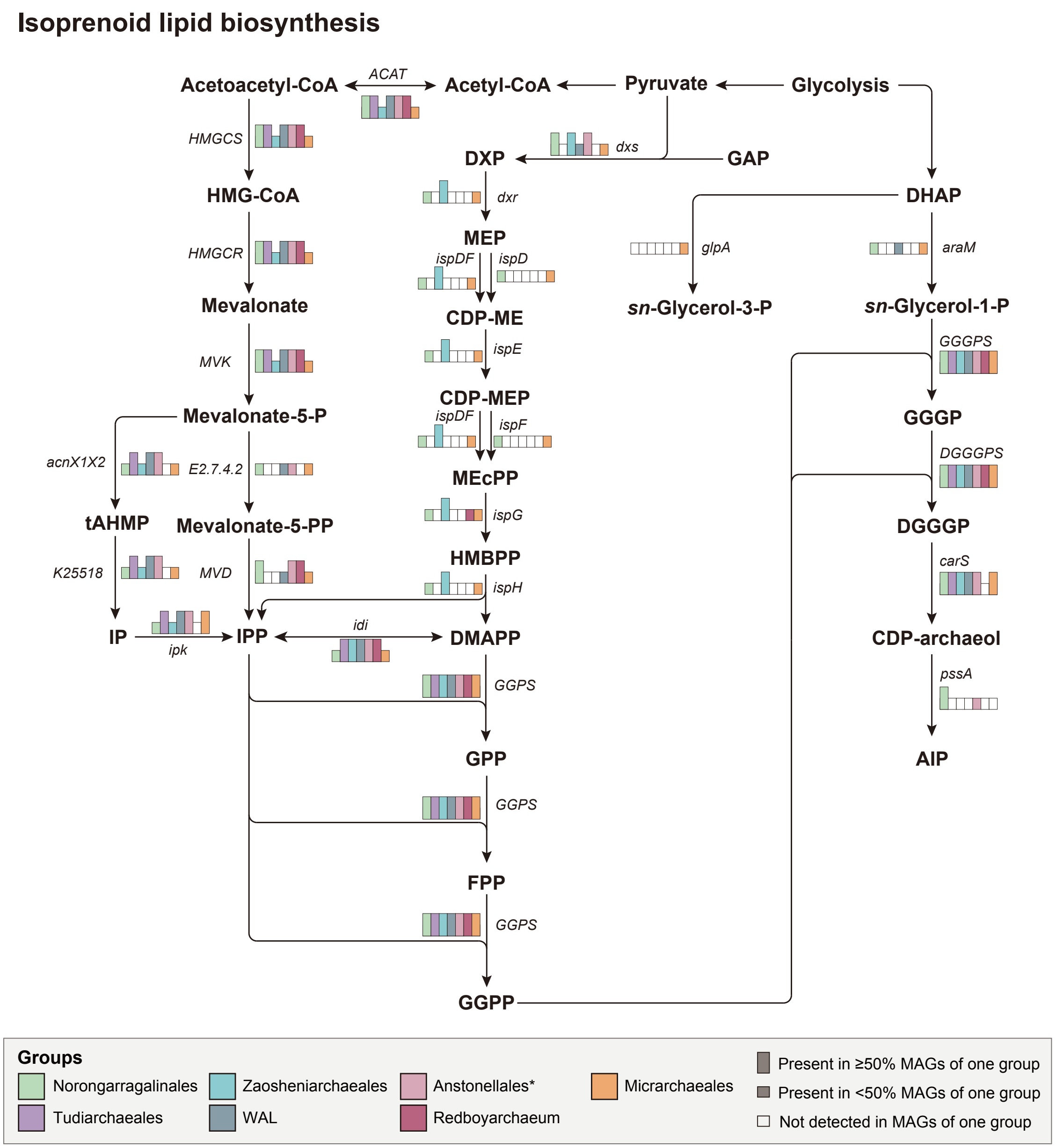

Fig. S9. Archaeal membrane lipid biosynthesis pathway in Micrarchaeota. Details of gene copies in each genome are shown in Dataset S7. Abbreviations: DHAP, Dihydroxyacetone phosphate; sn-Glycerol-1-P, sn-glycerol-1-phosphate; sn-Glycerol-3-P, sn-glycerol-3-phosphate; GGGP, geranylgeranylglyceryl phosphate; DGGGP, digeranylgeranylglyceryl phosphate; CDP-archaeol, cytidine-diphosphate-archaeol; AIP, archaetidylinositol phosphate; HMG-CoA, 3-hydroxy-3- methylglutaryl coenzyme A; Mevalonate-5-P, 5-Phosphomevalonate; Mevalonate-5-PP, 5- Diphosphomevalonate; IPP, isopentenyl diphosphate; tAHMP, trans-anhydromevalonate phosphate; IP, isopentenyl phosphate; DMAPP, dimethylallyl diphosphate; GPP, geranyl diphosphate; FPP, farnesyl diphosphate; GGPP, geranylgeranyl diphosphate; GAP, glyceraldehyde-3-phosphate; DXP, 1-Deoxy-D-Xylulose 5-Phosphate; MEP, methylerythritol 4-phosphate; CDP-ME, 4-diphosphocytidyl-2-C-methyl d-erythritol; CDP-MEP, 4-diphosphocytidyl-2C-methyl D-erythritol 2-phosphate; MEcPP, Methylerythritol cyclodiphosphate; HMBPP, hydroxylmethylbutenyl diphosphate; WAL, Wunengiarchaeales associated lineages (WAL).

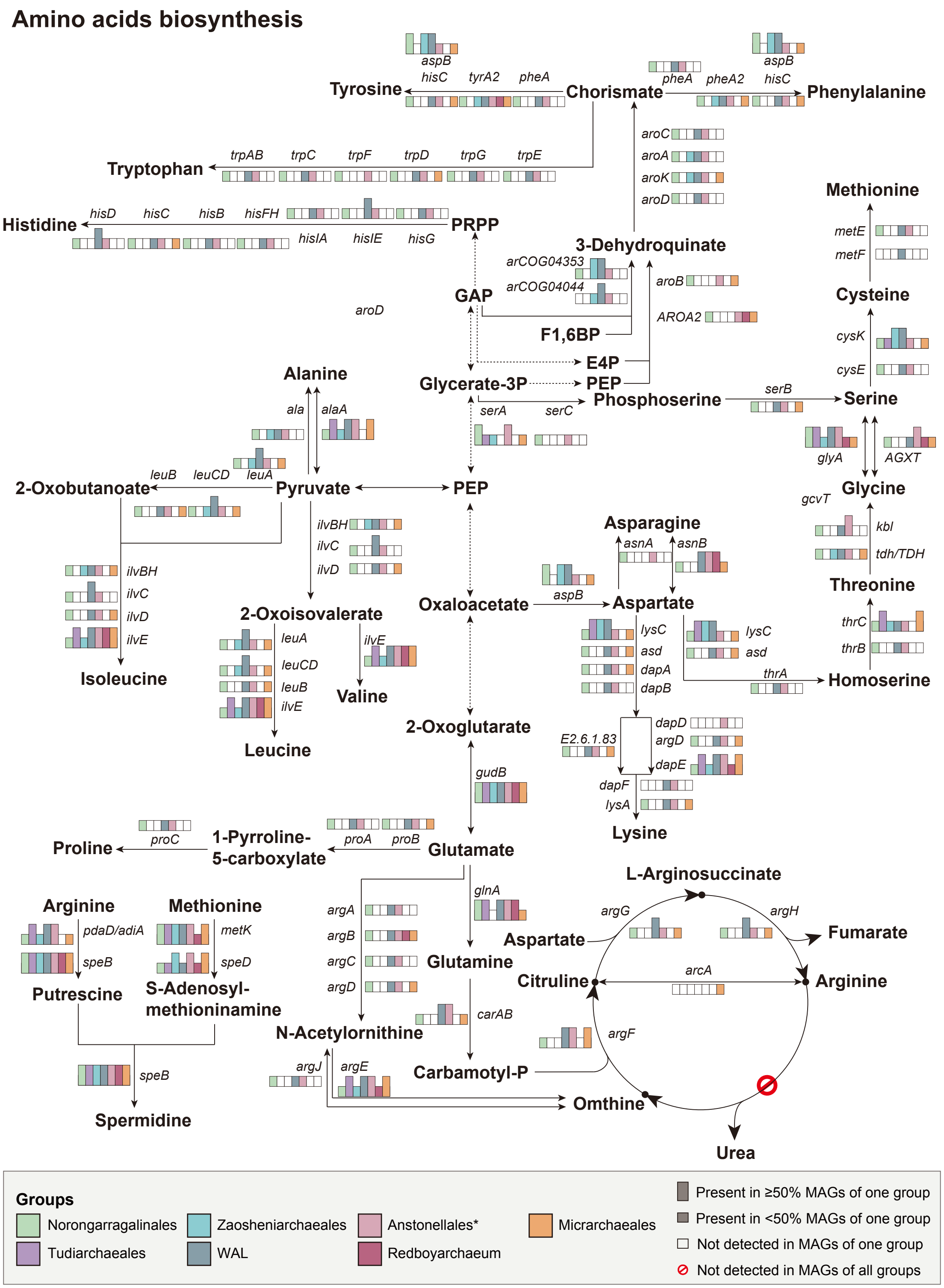

Fig. S10. Amino acid biosynthesis pathway in Micrarchaeota. Details of gene copies in each genome are shown in Dataset S7. Abbreviations: PRPP, phosphoribosyl diphosphate; GAP, glyceraldehyde-3-phosphate; E4P, D-erythrose 4- phosphate; PEP, phosphoenolpyruvate; Glycerate-3P, glycerate-3-phosphate; WAL, Wunengiarchaeales associated lineages (WAL).

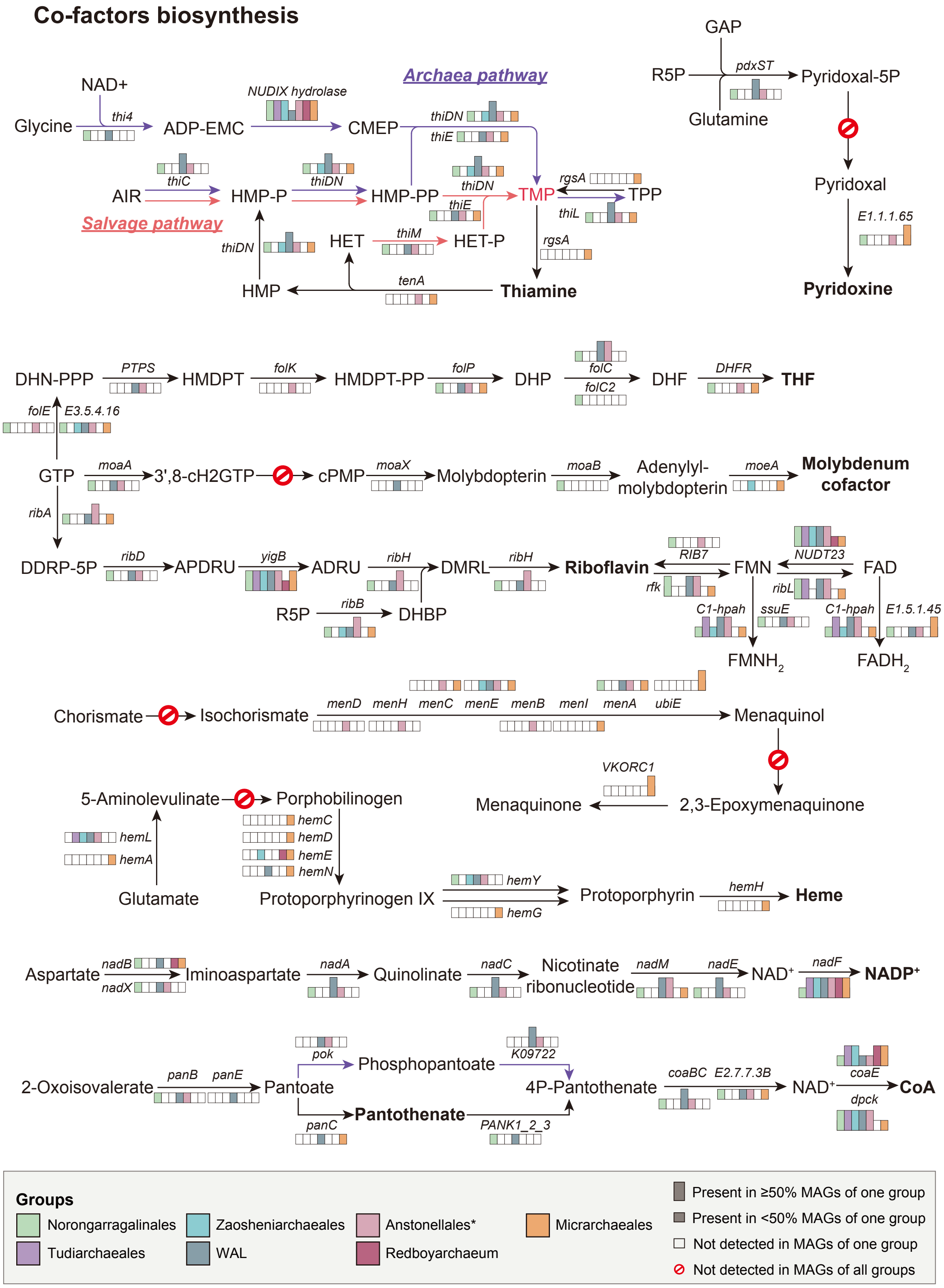

Fig. S11. Co-factors biosynthesis pathway in Micrarchaeota. Details of gene copies in each genome are shown in Dataset S7. Abbreviations: GAP, glyceraldehyde-3-phosphate; DXP, 1-Deoxy-D-Xylulose 5-Phosphate; CYMEP, 2-[(2R,5Z)-2-Carboxy-4-methylthiazol-5(2H)-ylidene]ethyl phosphate; ADP-MEC,

ADP-5-ethyl-4-methylthiazole-2-carboxylate; CMEP, 2-(2-Carboxy-4-methylthiazol-5-yl)ethyl phosphate; TMP, Thiamine phosphate; TPP, Thiamine diphosphate; AIR, Aminoimidazole ribotide; HMP-P, 4-Amino-2-methyl-5-(phosphooxymethyl)pyrimidine; HMP-PP, 4-Amino-2-methyl-5-(diphosphooxymethyl)pyrimidine; HMP, 4-Amino-5-hydroxymethyl-2-methylpyrimidine; HET, 4-Methyl-5-(2-hydroxyethyl)thiazole; HET-P, 4-Methyl-5-(2-phosphonooxyethyl)thiazole; DHN-PPP, Formamidopyrimidine nucleoside triphosphate; HMDPT, 6-(Hydroxymethyl)-7,8-dihydropterin; HMDPT-PP, 6-Hydroxymethyl-7,8-dihydropterin diphosphate; DHP, Dihydropteroate; DHF, Dihydrofolate; THF, Tetrahydrofolate; DDRP-5R, 2,5-Diamino-6-(5-phospho-D-ribosylamino)pyrimidin-4(3H)-one; APDRU, 5-Amino-6-(5-phospho-D-ribitylamino)uracil; ADRU, 5-Amino-6-(D-ribitylamino)uracil; DMRL, 6,7-Dimethyl-8-(D-ribityl)lumazine; R5P, D-Ribulose 5-phosphate; DHBP, L-3,4-Dihydroxybutan-2-one 4-phosphate; FMN, Flavin mononucleotide; FAD, Flavin adenine dinucleotide; Pyridoxine-5P, Pyridoxine 5-phosphate; WAL, Wunengiarchaeales associated lineages.

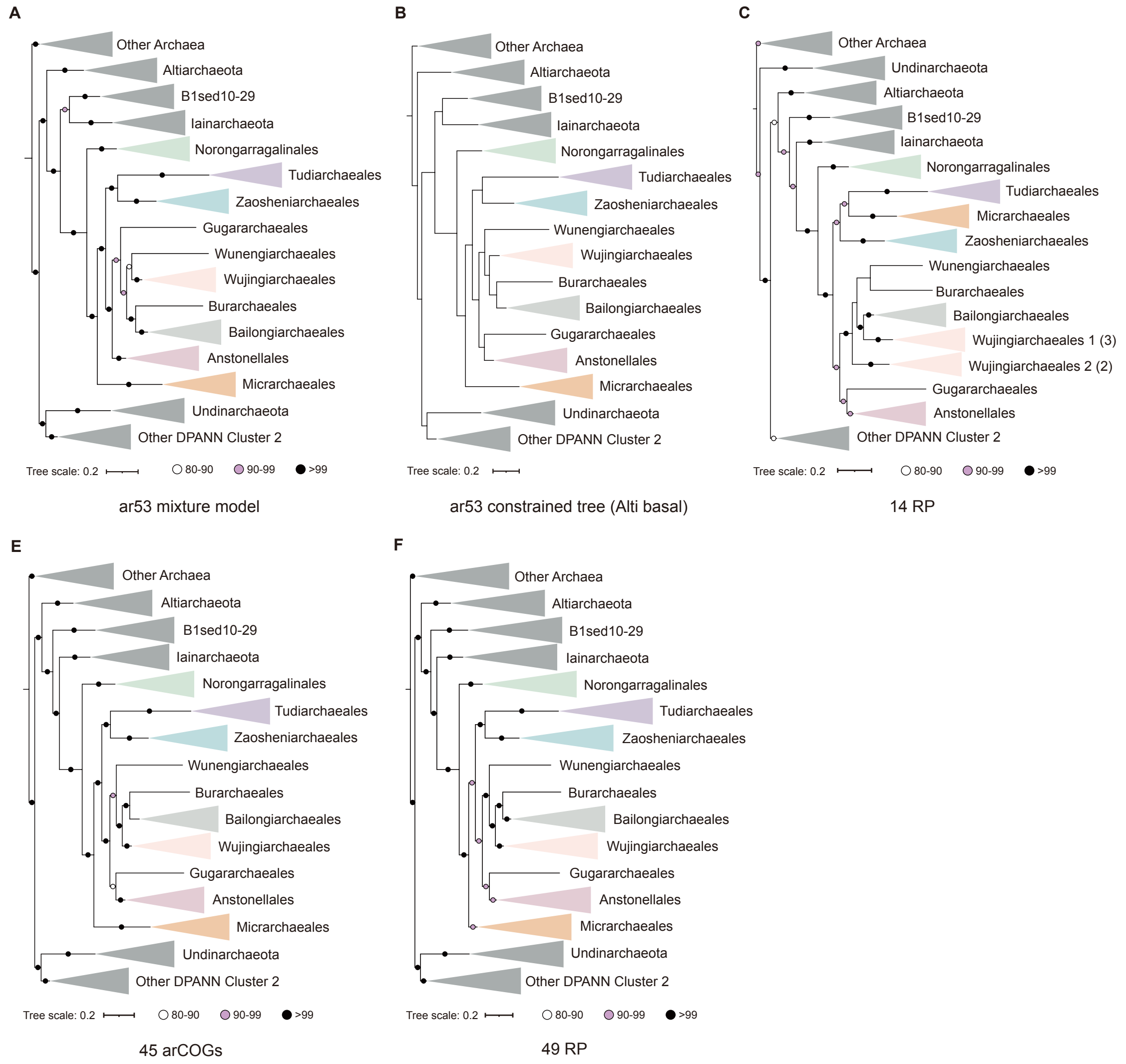

Fig. S12. Phylogenetic placement of Micrarchaeota based on different marker sets using the same genome set (494 of 658 genomes with completeness≥90% and contamination<5%) in Fig. 4 (See methods for details of different marker sets). (A) The maximum phylogeny based on 53 GTDB archaeal marker proteins with site-heterogeneous models. (B) Constrained tree that arbitrarily placed Altiarchaeota on the base of all DPANN using MSA of GTDB ar53 markers. (C) The maximum phylogeny based on 14 ribosomal proteins. (D) The maximum phylogeny based on 45 arCOGs. (E) The maximum phylogeny based on 49 ribosomal proteins. The hollow, purple, and solid circles represent the bootstrap values of 80-89%, 90-99%, and >99% respectively.

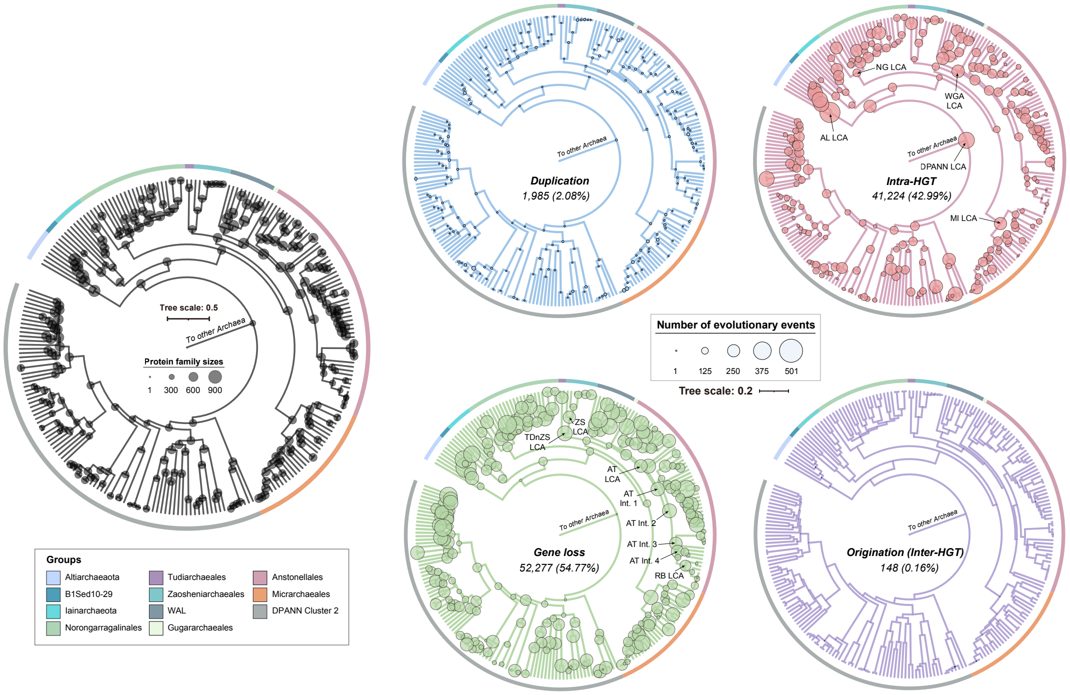

Fig. S13. The mapping of proteome sizes and evolutionary events, including duplication, intra-HGT, gene loss, and origination (inter-HGT) in each Micrarchaeota ancestral node on the same phylogenetic tree of Fig. 4 (pruned to contain only DPANN genomes). Strips with different colors represent different major lineages of Micrarchaeota and other archaea lineages. Sizes of circles represent the proteome sizes or counts of the occurred evolutionary events. Detail numbers were listed in Dataset S12. WAL in abbreviation stands for Wunengiarchaeales associated lineages.

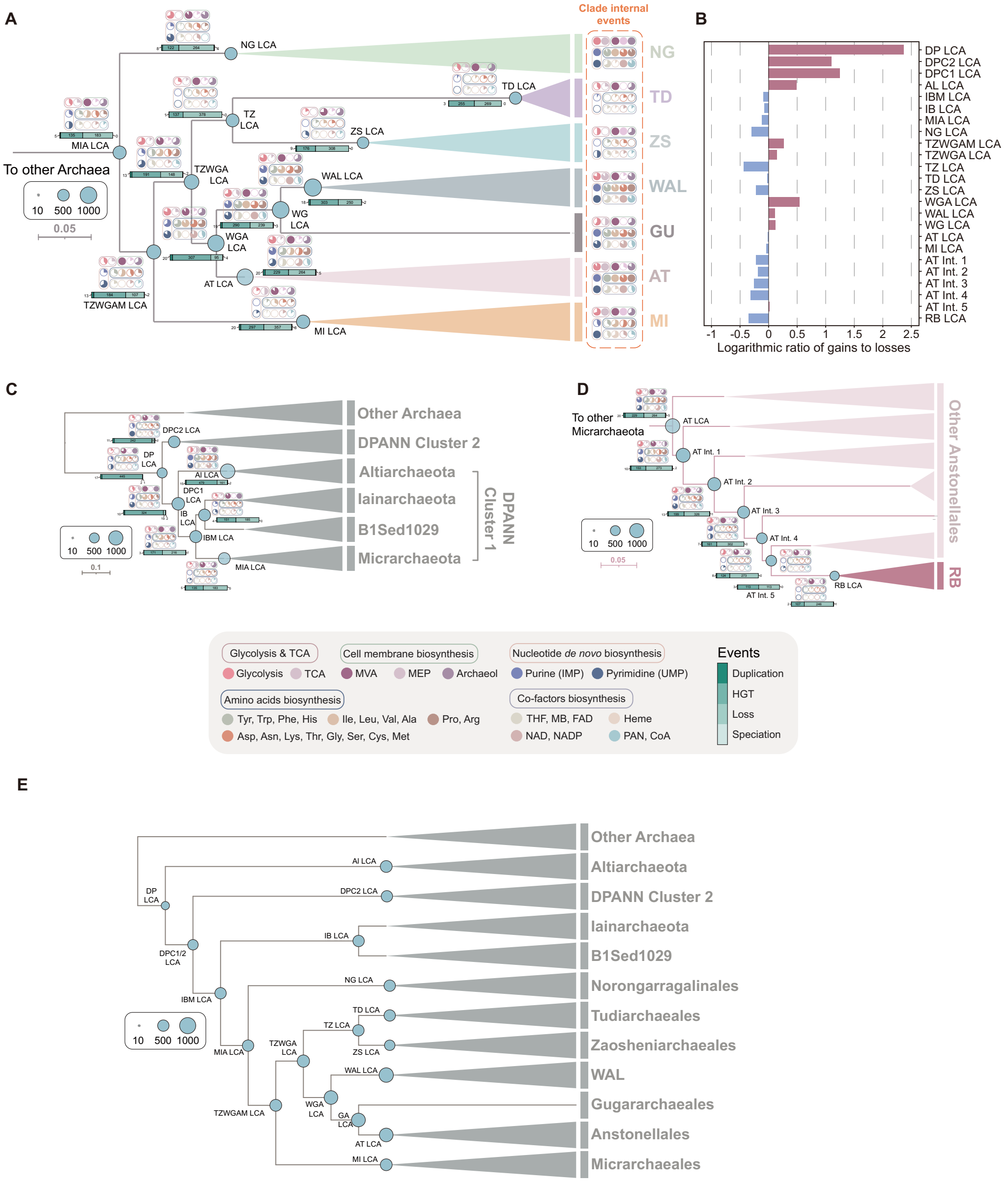

Fig. S14. Evolutionary history reconstruction of Micrarchaeota based on site-heterogeneous model tree in Fig. 14A and PMSF approximation on each individual protein family phylogeny. Panels of (A)-(D) represent the same analyses on Fig. 4 but with data based on this approach. (E) Illustration of proteome sizes in comparison with (A) and (C) but on phylogenetic tree with Altiarchaeota being arbitrarily placed on the base of all DPANN in Fig. S12B.

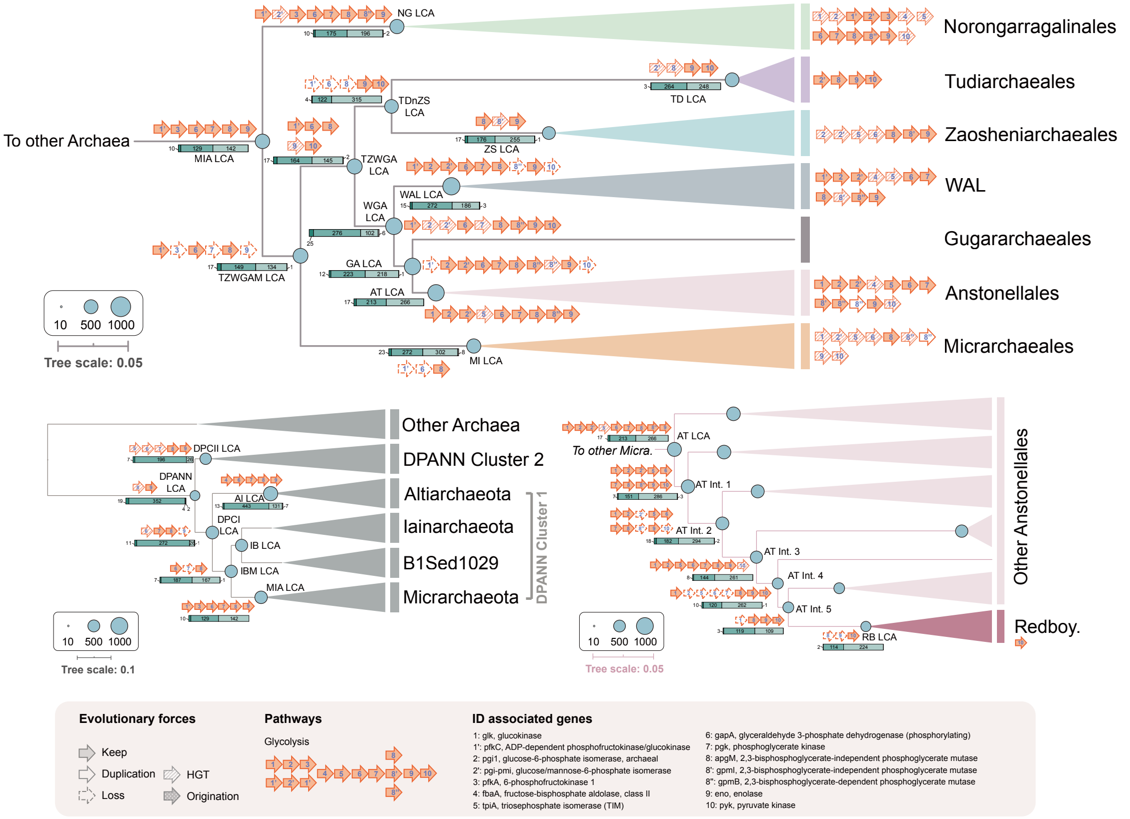

Fig. S15. Evolutionary history reconstruction of glycolysis pathway. The number within each arrow indicates the associated gene listed in Dataset S10. Different types of arrows show evolutionary events of genes, including the keep, duplication, loss, HGT, and origination in the ancestral nodes. The schematic diagram in the legend represents the glycolysis pathway (See details in Fig. 2). WAL in abbreviation stands for Wunengiarchaeales associated lineages.

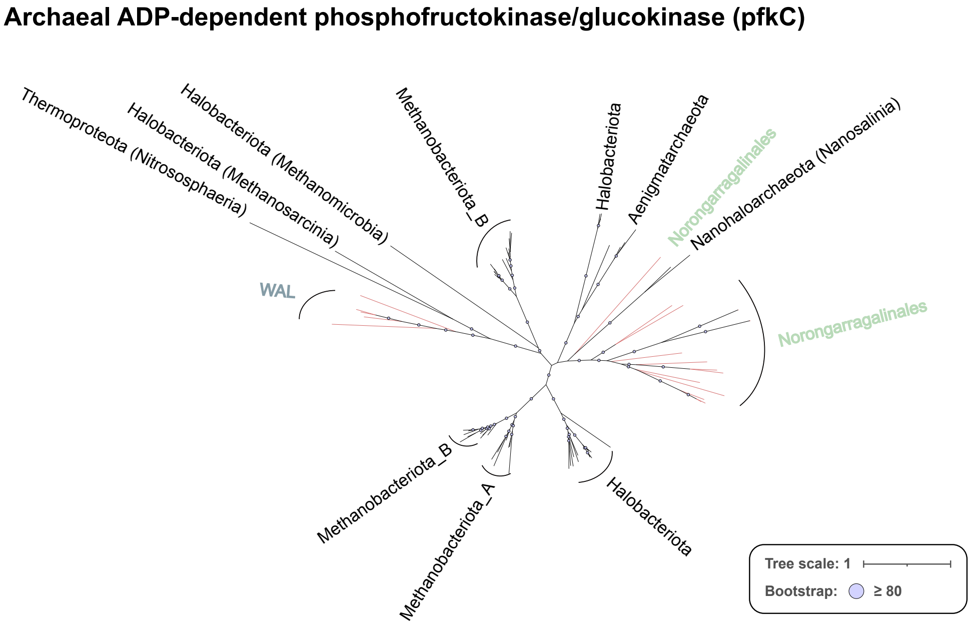

Fig. S16. Phylogenetic analysis of ADP-dependent phosphofructokinase/glucokinase (PfkC) based on posterior mean site frequency (PMSF) model. Leaves of sequences extracted from Micrarchaeota were labeled in red. WAL in abbreviation stands for Wunengiarchaeales associated lineages.

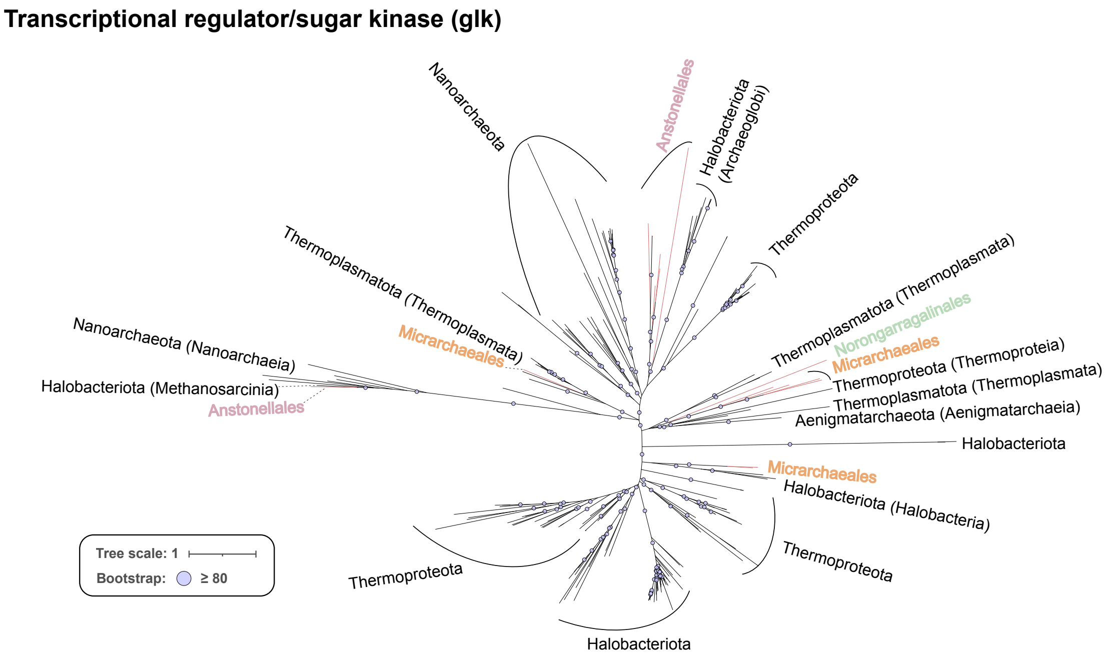

Fig. S17. Phylogenetic analysis of glucokinase (Glk) based on PMSF model. Leaves of sequences extracted from Micrarchaeota were labeled in red.

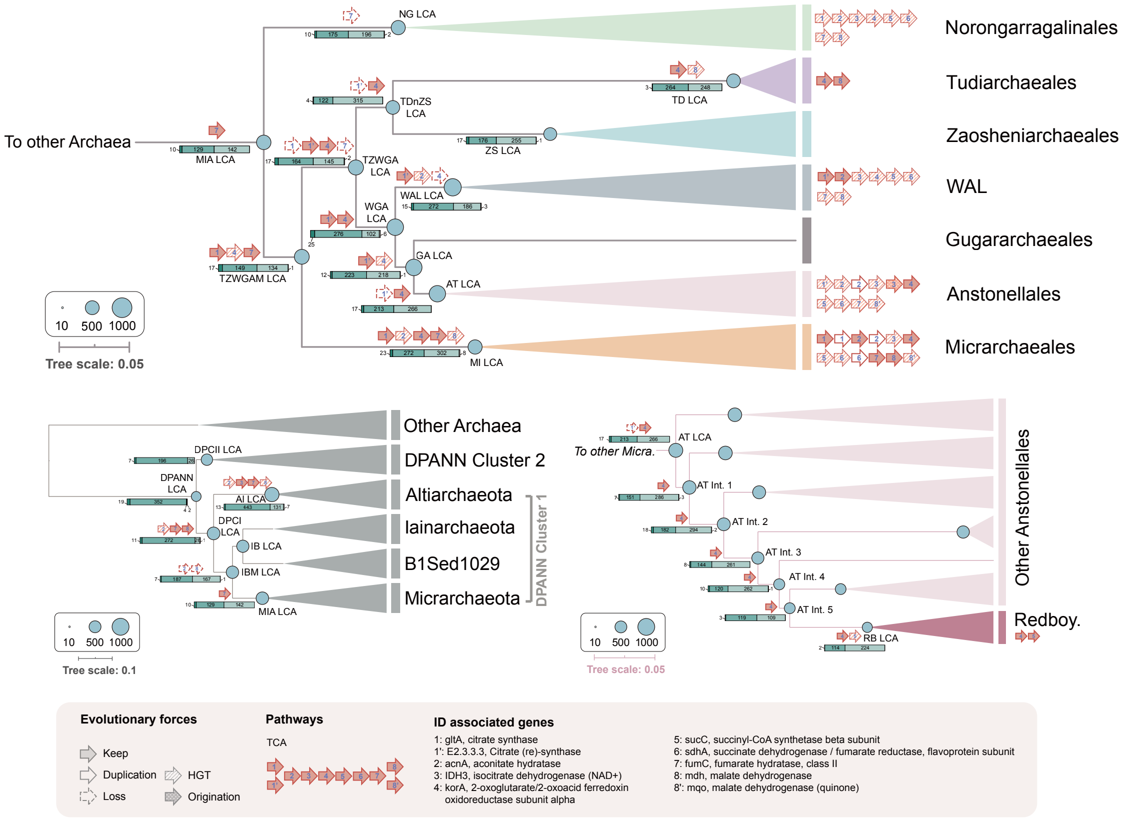

Fig. S18. Evolutionary history reconstruction of TCA cycle. The number within each arrow indicates the associated gene listed in Dataset S10. Different types of arrows show evolutionary events of genes including the keep, duplication, loss, HGT, and origination in the ancestral nodes. The schematic diagram in the legend represents the glycolysis pathway (See details in Fig. 2). WAL in abbreviation stands for Wunengiarchaeales associated lineages.

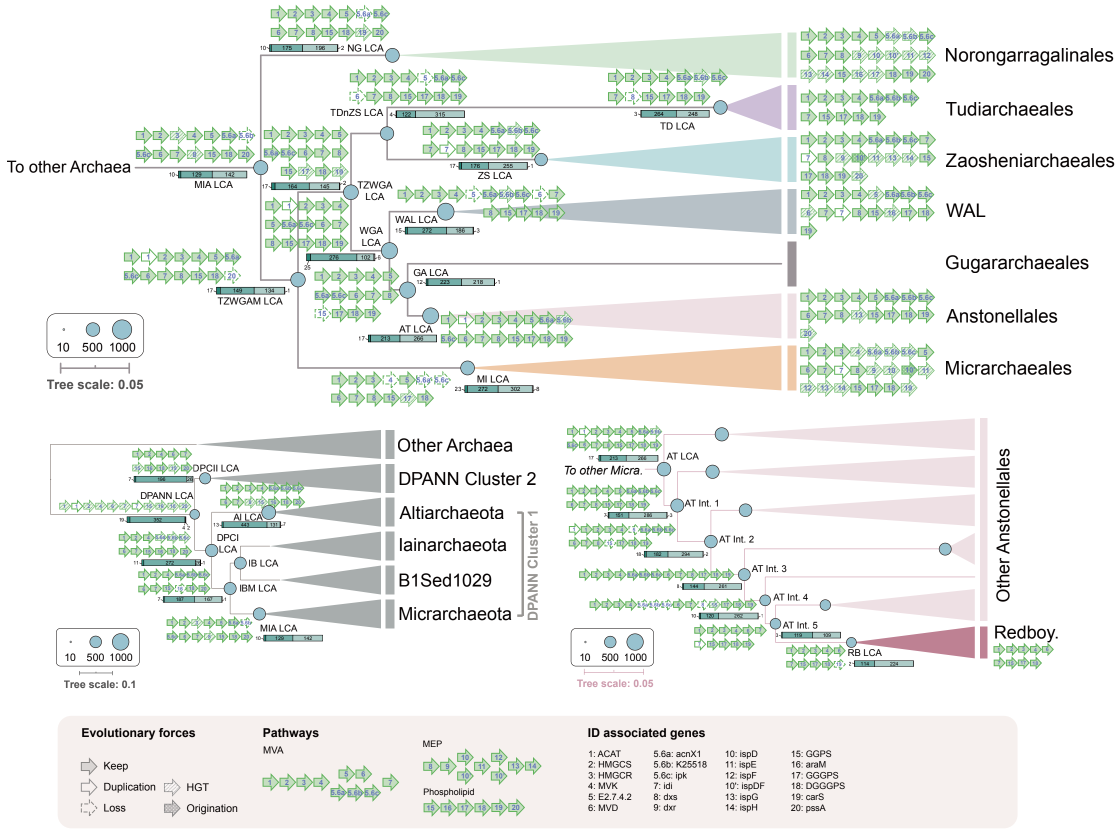

Fig. S19. Evolutionary history reconstruction of membrane biosynthesis. The number within each arrow indicates the associated gene listed in Dataset S10. Different types of arrows show evolutionary events of genes including the keep, duplication, loss, HGT, and origination in the ancestral nodes. The schematic diagram in the legend represents the membrane biosynthesis pathway (See details in Fig. S9). WAL in abbreviation stands for Wunengiarchaeales associated lineages.

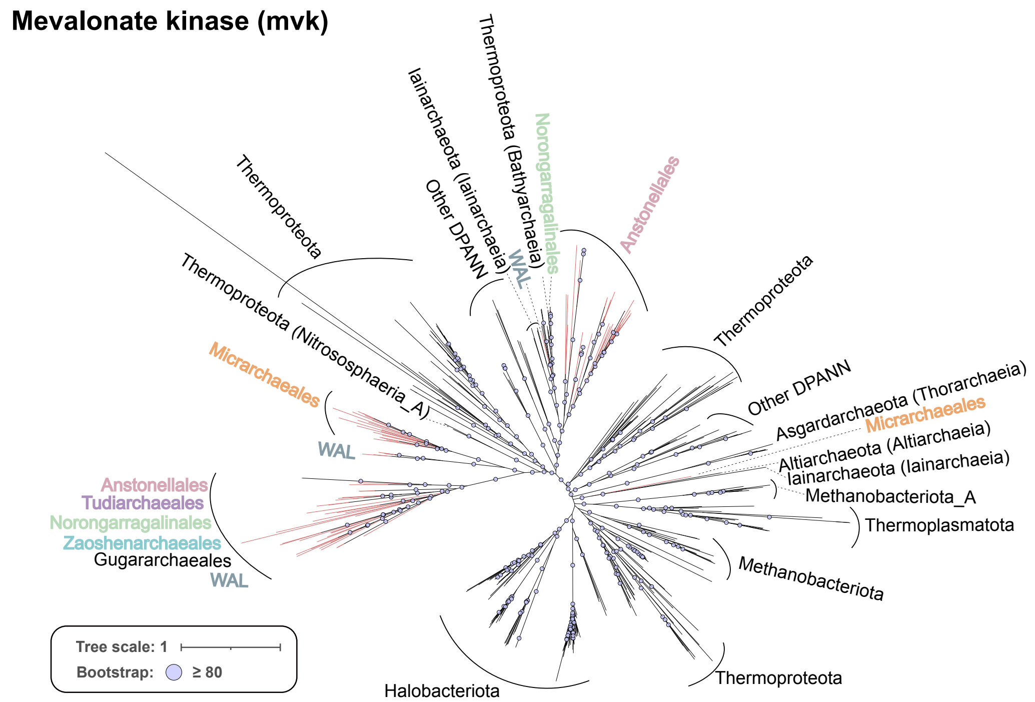

Fig. S20. Phylogenetic analysis of mevalonate kinase (Mvk) based on PMSF model. Leaves of sequences extracted from Micrarchaeota were labeled in red. WAL in abbreviation stands for Wunengiarchaeales associated lineages.

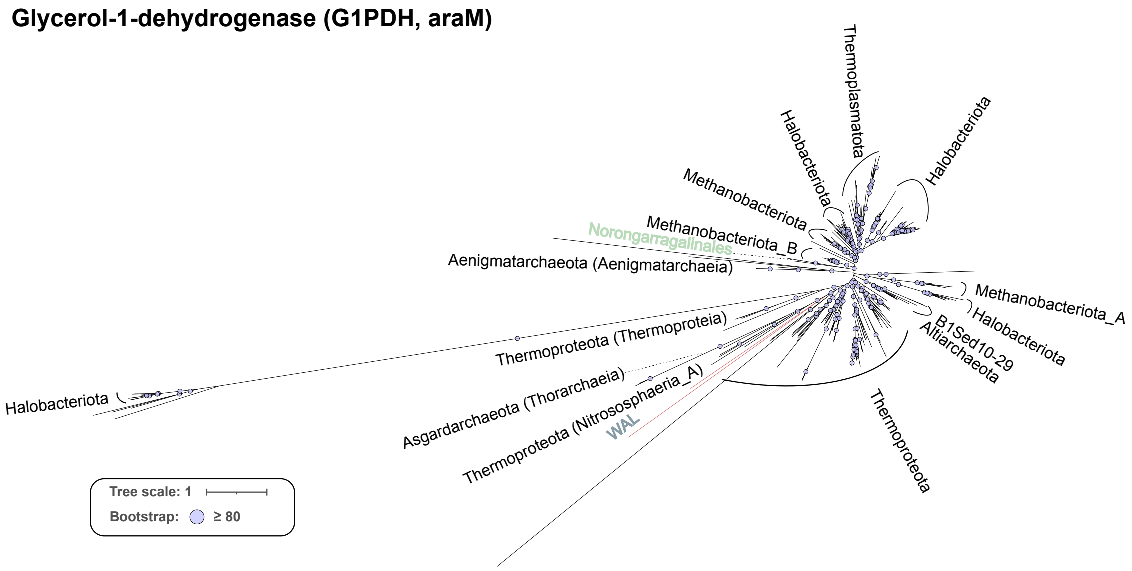

Fig. S21. Phylogenetic analysis of glycerol-1-phosphate dehydrogenase (G1PDH, AraM). The leaf of the sequence extracted from Micrarchaeota was labeled in red. WAL in abbreviation stands for Wunengiarchaeales associated lineages.

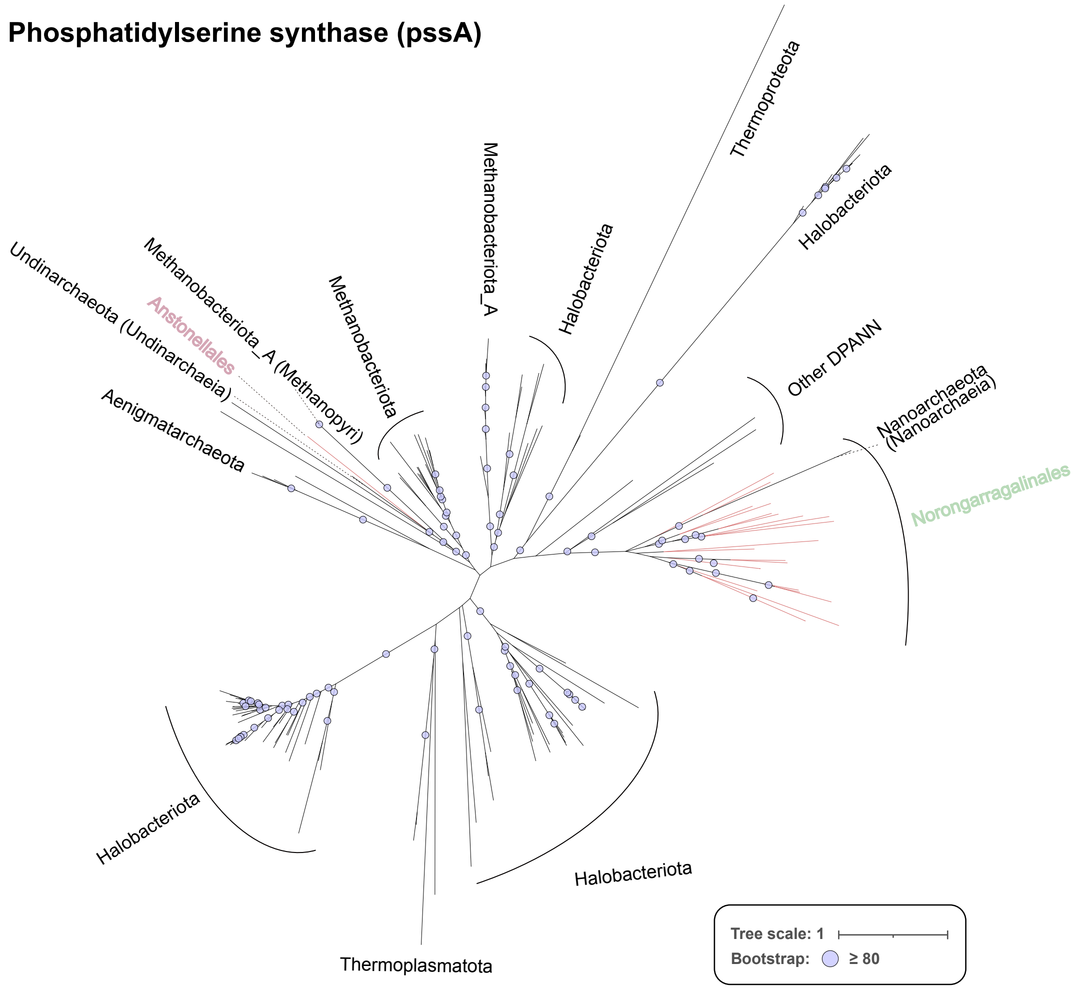

Fig. S22. Phylogenetic analysis of phosphatidylserine synthase (PssA) based on PMSF model. The leaf of the sequence extracted from Micrarchaeota was labeled in red.

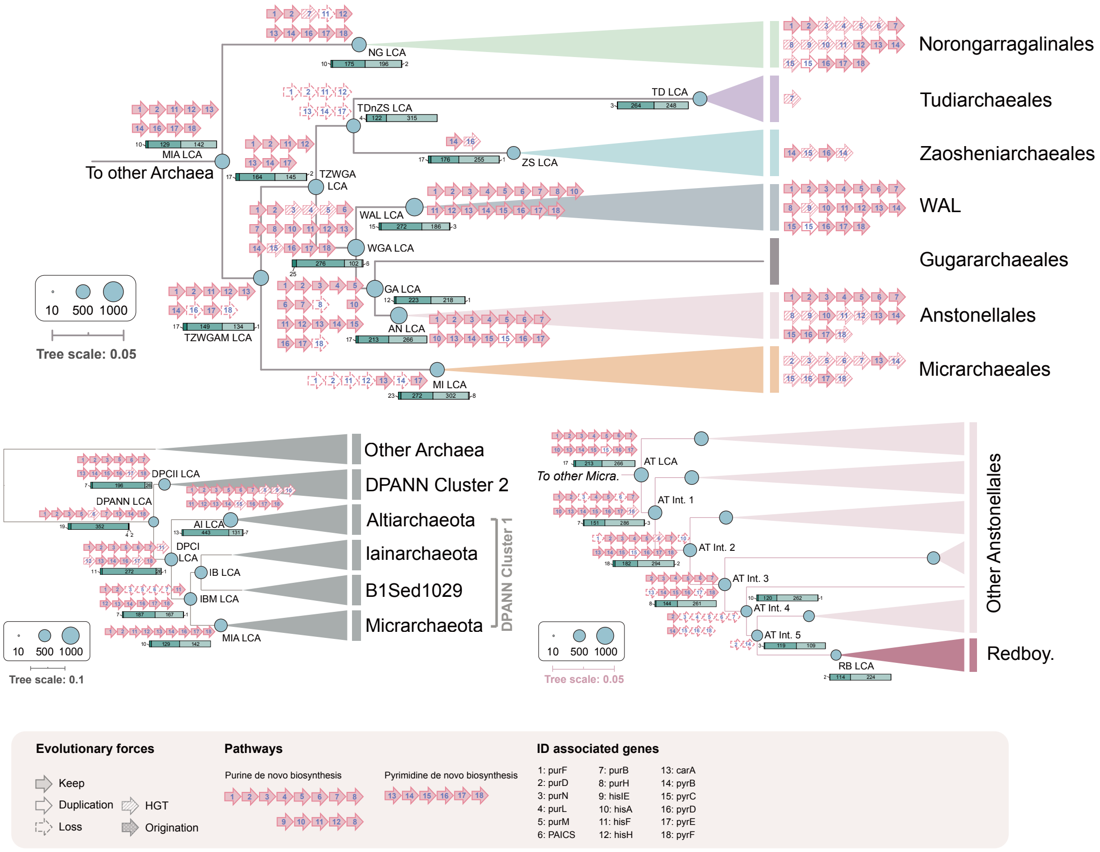

Fig. S23. Evolutionary history reconstruction of *de novo* purine and pyrimidine biosynthesis. The number within each arrow indicates the associated gene listed in Dataset S10. Different types of arrows show evolutionary events of genes including the keep, duplication, loss, HGT, and origination in the ancestral nodes. The schematic diagram in the legend represents the purine *de novo* biosynthesis pathway (See details in Fig. S8). WAL in abbreviation stands for Wunengiarchaeales associated lineages.

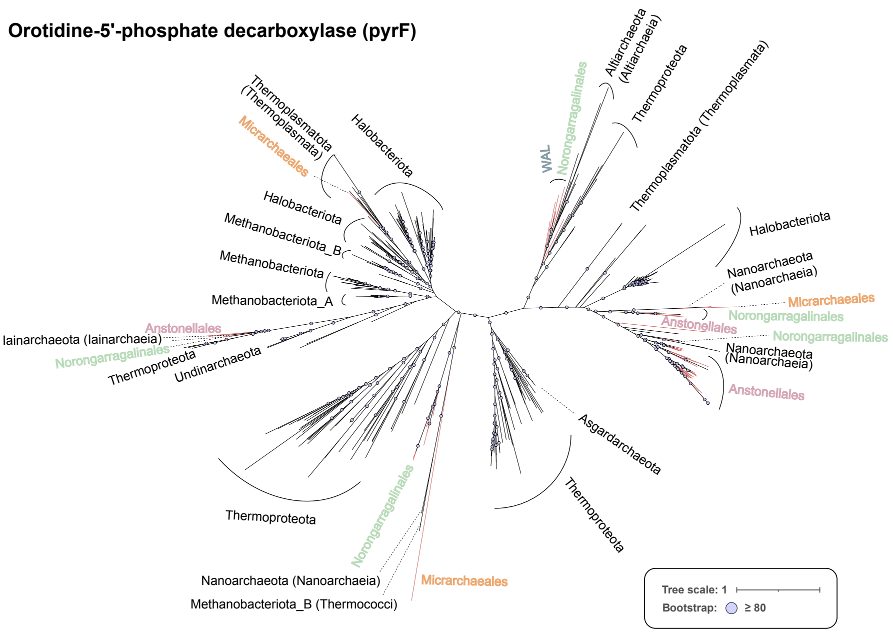

Fig. S24. Phylogenetic analysis of Orotidine-5'-phosphate decarboxylase (PyrF) based on PMSF model. The leaf of the sequence extracted from Micrarchaeota was labeled in red. WAL in abbreviation stands for Wunengiarchaeales associated lineages.

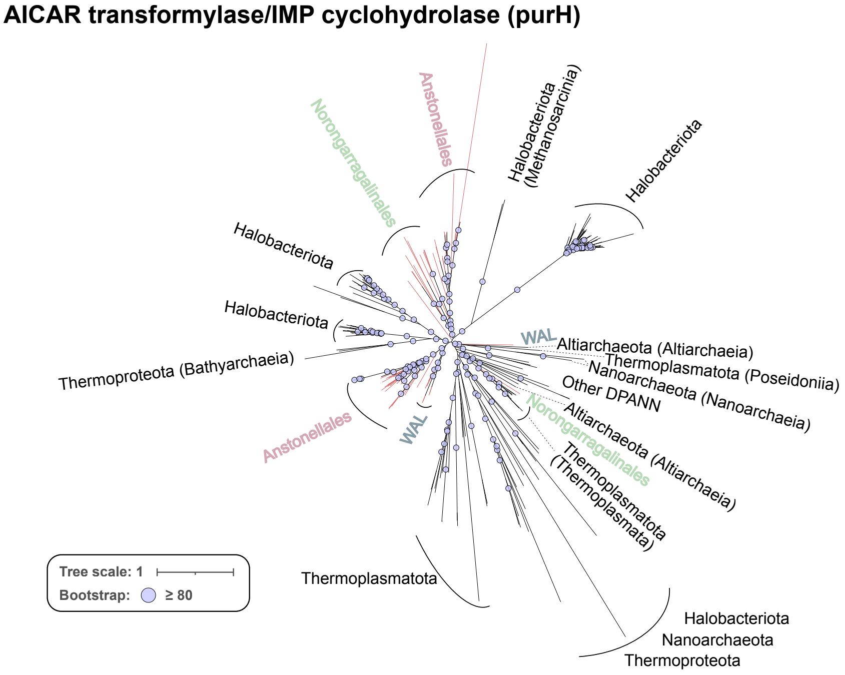

Fig. S25. Phylogenetic analysis of AICAR transformylase/IMP cyclohydrolase (PurH) based on PMSF model. The leaf of the sequence extracted from Micrarchaeota was labeled in red. WAL in abbreviation stands for Wunengiarchaeales associated lineages.

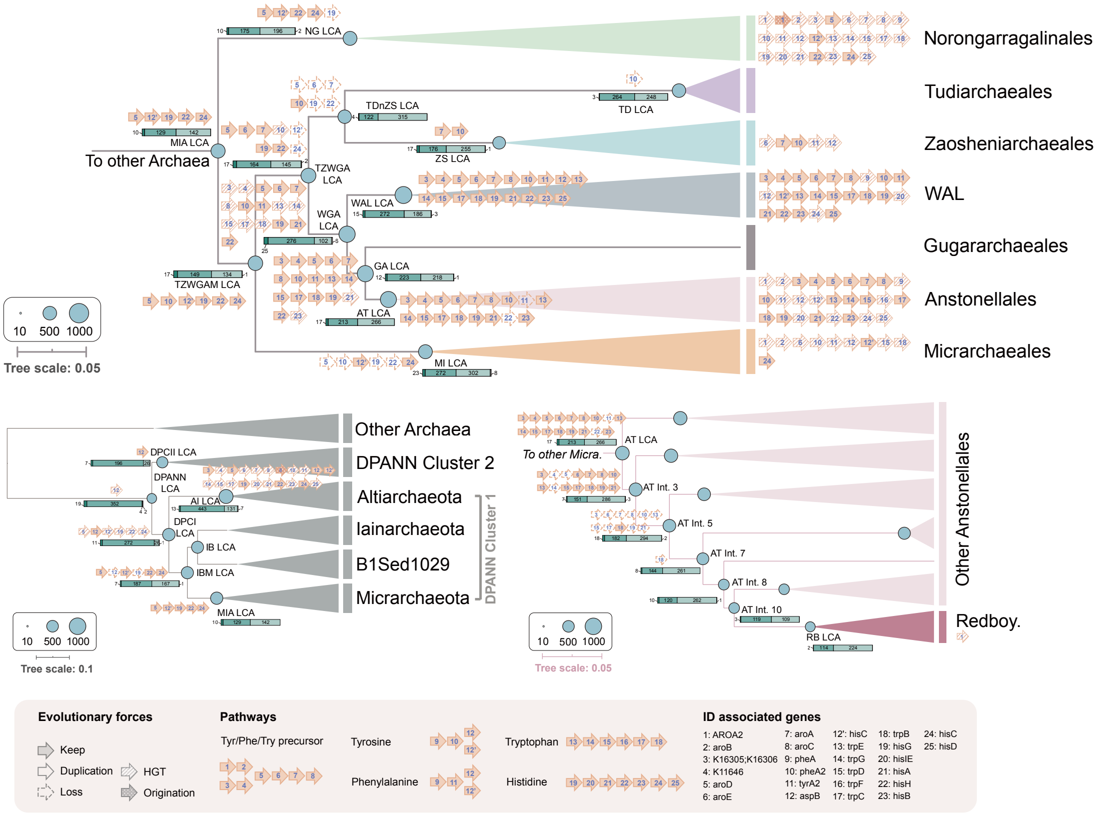

Fig. S26. Evolutionary history reconstruction of the amino acid (Tyrosine, Phenylalanine, Tryptophan, and Histidine) biosynthesis. The number within each arrow indicates the associated gene listed in Dataset S10. Different types of arrows show evolutionary events of genes including the keep, duplication, loss, HGT, and origination in the ancestral nodes. The schematic diagram in the legend represents the Tyr/Phe/Try module of the amino acid biosynthesis pathway (See details in Fig. S10). WAL in abbreviation stands for Wunengiarchaeales associated lineages.

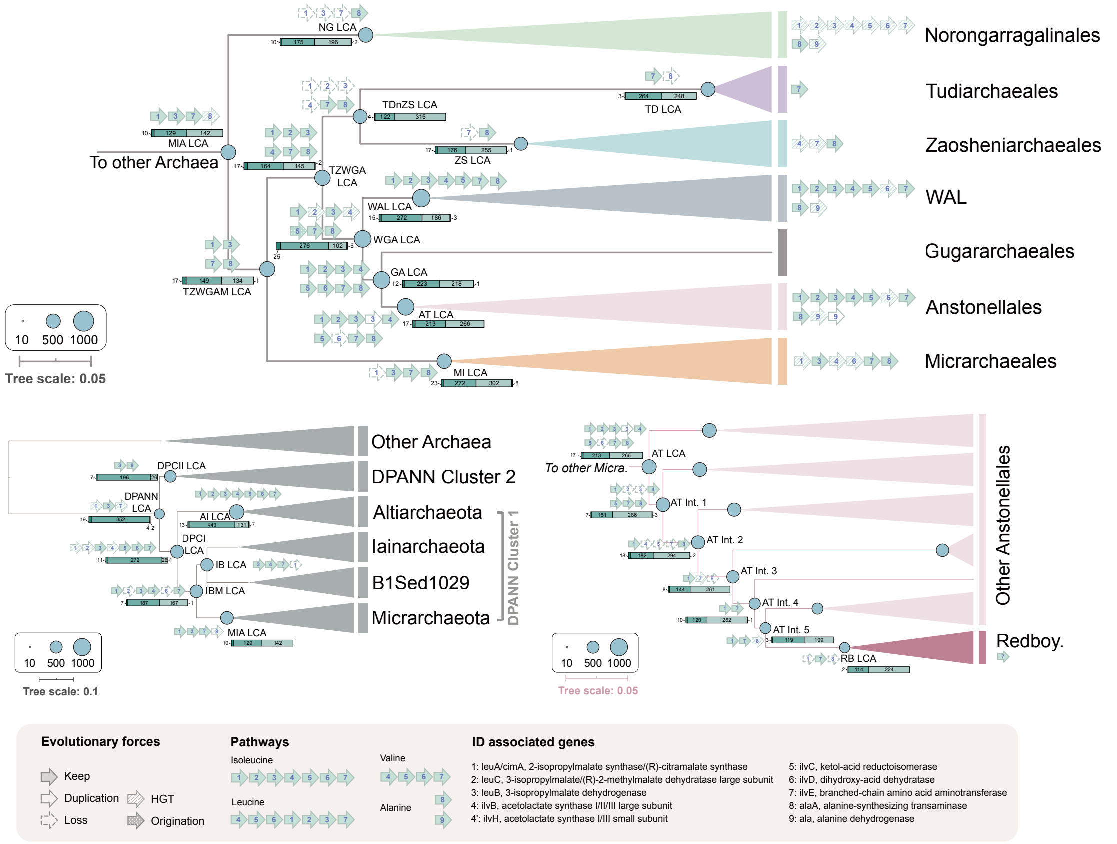

Fig. S27. Evolutionary history reconstruction of the amino acid (Isoleucine, Leucine, Valine, and Alanine) biosynthesis. The number within each arrow indicates the associated gene listed in Dataset S10. Different types of arrows show evolutionary events of genes including the keep, duplication, loss, HGT, and origination in the ancestral nodes. The schematic diagram in the legend represents the Ile/Leu/Val/Ala module of the amino acid biosynthesis pathway (See details in Fig. S10). WAL in abbreviation stands for Wunengiarchaeales associated lineages.

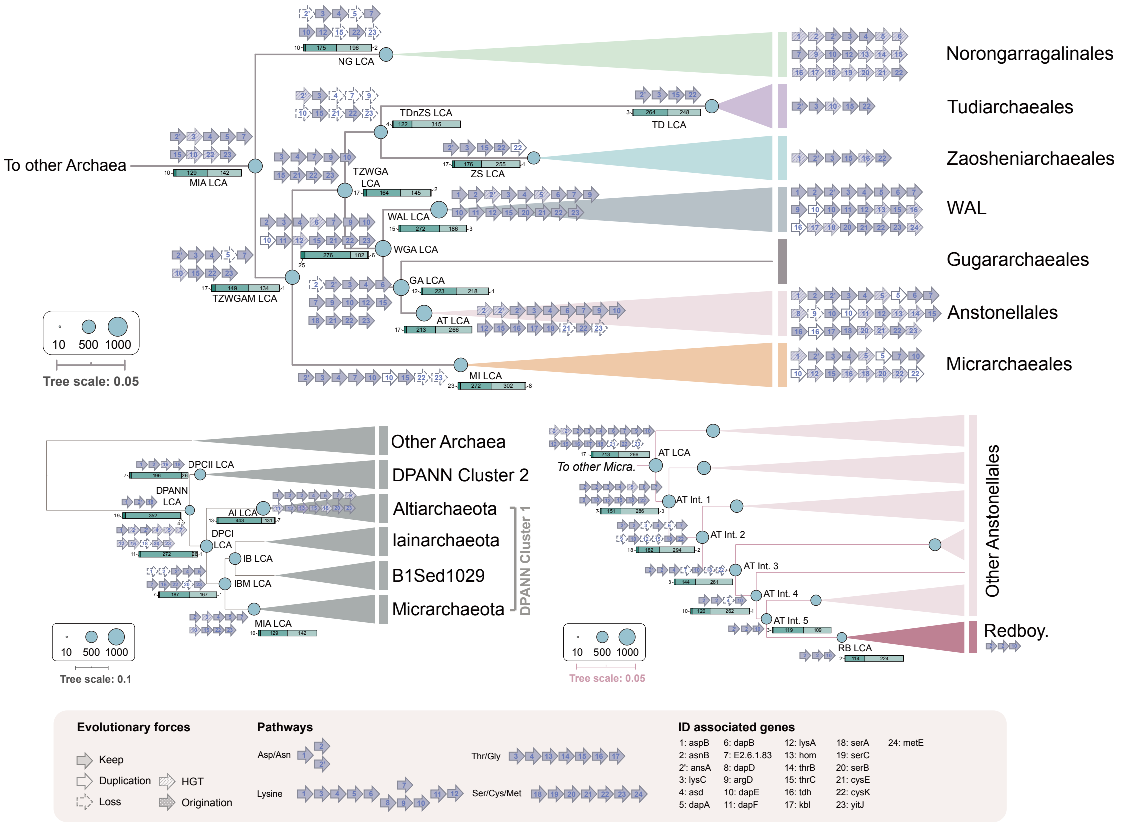

Fig. S28. Evolutionary history reconstruction of amino acid (Asparagine, Aspartate, Threonine, Glycine, Lysine, Serine, Cysteine, and Methionine) biosynthesis. The number within each arrow indicates the associated gene listed in Dataset S10. Different types of arrows show evolutionary events of genes including the keep, duplication, loss, HGT, and origination in the ancestral nodes. The schematic diagram in the legend represents the Asp/Asn/Thr/Gly/Ser/Cys/Met module of the amino acid biosynthesis pathway (See details in Fig. S10). WAL in abbreviation stands for Wunengiarchaeales associated lineages.

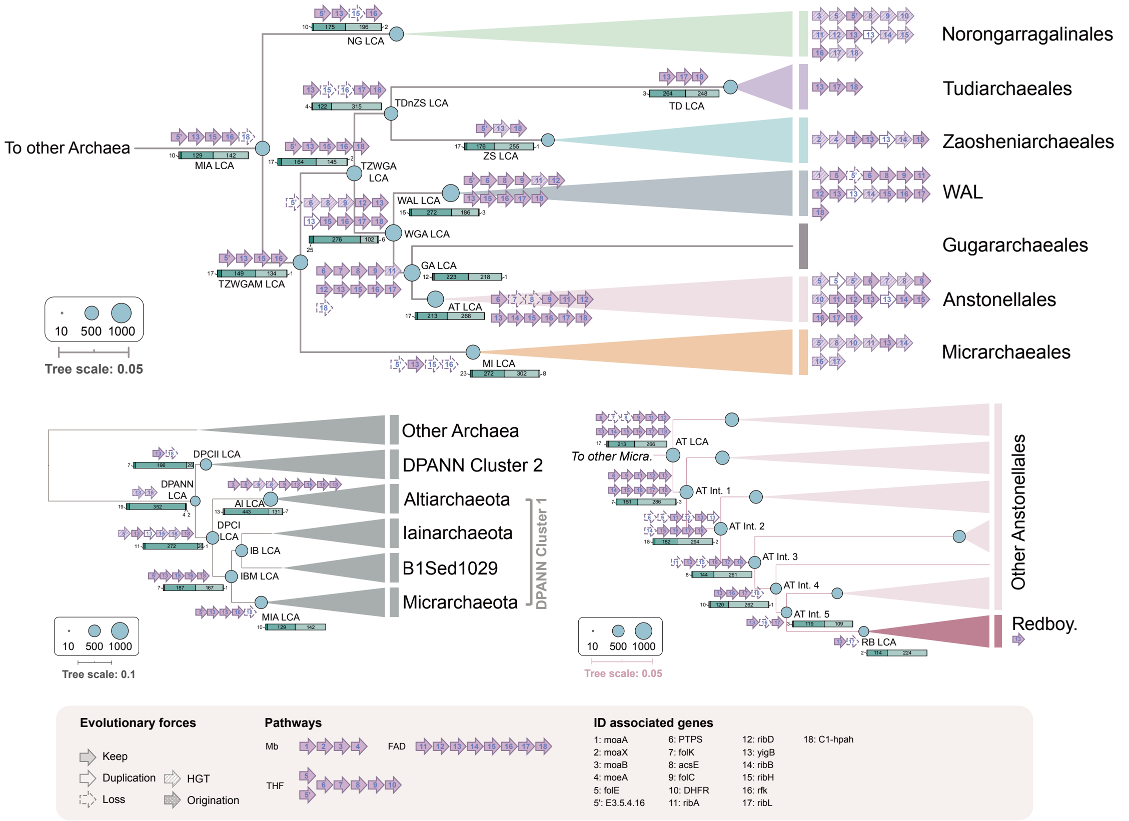

Fig. S29. Evolutionary history reconstruction of co-factors (Molybdenum, tetrahydrofolate (THF), flavin mononucleotide (FMN), flavin adenine dinucleotide (FAD)) biosynthesis. The number within each arrow indicates the associated gene listed in Dataset S10. Different types of arrows show evolutionary events of genes including the keep, duplication, loss, HGT, and origination in the ancestral nodes. The schematic diagram in the legend represents the co-factors biosynthesis pathway (See details in Fig. S11). WAL in abbreviation stands for Wunengiarchaeales associated lineages.

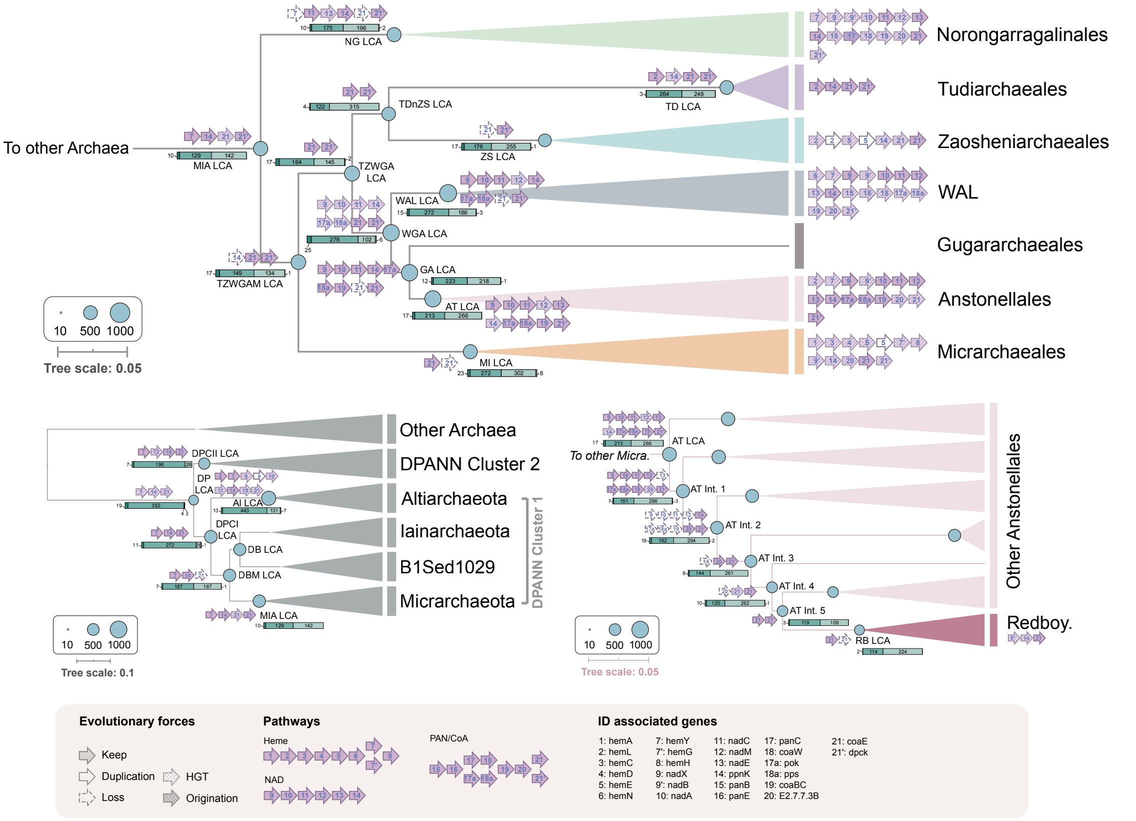

Fig. S30. Evolutionary history reconstruction of co-factors (heme, nicotinamide adenine dinucleotide phosphate ($NADP^+$/NADPH), nicotinamide adenine dinucleotide (NAD^+/NADH), and coenzyme A (CoA)) biosynthesis. The number within each arrow indicates the associated gene listed in Dataset S10. Different types of arrows show evolutionary events of genes including the keep, duplication, loss, HGT, and origination in the ancestral nodes. The schematic diagram in the legend represents the co-factors biosynthesis pathway (See details in Fig. S11). WAL in abbreviation stands for Wunengiarchaeales associated lineages.

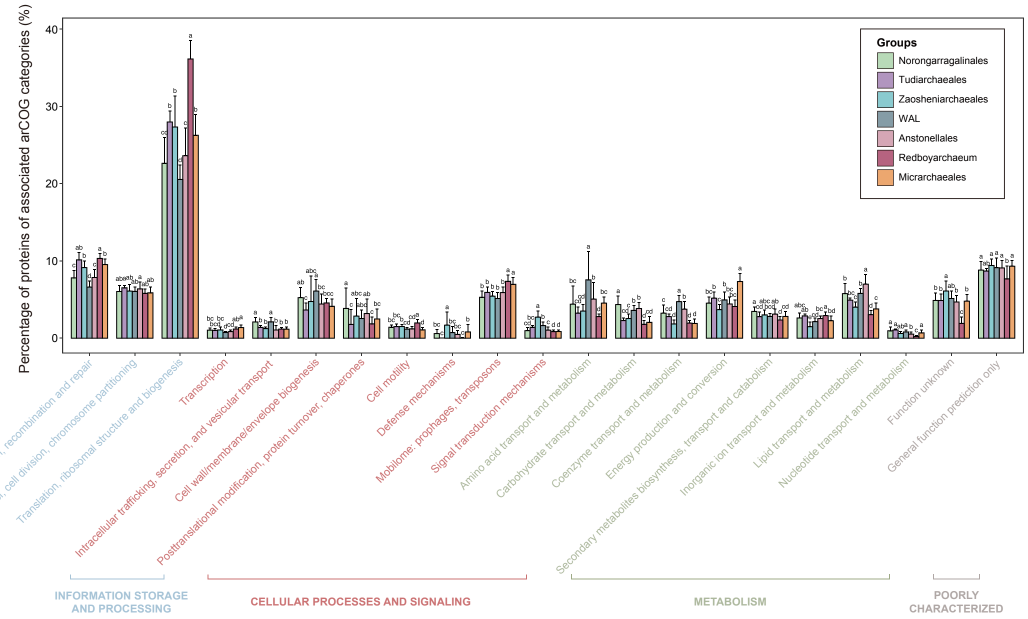

Fig. S31. Metabolic category statistics based on arCOG gene number of Micrarchaeota. Differences in major lineages of Micrarchaeota in all categories were assessed using analysis of the variance (ANOVA), followed by HSD post hoc all-pairwise comparisons test. Significant differences among groups were marked with alphabetic letters on the top of the bars. Bars without sharing the same letters (such as “a” and “b”) suggest a significant difference between them. “L”, Replication, recombination and repair; “K”, Transcription; “J”, Translation, ribosomal structure and biogenesis; “D”, Cell cycle control, cell division, chromosome partitioning; “N”, Cell motility; “M”, Cell wall/membrane/envelope biogenesis; “V”, Defense mechanisms; “U”, Intracellular trafficking, secretion, and vesicular transport; “X”, Mobilome: prophages, transposons; “O”, Posttranslational modification, protein turnover, chaperones; “T”, Signal transduction mechanisms; “E”, Amino acid transport and metabolism; “G”, Carbohydrate transport and metabolism; “H”, Coenzyme transport and metabolism; “C”, Energy production and conversion; “P”, Inorganic ion transport and metabolism; “I”, Lipid transport and metabolism; “F”, Nucleotide transport and metabolism; “Q”, Secondary metabolites biosynthesis, transport and catabolism; “S”, Function unknown; “R”, General function prediction only. WAL in abbreviation stands for Wunengiarchaeales associated lineages.

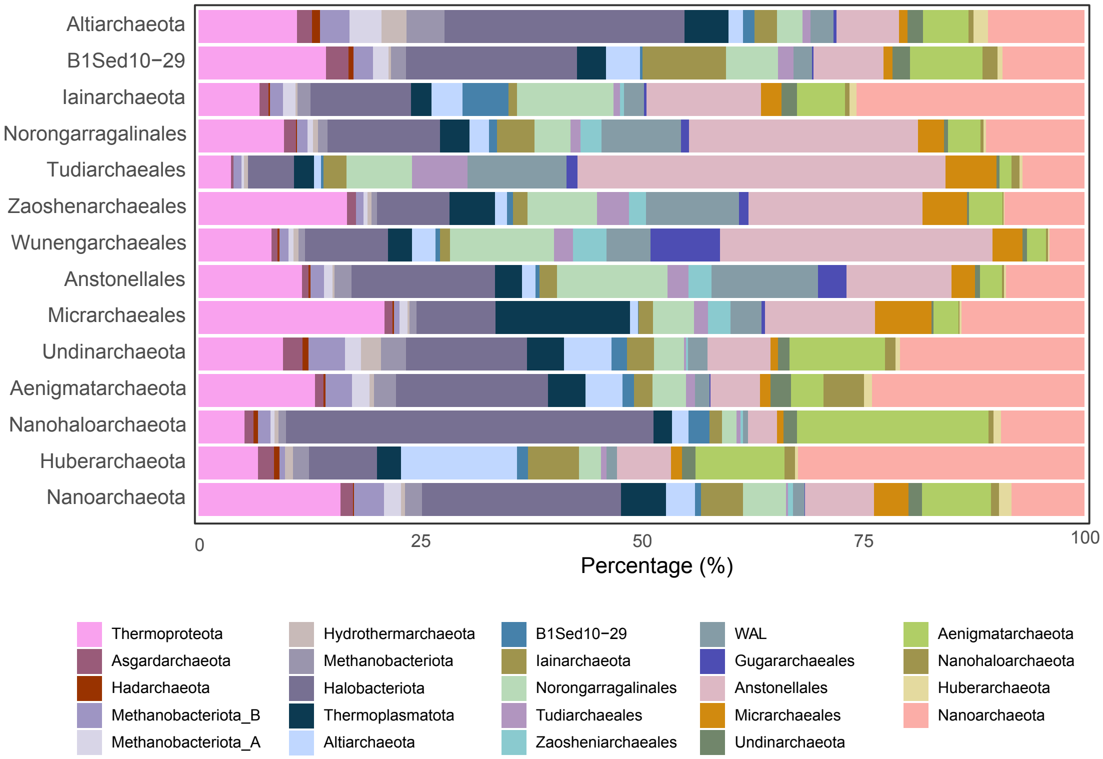

Fig. S32. Potential sources of HGTs regarding the sister group of each protein family indicate the sharing of gene ratios between major lineages of Micrarchaeota and other archaea lineages. The plot was generated based on 494 genomes and phylogeny of 8,866 derived protein families used in evolutionary analyses. Colors in stacked bar plots indicated different archaea phyla/major lineages of Micrarchaeota. WAL in abbreviation stands for Wunengiarchaeales associated lineages.

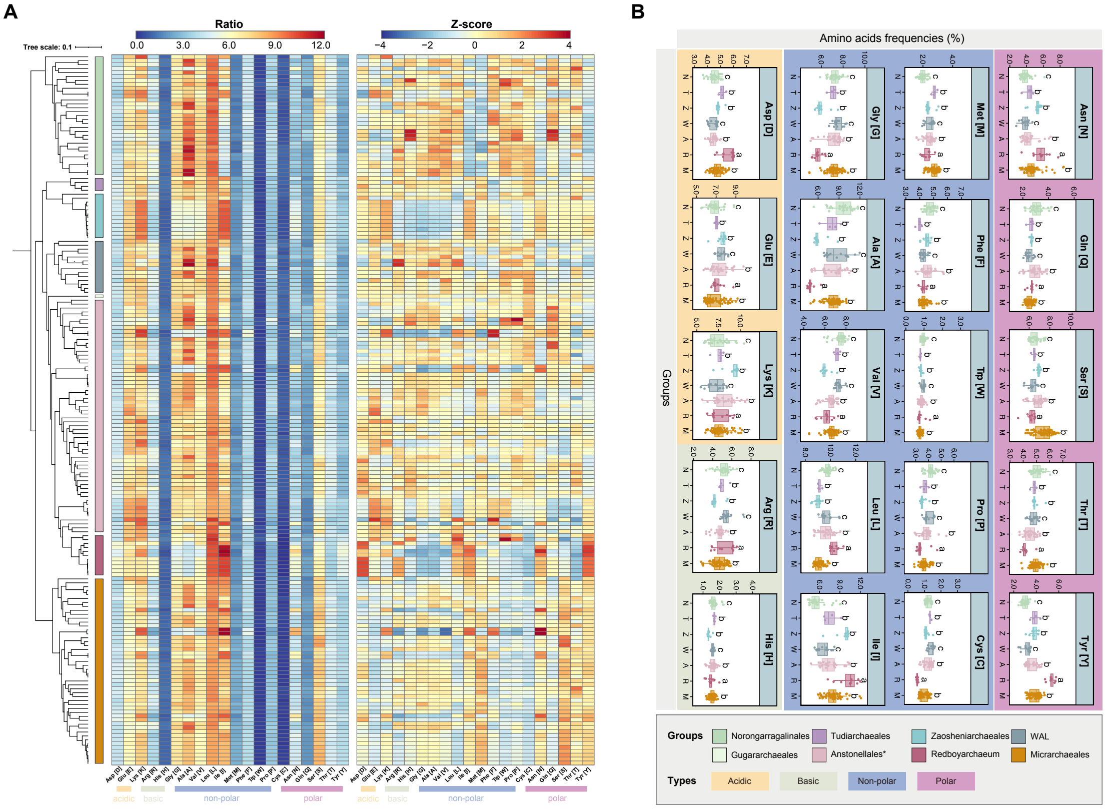

Fig. S33. Frequencies of amino acids of each genome and statistics of usage preferences of major lineages of Micrarchaeota. (A) The schematic tree was converted from the phylogenetic tree of Fig. 1B. The heatmap on the left shows the amino acid frequencies of each genome. The heatmap on the right was Z-transformed to reveal the differences in amino acid preferences between different genomes. (B) Boxplots of amino acid frequencies among all lineages in this study. The figure was visualized using ggplot2 package v3.3.6 in R2. The HSD post hoc all-pairwise comparisons test was used to test the differences in genomic characters among different groups. Different letters show significant differences between groups with a P value of < 0.05. WAL in abbreviation stands for Wunengiarchaeales associated lineages.

Dataset S1. Genome quality and characteristics statistics of all 163 Micrarchaeota MAGs in this study.

Dataset S2. Geochemical characteristics of all metagenome samples that MAGs were derived from or with reads detected.

Dataset S3. The occurrences of 48 conserved single-copy genes (SCGs) that were used for genome quality assessment in all DPANN genomes.

Dataset S4. Information of all reference genomes used for phylogenomic construction and evolutionary analyses.

Dataset S5. Occurrences of 53 GTDB conserved archaeal markers used for phylogenomic construction.

Dataset S6. Relative abundance of each major Micrarchaeota lineage among all sampling sites with the presence of associated MAGs or reads.

Dataset S7. Summary of metabolic potentials of all major Micrarchaeota lineages illustrated in Fig. 2, and Fig. S8-11.

Dataset S8. Presence of selected genes of interest and pFLAGs pathway completeness among DPANN and other archaeal lineages.

Dataset S9. Occurrences of 49 ribosomal proteins (RPs), 45 arCOGs, and 14 RPs in genome set used for phylogenomic construction of evolutionary inference.

Dataset S10. Presence and absence of evolutionary events of each concerned protein family in ancestral nodes of Micrarchaeota and other lineages.

Dataset S11. Evolutionary models best fitted for the phylogeny of each protein family analyzed in the reconstruction of evolutionary history.

Dataset S12. Counts of evolutionary events among all Micrarchaeota lineages and associated ancestral nodes.

Dataset S13. Counts of evolutionary events and gene gain/loss ratio of each ancestral node. The top 50 hot spots of associated evolutionary events were labeled in red.

Dataset S14. Comparison of proteome sizes on key ancestral nodes in Fig. 4 and Fig. S14.

SI References

1. A. L. Jaffe, C. J. Castelle, C. L. Dupont, J. F. Banfield, Lateral Gene Transfer Shapes the Distribution of RuBisCO among Candidate Phyla Radiation Bacteria and DPANN Archaea. *Molecular Biology and Evolution* **36**, 435–446 (2019).

2. T. Sato, H. Atomi, T. Imanaka, Archaeal Type III RuBisCOs Function in a Pathway for AMP Metabolism. *Science* **315**, 1003–1006 (2007).

3. K. C. Wrighton, *et al.*, RubisCO of a nucleoside pathway known from Archaea is found in diverse uncultivated phyla in bacteria. *ISME J* **10**, 2702–2714 (2016).

4. C. Greening, *et al.*, Genomic and metagenomic surveys of hydrogenase distribution indicate H2 is a widely utilised energy source for microbial growth and survival. *ISME J* **10**, 761–777 (2016).

5. C. J. Castelle, *et al.*, Genomic Expansion of Domain Archaea Highlights Roles for Organisms from New Phyla in Anaerobic Carbon Cycling. *Current Biology* **25**, 690–701 (2015).

6. M. Musfeldt, P. Schönheit, Novel type of ADP-forming acetyl coenzyme A synthetase in hyperthermophilic archaea: heterologous expression and characterization of isoenzymes from the sulfate reducer Archaeoglobus fulgidus and the methanogen Methanococcus jannaschii. *Journal of bacteriology* **184**, 636–644 (2002).

7. J. Glasemacher, A.-K. Bock, R. Schmid, P. Schönheit, Purification and Properties of Acetyl-CoA Synthetase (ADP-forming), an Archaeal Enzyme of Acetate Formation and ATP Synthesis, from the Hyperthermophile Pyrococcus furiosus. *European Journal of Biochemistry* **244**, 561–567 (1997).

8. L.-X. Chen, *et al.*, Metabolic versatility of small archaea Micrarchaeota and Parvarchaeota. *ISME J* **12**, 756–775 (2018).

9. A. Singh, A. Schnürer, J. Dolfing, M. Westerholm, Syntrophic entanglements for propionate and acetate oxidation under thermophilic and high-ammonia conditions. *ISME J* 1–13 (2023).

10. R. A. Gonzalez-Garcia, *et al.*, Microbial Propionic Acid Production. *Fermentation* **3**, 21 (2017).

11. Y.-X. Li, *et al.*, Deciphering Symbiotic Interactions of "*Candidatus* Aenigmarchaeota" with Inferred Horizontal Gene Transfers and Co-occurrence Networks. *mSystems* **6**, e00606-21 (2021).

12. D. Burstein, *et al.*, Major bacterial lineages are essentially devoid of CRISPR-Cas viral defence systems. *Nat. Commun.* **7**, 10613 (2016).

13. D. Burstein, *et al.*, New CRISPR–Cas systems from uncultivated microbes. *Nature* **542**, 237–241 (2017).

14. V. Lombard, H. Golaconda Ramulu, E. Drula, P. M. Coutinho, B. Henrissat, The carbohydrate-active enzymes database (CAZy) in 2013. *Nucl. Acids Res.* **42**, D490–D495 (2014).

15. V. La Cono, *et al.*, Symbiosis between nanohaloarchaeon and haloarchaeon is based on utilization of different polysaccharides. *Proc. Natl. Acad. Sci. USA* **117**, 20223–20234 (2020).

16. U. Sauer, F. Canonaco, S. Heri, A. Perrenoud, E. Fischer, The Soluble and Membrane-bound Transhydrogenases UdhA and PntAB Have Divergent Functions in NADPH Metabolism of Escherichia coli*. *Journal of Biological Chemistry* **279**, 6613–6619 (2004).

17. N. KATO, H. YURIMOTO, R. K. THAUER, The Physiological Role of the Ribulose Monophosphate Pathway in Bacteria and Archaea. *Bioscience, Biotechnology, and Biochemistry* **70**, 10–21 (2006).

18. Y.-N. Qu, *et al.*, Panguiarchaeum symbiosum, a potential hyperthermophilic symbiont in the TACK superphylum. *Cell Reports* **42**, 112158 (2023).

19. C. Bräsen, D. Esser, B. Rauch, B. Siebers, Carbohydrate Metabolism in Archaea: Current Insights into Unusual Enzymes and Pathways and Their Regulation. *Microbiol. Mol. Biol. Rev.* **78**, 89–175 (2014).

20. C. J. Castelle, *et al.*, Biosynthetic capacity, metabolic variety and unusual biology in the CPR and DPANN radiations. *Nat. Rev. Microbiol.* **16**, 629–645 (2018).

21. J. N. Hamm, *et al.*, Unexpected host dependency of Antarctic Nanohaloarchaeota. *Proc. Natl. Acad. Sci. USA* **116**, 14661–14670 (2019).

22. W.-C. Huang, *et al.*, Comparative genomic analysis reveals metabolic flexibility of Woesearchaeota. *Nat. Commun.* **12**, 5281 (2021).

23. A. Flamholz, E. Noor, A. Bar-Even, W. Liebermeister, R. Milo, Glycolytic strategy as a tradeoff between energy yield and protein cost. *Proceedings of the National Academy of Sciences* **110**, 10039–10044 (2013).

24. C. H. Verhees, *et al.*, ADP-dependent phosphofructokinases in mesophilic and thermophilic methanogenic archaea. *Journal of Bacteriology* **183**, 7145–7153 (2001).

25. H. Sakuraba, *et al.*, ADP-dependent Glucokinase/Phosphofructokinase, a Novel Bifunctional Enzyme from the Hyperthermophilic Archaeon Methanococcus jannaschii*. *Journal of Biological Chemistry* **277**, 12495–12498 (2002).

26. P. S. Adam, G. Borrel, S. Gribaldo, Evolutionary history of carbon monoxide dehydrogenase/acetyl-CoA synthase, one of the oldest enzymatic complexes. *Proceedings of the National Academy of Sciences* **115**, E1166–E1173 (2018).

27. D. R. Colman, *et al.*, Phylogenomic analysis of novel Diaforarchaea is consistent with sulfite but not sulfate reduction in volcanic environments on early Earth. *ISME J* **14**, 1316–1331 (2020).

28. S. Neukirchen, I. A. C. Pereira, F. L. Sousa, Stepwise pathway for early evolutionary assembly of dissimilatory sulfite and sulfate reduction. *ISME J* 1–13 (2023).

29. O. V. Golyshina, *et al.*, 'ARMAN' archaea depend on association with euryarchaeal host in culture and in situ. *Nat. Commun.* **8**, 60 (2017).

30. S. Krause, *et al.*, The importance of biofilm formation for cultivation of a Micrarchaeon and its interactions with its Thermoplasmatales host. *Nat. Commun.* **13**, 1735 (2022).

31. B. Kartal, *et al.*, Molecular mechanism of anaerobic ammonium oxidation. *Nature* **479**, 127–130 (2011).

32. Y.-Z. Rao, *et al.*, Metagenomic Discovery of "Candidatus Parvarchaeales"-Related Lineages Sheds Light on Adaptation and Diversification from Neutral-Thermal to Acidic-Mesothermal Environments. *mSystems* **0**, e01252-22 (2023).

33. M. M. M. Kuypers, H. K. Marchant, B. Kartal, The microbial nitrogen-cycling network. *Nat. Rev. Microbiol.* **16**, 263–276 (2018).

34. Dombrowski N, Williams TA, Sun J *et al.* Undinarchaeota illuminate DPANN phylogeny and the impact of gene transfer on archaeal evolution. *Nat Commun* 2020;**11**:3939.

35. Jaffe AL, Castelle CJ, Dupont CL *et al.* Lateral Gene Transfer Shapes the Distribution of RuBisCO among Candidate Phyla Radiation Bacteria and DPANN Archaea. Falush D (ed.). *Molecular Biology and Evolution* 2019;**36**:435–46.

36. Katoh K, Standley DM. MAFFT Multiple Sequence Alignment Software Version 7: Improvements in Performance and Usability. *Molecular Biology and Evolution* 2013;**30**:772–80.

37. Søndergaard D, Pedersen CNS, Greening C. HydDB: A web tool for hydrogenase classification and analysis. *Sci Rep* 2016;**6**:34212.

38. Kolde R. pheatmap: Pretty Heatmaps. R package version 1.0. 12. *CRAN R-project org/package= pheatmap* 2019.

39. Paradis E, Schliep K. ape 5.0: an environment for modern phylogenetics and evolutionary analyses in R. *Bioinformatics* 2019;**35**:526–8.

40. Sauer DB, Wang D-N. Predicting the optimal growth temperatures of prokaryotes using only genome derived features. Valencia A (ed.). *Bioinformatics* 2019;**35**:3224–31.

41. Zeldovich KB, Berezovsky IN, Shakhnovich EI. Protein and DNA Sequence Determinants of Thermophilic Adaptation. *PLOS Computational Biology* 2007;**3**:e5.

42. Team RC. R: A language and environment for statistical computing [Computer software manual]. *Vienna, Austria* 2016.

43. Simes RJ. An improved Bonferroni procedure for multiple tests of significance. *Biometrika* 1986;**73**:751–4.

44. Fd M. Agricolae: statistical procedures for agricultural research. *R package version* 2016;**1**:1–6.

45. Wickham H. *Ggplot2: Elegant Graphics for Data Analysis*. 2nd ed. 2016. Cham: Springer International Publishing : Imprint: Springer, 2016.

46. Van Der Maaten L. Accelerating t-SNE using tree-based algorithms. *The journal of machine learning research* 2014;**15**:3221–45.

47. C. He, *et al.*, Genome-resolved metagenomics reveals site-specific diversity of episymbiotic CPR bacteria and DPANN archaea in groundwater ecosystems. *Nat. Microbiol.* **6**, 354–365 (2021).

48. L. A. Hug, *et al.*, A new view of the tree of life. *Nat. Microbiol.* **1**, 16048 (2016).

49. L. Eme, *et al.*, Inference and reconstruction of the Heimdallarchaeial ancestry of eukaryotes. *Nature* 1–8 (2023).

50. L. E. Valentin-Alvarado, *et al.*, Asgard archaea modulate potential methanogenesis substrates in wetland soil. *Nat. Commun.* **15**, 6384 (2024).

51. Szöllősi GJ, Rosikiewicz W, Boussau B *et al.* Efficient exploration of the space of reconciled gene trees. *Systematic biology* 2013;**62**:901–12.

52. Groussin M, Hobbs JK, Szöllősi GJ *et al.* Toward more accurate ancestral protein genotype–phenotype reconstructions with the use of species tree-aware gene trees. *Molecular biology and evolution* 2015;**32**:13–22.

53. B. P. Hedlund, *et al.*, SeqCode: a nomenclatural code for prokaryotes described from sequence data. *Nat. Microbiol.* **7**, 1702–1708 (2022).
